# Rapid Bacterial Identification, Resistance, Virulence and Type Profiling using Selected Reaction Monitoring Mass Spectrometry

**DOI:** 10.1038/srep13944

**Published:** 2015-09-09

**Authors:** Yannick Charretier, Olivier Dauwalder, Christine Franceschi, Elodie Degout-Charmette, Gilles Zambardi, Tiphaine Cecchini, Chloe Bardet, Xavier Lacoux, Philippe Dufour, Laurent Veron, Hervé Rostaing, Veronique Lanet, Tanguy Fortin, Corinne Beaulieu, Nadine Perrot, Dominique Dechaume, Sylvie Pons, Victoria Girard, Arnaud Salvador, Géraldine Durand, Frédéric Mallard, Alain Theretz, Patrick Broyer, Sonia Chatellier, Gaspard Gervasi, Marc Van Nuenen, Carolyn Ann Roitsch, Alex Van Belkum, Jérôme Lemoine, François Vandenesch, Jean-Philippe Charrier

**Affiliations:** 1 Technology Research Department, Innovation Unit, Marcy l’Etoile, bioMérieux SA, France; 2 UMR 5280, Institut des Sciences Analytiques, Université de Lyon, Lyon 1, 5 Rue de la Doua, 69100 Villeurbanne, France; 3 Centre National de Référence des Staphylocoques, Centre de Biologie et de Pathologie Est, Hospices Civils de Lyon, Bron, France; 4 CIRI, International Center for Infectiology Research; INSERM U111; CNRS UMR5308; University of Lyon; Ecole Normale Supérieure de Lyon; Lyon, France; 5 R&D Microbiologie, La Balme Les Grottes, bioMérieux SA, France; 6 R&D ImmunoAssays, Marcy l’Etoile, bioMérieux SA, France; 7 Technology Research Department, Innovation Unit, Grenoble, bioMérieux SA, France; 8 Microbiology Unit, bioMérieux Inc. Durham, NC, USA; 9 Corporate IP Search and Watch, Intellectual Property, Marcy l’Etoile, bioMérieux SA, France

## Abstract

Mass spectrometry (MS) in Selected Reaction Monitoring (SRM) mode is proposed for in-depth characterisation of microorganisms in a multiplexed analysis. Within 60–80 minutes, the SRM method performs microbial identification (I), antibiotic-resistance detection (R), virulence assessment (V) and it provides epidemiological typing information (T). This SRM application is illustrated by the analysis of the human pathogen *Staphylococcus aureus,* demonstrating its promise for rapid characterisation of bacteria from positive blood cultures of sepsis patients.

Bacterial characterisation has witnessed many technological developments in clinical laboratories in the last decades. Based on a combination of biochemical reactions, microbial identification has been successively miniaturised with API strips^1^ and then automated with bioMérieux VITEK2®, WalkAway MicroScan® (Siemens) or BD Phoenix® systems^2^. Initiated by the pioneering work of Fenselau *et al.*^3^, whole cell mass spectrometry (MS) has now revolutionised microbial identification using Matrix-Assisted Laser-Desorption/Ionisation—Time-Of-Flight (MALDI-TOF) MS. This technology is faster, more accurate and cheaper for large laboratories than conventional biochemical tests^4^. In recent years, MALDI-TOF MS has been broadly accepted in clinical microbiology to become the new Gold Standard for bacterial identification. Being pivotal for the accurate management of patients with infectious diseases, identification has thus been accelerated significantly.

However, the selection of the right antibiotic treatment still requires time-consuming antibiotic susceptibility testing (AST). Despite a certain level of automation achieved over the last decades, obtaining AST results in modern clinical laboratories still requires more than 10 hours following bacterial isolation^5^. In certain instances, AST can be performed by PCR but in most cases AST still relies on conventional microbial growth monitoring in the presence of antibiotics (Fig. 1). As a result, one to three days of delay occur between the initiation of the empirical antimicrobial therapy and the AST result. This contributes to higher in-hospital mortality when the initial therapy turns out to be inappropriate and to additional hospitalisation stay and costs, especially for patients with bloodstream infections^6^.

To overcome the global delay for identification and AST, MALDI-TOF MS identification associated with conventional AST has recently been performed directly on positive blood cultures (Fig. 1), reducing the time to results by two days^7^. Nonetheless, duration of AST remains an important limitation. It has been proposed that MALDI-TOF MS might provide a new tool for AST^8^. However, current literature is limited to specific combinations of bacterial species and antibiotics. Principally, the detection of beta-lactamases through identification of the antibiotic degradation products by MS has been promoted^9^,^10^. Unfortunately, there are no current MS-mediated procedures that cover multiple bacterial species, with multiple antibiotics as well as their respective resistance mechanisms. Finally, the assessment of epidemiological and virulence traits of clinical bacterial isolates is currently limited to reference laboratories. Based on molecular methods (multiplex PCRs, pulsed-field gel electrophoresis, whole genome sequencing, *etc.*), these approaches further prolong the time to results. Obviously, accelerated bacterial characterisation procedures are still needed.

*Staphylococcus aureus* was chosen as a model to demonstrate the feasibility of the MS method we propose here. These common, Gram-positive bacteria can be responsible for both community- and hospital-acquired infections. They constitute one of the major causes of bloodstream infections, in particular when the aetiology is nosocomial^11^. The existence of drug-resistant and/or highly virulent strains reinforces their public health threat. Methicillin-Resistant *S. aureus* (MRSA)^12^ is resistant to nearly all β-lactam antibiotics, due to the low affinity penicillin binding proteins, PBP2a and PBP2c^13^, encoded by the *mec*A and *mec*C genes, respectively. Moreover, *S. aureus* produces a variety of virulence factors encoded by either the core genome or the accessory genome^14^. Among the latter, the toxic shock syndrome toxin 1 (TSST-1) and Panton-Valentine Leukocidin (PVL) toxin are archetypal and their detection calls for anti-toxin therapy in addition to conventional antimicrobial treatment^15^. TSST-1 is involved in menstrual as well as non-menstrual toxic shock syndromes^16^ and PVL is mainly involved in acute primary skin and soft tissue infections^17^, severe bone infections and in frequently lethal necrotising pneumonia^18^. Highly pathogenic MRSA strains expressing PVL have also been described^19^. The detection of both resistance and virulence properties is, therefore, clinically relevant for these organisms.

The development of a targeted MS method operating in the Selected Reaction Monitoring (SRM)^20^ mode is reported here (Fig. 2). The new method shortens the time to results for characterisation of virtually all cultivable microorganisms. We developed generic sample preparation and chromatographic separation methods to be used prior to electrospray triple quadrupole (ESI-QqQ) MS. The method can be applied either directly to bacterial colonies or to positive blood cultures.

## Results

### SRM method development

Using an ESI-QqQ MS, a rapid SRM method was developed for *S. aureus* characterisation (Fig. 2). Proteotypic peptides, *i.e.* specific peptides that characterise at the level of both species and strains, were selected in order to track the following features: *i)* I-peptides to confirm *S. aureus* identification at the species level, *ii)* R-peptides specific to PBP2a and PBP2c to detect MRSA, *iii)* V-peptides characteristic of selected virulence factors (PVL and TSST-1) and *iv)* T-peptides which provide typing information (*e.g.* Protein A peptides). These peptides were selected, according to Bereman *et al.*^21^, by *in silico* digestion of the targeted proteins using UniProtKB sequences and were experimentally verified using strains in the training set. The most appropriate peptides were combined in order to design the SRM method. As a result, detection of 109 peptides from 27 *S. aureus* proteins was performed in a single experiment (Supplementary Table 1).

In practice, a peptide (or precursor) ion was selected by its mass/charge ratio (m/z_1_) using the first quadrupole (Q1) as a filter and was then fragmented in the second quadrupole (q2). A specific fragment ion (m/z_2_) was further selected using the third quadrupole (Q3) as a filter and this was ultimately detected. For each peptide, the three most intense pairs of precursor and associated-fragment m/z settings, named transitions, were successively filtered. The use of Absolute QUAntification peptides (AQUA peptides^22^, *i.e.* peptides synthesised using heavy isotopes) as internal standards was also performed until shown to be redundant (results not shown).

Therefore, the SRM method integrated 474 SRM transitions and, hence, in this format comprised a multiplex of 474 independent assays (Supplementary Table 1): 327 SRM transitions for the 109 targeted peptides, plus 147 transitions for 49 AQUA peptides. During the experimental workflow (Fig. 2a), the peptides were generated from intact bacteria or bacterial particles purified from patient blood culture and fragmented by trypsin digestion. The digests were subsequently resolved using a conventional 2.1 mm-bore chromatographic separation before the SRM analysis (see **Online Methods**). This entire process required only 25 or 40 minutes for the sample preparation from a colony or a positive blood culture, respectively, plus 34 minutes for the liquid chromatography (LC) gradient and the ESI-MS analysis.

### Training set analyses: establishment of strain characterisation parameters at the colony level

The *S. aureus* SRM method was applied to a training set of 38 strains (Supplementary Table 2a), including 29 *S. aureus* strains and 9 isolates belonging to 6 other *Staphylococcus* spp.: *S. haemolyticus, S. simulans, S. capitis, S. saprophyticus, S. epidermidis,* and *S. hominis*. Examples of typical ion mass chromatograms are shown in Fig. 2b and Supplementary Figures 1 to 5. Based on the transition intensities and on the background signals of this training set, a subset of 41 peptides, considered as *bona fide* proteotypic peptides, was selected for clinical evaluation (Fig. 2c). This subset choice was based on the simultaneous detection of 3 transitions per peptide and on the conservation of the normalised ratio between the transition areas (see **Online Methods**). We only included peptides with transition areas higher than 4000 (QTRAP 5500 arbitrary units) and transition retention times with a standard deviation below 0.03. The mean peptide retention time was not taken into account because of weak stochastic variations within a 1.45 min window. We retained peptides having 3 normalised transition ratios with coefficients of variation (CV) inferior to 20%, across the training set. As an average, the CV of the selected transitions was 7.3% (Supplementary Figure 6). These normalised ratios were averaged and used as reference numbers for further detection assessments. Some isobaric and hence interfering ions were also observed, but discarded as having only a single positive transition. Of note, the addition of AQUA peptides^22^ as internal standards was seen as non-compulsory during this training study and was thus omitted in the subsequent evaluation phases.

When multiple peptides were visible for a single protein, *e.g.* PBP2a and PVL, the ratio conservation criteria were extended to all of the transitions related to the same protein (**Online Methods**) and IRV features were detected using correlations with the reference ratios, *i.e.* a combination of the Pearson product-moment correlation coefficient and the slope of the linear regression curve (Supplementary Table 3a). All the strain characters were thus correctly identified by a simple calculation (Table 1). Notably, the distinction between PBP2a and PBP2c was unambiguous as these proteins share only one peptide and were detected by 8 and 5 peptides, respectively. In each strain, when the I-feature, the 2 R-features and the 2 V-features were absent using the reference methods, these features were considered as negative controls. As expected, all these controls were negative using the SRM method (Table 1).

### Testing set analyses: method verification at the colony level

The relevance of the 41 selected peptides was further confirmed using a totally independent testing set of 38 genotypically characterised strains (Supplementary Table 2b), including 29 *S. aureus* and 9 strains from 6 other *Staphylococcus* spp.: *S. haemolyticus, S. simulans, S. capitis, S. epidermidis, S. hominis,* and *S. warneri*. When readily identifiable proteins or peptides were present, the strongest signals were observed for I, R, and T-peptides and the weakest signals for V-peptides (see Supplementary Experimental data). With regard to identification, methicillin susceptibility and toxin detection, the results were in complete agreement with the strain characters previously determined by molecular or phenotypic methods, except for a single case where a non-detectable mutated peptide of TSST-1 was involved (Table 1 and Supplementary Table 3b).

### Reproducibility assessment

The reproducibility of the SRM method was tested using 3 independent assays for the same 12 strains (Supplementary Table 2e). The 3 analyses were initiated from independent subcultures of all strains. For each repeat, the entire protocol from culture to sample preparation and LC-ESI-QqQ MS analysis was, therefore, run independently. All triplicate results were consistent (Table 1 and Supplementary Table 3e), demonstrating efficient qualitative detection without the need for an internal standard. The quantity of processed bacteria (*i.e.,* 1 mL at 4 McF per colony or 0.8 mL of positive blood culture broth) ensured reproducible qualitative detection without a need for an internal standard.

Another way to assess the robustness of the SRM method is to observe the pattern stability at the feature level. Indeed, the method should be robust to the pattern distortion anticipated when a peptide is mutated or post-translationally modified. In either one of these cases, the peptide mass changes rendering the corresponding transitions undetectable by the SRM method. Except for TSST-1, the number of peptides searched for a single feature (9, 8, 5 and 4 peptides for I, R-PBP2a, R-PBP2c and V-PVL, respectively) overcame this issue. Moreover, the transitions were normalised for each peptide (see **Online Methods**), and the normalised transitions were compared by correlation calculation to averaged ratios, learned during the train set study. As an example, Fig. 3 displays the correlation used for *S. aureus* identification (Fig. 3a) and PBP2a detection (Fig. 3b) in Strain_026. When a feature was positive, both the Pearson product-moment correlation coefficient, r (Fig. 3c), and the slope (Fig. 3d) were approximately 1. Other examples of experimental correlations are displayed on Supplementary Figure 7 and support the stability of feature patterns. This provided a simple but powerful statistical value for the estimation of the ratio conservation between transitions for all of the peptides characteristic of a feature. Having a minimal peptide number per feature and using a 30% tolerance for both the Pearson product-moment correlation coefficient and the slope was seen as efficient for robust and reproducible feature detections (Supplementary Table 3 and Supplementary Figure 8).

### Clinical evaluation: Blinded characterisation of *S. aureus* strains directly from a colony

The initial clinical evaluation using the SRM method was performed on a blinded subset of 20 well-characterised *S. aureus* strains selected by the French National Reference Center for Staphylococci. As representative of the worldwide diversity of *S. aureus*, this collection of 20 strains included isolates of highly prevalent MSSA and MRSA lineages (including community-acquired MRSA clones), either expressing TSST-1 alone, together with PVL or not at all. As additional controls, genetically-engineered derivatives of the above strains in which *tst* or *pvl* genes had been knocked out (Supplementary Table 2c) were also analysed. The IRV results were 100% accurate, in perfect agreement with molecular and phenotypic methods (Table 1, Supplementary Table 3c and Supplementary Figure 8).

### Typing information

In addition to identification, resistance and virulence, the SRM method brought interesting typing information from the same analysis. As previously demonstrated by the use of DNA microarrays suited for *S. aureus* resistance and virulence factor detection^23^, a dedicated algorithm using a combination of sequences could probably be set up to assign certain peptides to known clonal complexes or sequence types of well-known multi-locus sequence typing schemes. A preliminary attempt to evaluate this possibility was based on the combination of T-peptides along with Protein A and I-peptides. It allowed a clear distinction between major lineages and unrelated strains (Supplementary Table 4), demonstrating the promising potential of the technology. However, the existence of repetitive sequences in Protein A may lead to ubiquitous peptides after digestion by trypsin. Initially, T-peptides from Protein A were selected by analogy with the *spa* typing method, based on sequencing of the polymorphic region of the Protein A gene (*spa*)^24^. Indeed, the polymorphism and the repeat structure of the *spa* gene enable a simultaneous indexation of both micro- and macrovariations. Standardised *spa* type nomenclature has thus been established to study both local and global outbreaks. The same work remains to be done at the peptide level to definitively establish the T-peptide list in order to achieve an IRVT-SRM method.

### Clinical evaluation: Blinded characterisation of strains in positive blood cultures

SRM was subsequently performed on 14 blinded aerobic blood cultures after growth detection and *S. aureus* species identification (see **Online Methods**). These blood cultures were from 14 different patients hospitalised in the Lyon University Hospital (Supplementary Table 2d). Negative bottles were ruled out from the study since only positive bottles are considered for further analysis in a routine clinical microbiology laboratory. The bacterial content of the positive bottle was analysed using Gram staining followed by a MALDI-TOF MS-based identification. The SRM method was thus used for in-depth characterisation of the bacterial features of clinical interest. The IRV results were again in full agreement with the clinical laboratory data (Table 1 and Supplementary Table 2d).

## Discussion

**Figure 1:**
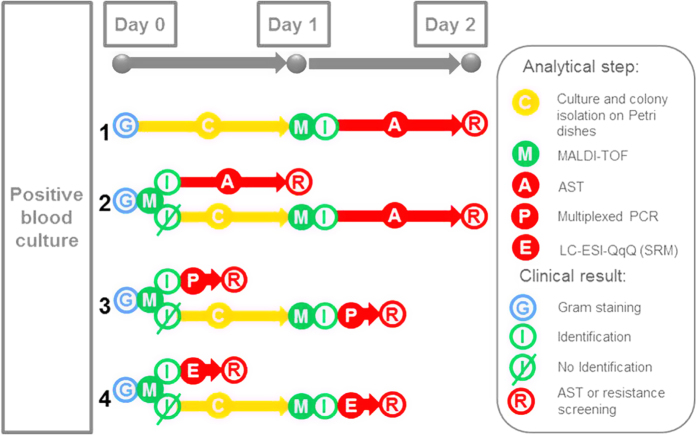
Workflow strategies for positive blood culture analysis. Case 1 is the usual workflow using Gram staining (1–4 h), isolation culture on Petri dishes (16–24 h), MALDI-TOF MS identification (15 min-1 h) and conventional AST (6–24 h). Gram staining is performed directly from positive blood cultures at day 0, but microbial identification is obtained at day 1 and, generally, AST at day 2. For cases 2–4, Gram staining and MALDI-TOF MS identification are performed at day 0, but when MALDI-TOF MS identification is inconclusive, isolation culture on Petri dishes is required (second line in each cases). Case 2 (upper line): following identification, conventional AST is performed^7^. Identification is obtained at day 0 and AST at the end of day 0, or at day 1. Case 3 (upper line): AST is replaced by a multiplexed PCR resistance screening (2 h) according to Klein *et al*.^27^. Identification and resistance information are available at day 0. However PCR could be confounded by polymicrobial cultures which happen in 5–10% of all cases. Case 4 (upper line): SRM (1 h) replaces PCR. Identity and resistance information are available at day 0. Notably, the results are less sensitive to the confounding effects of a polymicrobial culture, since proteins are detected without molecular amplification. Only resistance proteins from the dominant microorganism are detectable. High multiplexing capabilities of LC-ESI-QqQ MS in SRM mode allows additional information: molecular confirmation of closely-related species as well as virulence and typing information. The same workflow and reagents are used irrespective of the species; only an appropriate SRM method has to be selected.

Our results demonstrate the efficiency of SRM in rapidly achieving a detailed IRV characterisation of *S. aureus* from colonies or monomicrobial positive blood cultures, notably for the specific detection of MRSA. Illustrated by Fig. 1, the method is adapted to speed up today’s bacteremic septic patient management. IRV results may be transmitted to the clinician the same day the blood culture is flagged positive (*i.e* day 0). The empiric treatment may thus be optimised two days earlier than when using conventional technologies. Currently, the detection of MRSA may take up to 3 days after a blood culture turns positive. During this period antibiotherapy is usually empiric and an elevated local MRSA prevalence generates a clinical dilemma: does one use M penicillin or vancomycin as first line treatment? As shown by others^25^,^26^, MALDI-TOF MS may quickly identify a pathogen in positive blood cultures. When combined with rapid AST, preferably also performed directly on positive blood culture broth, this may accelerate the choice for a more targeted antibiotic for treatment and thus reduce hospitalisation costs^25^,^27^. However, to date AST results are generally reported the day after the blood culture becomes positive. An alternative option may be the use of ELISA or PCR strategies, using short protocols and multiplexed tests providing both information on species nature and presence of resistance genes^27^. However, multiplexed assays are usually expensive whereas SRM provides an attractive and cost-effective alternative approach that generates information on species nature and the presence of resistance determinants within 70 min of a blood culture signalling positive. In addition, virulence and typing information becomes available within the same time-frame. Consequently, SRM can also provide epidemiological information on the possible spread of resistant strains and/or resistance markers.

Workflow implications for microbiological laboratories are also of utmost importance. Thus, a cumbersome method to repetitively calibrate the quantity of bacteria was excluded. A simple preparation procedure for roughly the same bacterial biomass (*e.g.* 1 mL of a bacterial suspension at 4 McF or 0.8 mL of positive blood culture broth) was developed. Moreover, to preserve workflow simplicity, specific reagents such as antibodies or steps costly to automate, such as centrifugation, were eliminated. A generic and possibly automatable sample preparation protocol has been targeted, using simple ultrasound disruption for bacterial lysis and trypsin digestion for peptide generation. Sensitivity and specificity could potentially be improved using SISCAPA^28^ or similar protein– or peptide–capture methodology, but will encumber the versatility of the approach. For the same reason, the use of internal standards such as AQUA^29^, QCONCAT^30^ or PSAQ^31^ was considered but ultimately not applied. The need of special mixtures of internal calibrants for different clinical questions would have been very cumbersome in routine. Simplicity and minimal time to results have driven protocol choices without jeopardising quality.

**Figure 2:**
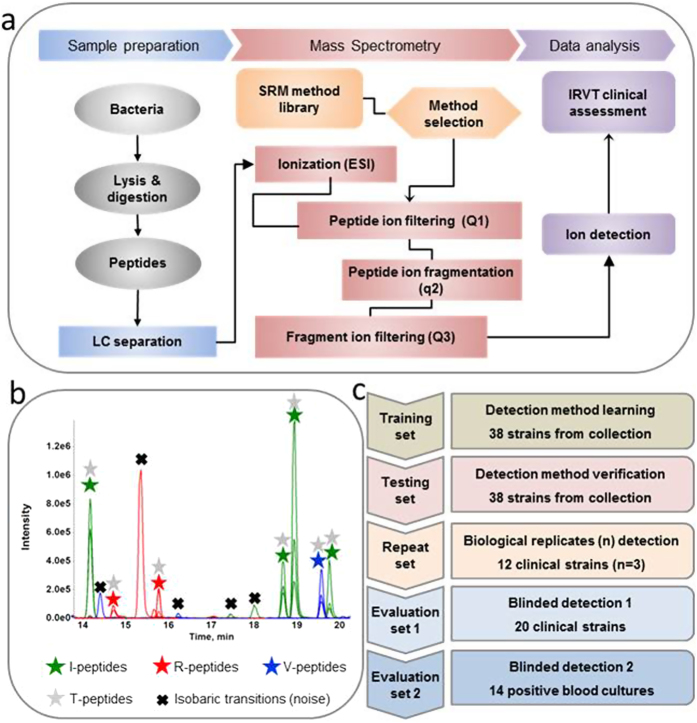
SRM method development for Identification, Resistance, Virulence and Type profiling. (**a**) Sample analysis workflow. Bacteria were lysed and proteins digested. The mixture of peptides was separated by conventional chromatography and analysed by ESI-QqQ MS in SRM mode. After electrospray ionisation (ESI), specific precursor ions were filtered in quadrupole Q1, fragmented in quadrupole q2, filtered in quadrupole Q3 and detected. For each peptide, 3 transitions (pairs of precursor and fragment ion mass/charge) were monitored using a 3-minute window targeting the expected elution time. (**b**) Typical ion chromatogram acquired during a single injection. Extracted-ion chromatogram from peptides for *S. aureus* identification (I-peptides: green stars), resistance screening (R-peptides: red stars), virulence screening (V-peptides: blue stars) and typing (T-peptides: grey stars). All I, R and V-peptides contain typing information and could be used as T-peptides. Isobaric transitions (biological noise) are identified by black cross. (**c**) Validation strategy method. A training set was used to develop the method, an independent testing set permitted the method verification and two blinded evaluation sets allowed to verify the method using blinded clinical samples, either pure cultures or unprocessed positive blood cultures. In addition to these two evaluation sets, a repeat set was studied to assess robustness.

**Figure 3:**
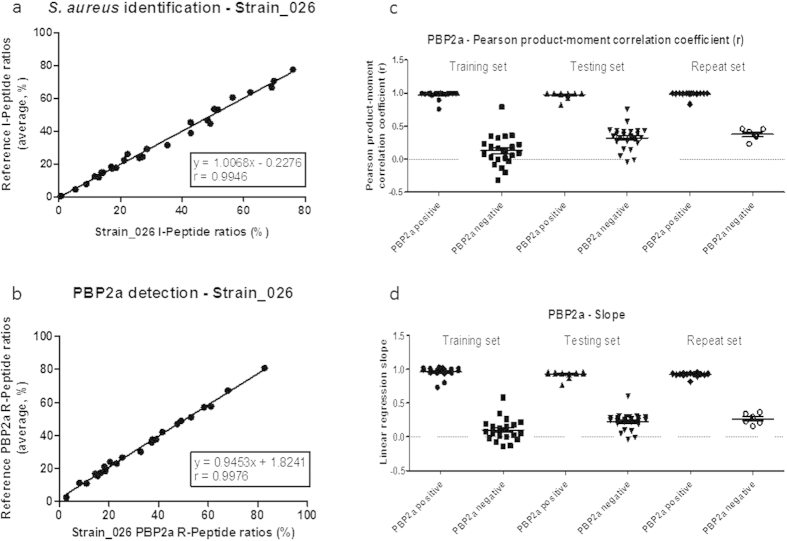
Features detection using linear regression statistics. (**a**) Linear regression curves between Strain_026 and reference normalised ratios for *S. aureus* positive identification feature. (**b**) Linear regression curves between Strain_026 and reference normalised ratios for PBP2a positive detection feature. (**c**) Pearson product-moment correlation coefficients (r) for PBP2a feature in training, testing and repeat sets. (**d**) Linear regression slopes for PBP2a feature in training, testing and repeat sets.

The method and system robustness was furthermore privileged by choosing conventional 2.1 mm internal diameter bore chromatography column, instead of nano-chromatography equipment frequently adopted in proteomic studies. Nano-chromatography might be employed to enhance SRM sensitivity, addressing samples with a lower bacterial charge, but the difficulty to assure reliable detection (retention time variation, peak shape alteration, etc.) in routine^32^ as well as longer analysis times and complex troubleshooting was considered as incompatible with clinical use. Likewise, an ESI-QqQ MS was preferred over high resolution instruments broadly used in proteomics research. The workhorse ESI-QqQ MS used in these studies has demonstrated its effectiveness in the routine detection and quantitation of chemicals and metabolites with high reliability, and was thus favoured for peptides. Consequently, the signal showed reproducible chromatographic peaks (Fig. 2b and Supplementary Figures 1 to 5) enabling analytical and clinical validation.

Automation of data processing was also evaluated. Recently, advanced approaches have been proposed to meet quantification during large proteomic studies^33^,^34^,^35^,^36^, however, a less complex strategy was preferred here and its efficiency was demonstrated for SRM qualitative measurements. Here the reliance is on simple physical parameters and a broadly accepted statistical analysis. Cut-off values for peak intensity, retention time and ratio conservation were defined via the training and testing set and experimentally verified by 3 independent sets of analyses. Finally, from a clinical perspective, the effectiveness of the method relies on the regular analysis of positive and negative controls using well-characterised strains.

The flexible methodology described here using a protocol with simple steps, proven instrumentation and statistically sound data analysis can be easily expanded towards other species of microorganism with different resistance and virulence features. There are no obstacles to multiplexed inclusion of other resistance traits, such as β-lactamase or porin production in Gram negatives^37^, if the adequate bacterial cell quantity is provided. The SRM method indeed requires at least 10^7^ colony-forming units, supporting the analysis of “biologically amplified” bacteria found in isolated colonies, or positive blood cultures. Unlike PCR, however, these techniques are not susceptible to detection of low levels of dead bacteria, which can lead to false positive results and potential overtreatment of patients. SRM provides a direct method, measuring only significantly expressed proteins. It enables a phenotyping-like pattern and circumvents the gene regulation question, sometimes difficult to predict by nucleic acid-mediated methods. When appropriately developed, protein measurements by ELISA could offer the same benefits and be more sensitive than SRM, but remain dependent on obtaining both optimal antibodies and antigens. ELISAs, thus, tend to require a much longer development time than SRM^38^.

Finally, to be exhaustive, special attention was paid to matrix issues frequently reported in proteomic studies using human serum^39^. Indeed, SRM may be affected by isobaric transitions from non-relevant but abundant molecules, *i.e.* the matrix. We overcame this common pitfall by the careful selection of transitions during the training set. Only transitions having CVs of normalised ratios below 20% were selected. These transitions were seen as having minimal alterations due to sample matrix effects on the testing, repeat and evaluation sets of strains (Fig. 2c). Stochastic variations, attributed to matrix effects, were occasionally observed, but the matrix noise was weak and easily ruled out by the ratio conservation rules. The targeted bacterial proteins were probably not in the ultra low concentration range as they are not further diluted in natural extra-matrices, such as cancer biomarkers in serum. It was thus easier to find peptides not contaminated by non-relevant molecules present in the bacterial digests, without the necessity of sample fractionation.

Actually, the only observed weakness of the method was an undetectable TSST-1 production in a strain where the targeted peptide was mutated. The current SRM method can easily be modified to include the transitions of the mutated peptide (data not shown), but will still be impinged if other mutations occur. Being highly specific based on m/z, SRM is clearly sensitive to mass changes due to single amino acid mutations. The detection of several peptides per feature circumvents this issue, except if the production-level and the small length of the protein constrain the peptide selection to one peptide, such as for TSST-1. Obviously, other molecular methods, including for instance PCR, may be equally sensitive to mutation.

## Conclusion

The SRM method enables the in-depth characterisation of *S. aureus* strains in 60–80 minutes using a single, multiplexed analysis. Not only have clinical *S. aureus* strains been successfully identified but also their resistance to methicillin (due either to PBP2a or PBP2c) has been correctly determined. In addition, the initial experiments identifying toxins (PVL and TSST-1, two relevant toxins chosen for this proof of concept study) have been extremely encouraging, showing characterisation of each according to expectation. With the exception of the undetectable mutated TSST-1 peptide, all the IRV traits were detected in the 110 different strains with 100% specificity and 100% sensitivity. The fact that multiple marker peptides were used per protein, except for TSST-1, provides high-level confidence in the test results. Moreover, potential information for epidemiological typing is accessible after the same analysis.

As it provides faster microbial characterisation, SRM should help to reduce time of empirical antimicrobial therapy, allowing earlier administration of the most suitable drugs as required. The SRM methods can be easily adapted to other species and their resistance and virulence proteins. This suggests a highly relevant approach in septic shock diagnosis where delay of adequate treatment is correlated with the mortality rate^40^. Appropriate antibiotic treatment facilitated by SRM may therefore be an effective means for combating microbial antibiotic resistance.

SRM illustrates the potential clinical applicability of mass spectrometry in developing personalised patient treatment, particularly in the context of the emergence of multidrug resistance and of the current paucity of alternative therapies.

## Online Methods

### Reagents and chemicals

Acetonitrile and water (LC-MS grade) were obtained from Fisher Scientific (Strasbourg, France). Formic acid, dithiotreitol (DTT), iodoacetamide (IAA), ammonium bicarbonate and porcine trypsin were purchased from Sigma-Aldrich-Fluka (Lyon, France). Blood culture bottles and agar plates for bacterial isolation and culture were obtained from bioMérieux (Marcy L’Etoile, France). Peptides were synthesised using Fmoc chemistry on a MultiPep RS peptide synthesiser from Intavis Bioanalytical Instruments AG (Koeln, Germany).

### Clinical samples storage and culture

Seventy six clinical strains were obtained from the bioMérieux culture collection and were identified by biochemical methods using the VITEK2® instrument. The *mec*A*, mec*C, *luk*F*, luk*S and *tst* genes were detected by PCR as previously described^41^,^42^,^43^ or with the following primer sets: *mec*C-F, 5′-AGC AAG CAA TAG AAT CAT CAG ACA A-3′; *mec*C-R, 5′-CAA ATC TTG CAT ACC TTG CTC AAA-3′; *mec*C-P, 5′-FAM-CTA ATG GTA ATG CAA TGC GGG CAA AAA A-TAMRA-3′.

Twenty clinical strains were obtained from the French National Reference Center for Staphylococci (Lyon, France) and were characterised by their toxin gene profile, *spa* typing and multi locus sequence typing (MLST) as previously described^44^,^45^,^46^.

All strains were stored in glycerol solutions at −80 °C. Before use, two successive cultures were performed on Columbia Blood agar plates (bioMérieux, Marcy l’Etoile, France) at 37 °C for 18 h in aerobic conditions.

Fourteen positive aerobic blood cultures (BacT/ALERT® SN, bioMérieux, Marcy l’Etoile, France) were obtained from 14 different patients, 8 men and 6 women, mean age 59.7 years old, hospitalised in Lyon’s Hospital University (Hospices Civils de Lyon, France) from intensive care or emergency units (48%), medical units (30%) or surgical units (22%). Blood cultures were classically reported as positive by growth detection using the BacT/ALERT® 3D instrument (bioMérieux). After growth detection, the blood cultures demonstrated the presence of Gram positive cocci in clusters, identified as *S. aureus* upon subculture by MALDI-TOF mass spectrometry (VITEK-MS, SARAMIS®, bioMérieux, Marcy l’Etoile, France). The blood cultures were stored at 2–8 °C until SRM analysis. Isolated strains were also characterised according to their toxin gene profiles, *spa* type and MLST (see above).

### Ethics statement

The study protocol was in accordance with the ethical guidelines approved by the Hospices Civils de Lyon (HCL)’s committee and French laws. Moreover, informed consent was obtained from all participants. Blood samples were rendered anonymous before shipment from the hospital.

### Blood culture sample preparation

Positive bottles were processed according to Fothergill *et al.*^44^. Briefly, human blood cells were lysed for 3 min at room temperature using an alkaline buffer containing detergents (0.6% polyoxyethylene 10 oleoyl ether [Brij 97] in 0.4M[3-(cyclohexylamino)-1-propane sulfonic acid] [CAPS], pH 11.7) prior to filtration. Micro-organisms were collected on a 0.45 μ pore-size polyethersulfone membrane (Millipore, Billerica, MA) and washed using 200 μL of buffer (50 mM NH_4_HCO_3_, pH 8.0). The process was performed in less than 15 min.

### Colony sample preparation

Colonies grown on Petri dishes were suspended in saline buffer (NaCl 0.45%) at 4 McF. One mL was centrifuged at 8,000 g during 15 min and the pellet was resuspended in 200 μl of buffer (50 mM NH_4_HCO_3_, pH 8.0).

### Bacterial lysis and digestion

Positive blood culture material or bacterial suspensions were collected into 1.5 mL Eppendorf tubes, containing a mixture of glass beads and 100 μL of 50 mM NH_4_HCO_3_, 5 mM DTT, pH 8.0. The tubes were placed on an ultrasound probe (Hielscher Ultrasonics GmBH, Teltow, Germany) and the cells were disrupted in 5 min. Proteins in the lysates were alkylated using 12.5 mM iodoacetamide during 5 min in the dark at room temperature. Trypsin (10 μg) was added to the samples and the digestion was performed in a heating block at 50 °C during 15 min. The tryptic digestion was stopped by acidifying the samples with 0.5 μL formic acid. Samples were directly analysed or stored at −20 °C until analysis.

### Liquid chromatography and mass spectrometry analysis

An Ultimate 3000 series HPLC instrument including a binary pump and an autosampler (Dionex, Sunnyvale, CA) hyphenated to a hybrid triple quadrupole/linear ion trap mass spectrometer (QTRAP 5500, AB Sciex, Foster City, CA) equipped with a Turbo V ion source was used for LC-MS analyses. Instrument control, data acquisition and processing were performed using Analyst 1.5.1 software. LC separation was carried out on a XBridge BEH C18 column (100 * 2.1 mm, particle size 3.5 μm, porosity 130 Å) from Waters (Milford, MA). Elution was performed at a flow rate of 300 μL/min with water containing 0.1% (v/v) formic acid as solvent A and acetonitrile containing 0.1% (v/v) formic acid as solvent B. An isocratic step at 2% solvent B during 3 min was followed by a 31-min linear gradient from 2% to 45.4% solvent B. The mass spectrometer was initially tuned and calibrated using polypropylene glycol, reserpine, and the Agilent Tuning Mix (all AB Sciex, Foster City, CA) according to the manufacturer’s instructions. Each peptide was detected using scheduled SRM with 3 min windows. Q1 resolution was adjusted to 0.7 ± 0.1 atomic mass unit full width at half maximum (amu-fwhm), referred to as unit resolution. Q3 was also set to unit resolution. MS analysis was carried out in positive ionisation mode using an ion spray voltage of 5500 V. The nebuliser and the curtain gas flows were set at 45 psi using nitrogen. The QTRAP 5500 Turbo V ion source was operated at 550 °C with the auxiliary gas flow (nitrogen) set at 40 psi.

To ensure the SRM assay performance, a synthetic peptide (LSEPAELTDAVK, from the human Prostate Specific Antigen protein) dissolved in water was regularly analysed. Thanks to this reference peptide, chromatographic separation, retention time, intensity signal, transition relative intensities were continuously inspected. Additionally, blank samples were analysed to rule out memory effects, after every bacterial sample during SRM developments and every ten samples during strain analyses. ESI-QqQ MS cleaning operations were scheduled on the basis of this procedure and were performed at the end of each sample set.

### Data analysis

MultiQuant^TM^ 2.1 software (AB Sciex), using the integration algorithm Summation for peak integration was used for SRM data analysis. Peak integration parameters were set with a Gaussian smooth width of 1.0 point, a summation window of 10 s, a re-centering window of 20 s around the expected retention time and a 10% noise level for baseline setting.

Peptide detection was considered positive if 3 transitions per peptide were simultaneously detected and if the peak area ratios between the transitions were conserved.

In practice, the following criteria were adopted for a detection of a single peptide: 1) all the 3 transition areas were higher than 4000 (QTRAP 5500 arbitrary units); 2) the standard deviation of the retention time (min) from the 3 transitions of the same peptide was lower than 0.03; 3) the Pearson product-moment correlation coefficient of the linear regression curve between the sample and the reference for the 3 normalised transition areas was higher than 0.9; 4) the slope of the linear regression curve was between 0.9 and 1.1.

The transition area normalisation was calculated in each sample using the following equation:


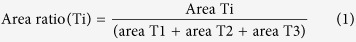


Where T1, T2 and T3 represent each of the 3 transitions and Ti a single one of the 3; an average area ratio was estimated on the basis of positive samples from the training set.

When multiple peptides were detected for a feature (*e.g.* for *S. aureus* identification or for PBP2a detection), the ratio conservation criteria were extended to all the peptides characteristic of the feature. Equation (1) was applied for each peptide and the correlation curve between all normalised areas and the normalised average for areas from the training set was used as a simple objective criterion (see Supplementary Figure 6). In practice, the feature was assumed to be detected if 3) the Pearson product-moment correlation coefficient was higher than 0.7 and 4) if the slope was between 0.7 and 1.3 (see Supplementary Figure 7). Moreover, 5) a minimal number of peptide criterion was added. This number was two for *S. aureus* identification, three (2+1) for PBP2a and PBP2c detection (one peptide is shared between PBP2a and PBP2c) and one for PVL and TSST-1 (PVL and TSST-1 have only 4 and 1 peptides, respectively, in the SRM method).

### SRM assays construction

Peptides were selected from *in silico* digestion of the targeted proteins using UniProtKB sequences. SRM assays were created using synthetic peptides, synthesised using Fmoc chemistry on a MultiPep RS peptide synthesiser (Intavis Bioanalytical Instruments, Koeln, Germany). Peptide purity was established using HPLC and a 6540 Q-TOF MS (Agilent, Santa Clara, CA) to be better than 95%. The 3 most intense transitions were selected to build the SRM method.

## Additional Information

**How to cite this article**: Charretier, Y. *et al.* Rapid Bacterial Identification, Resistance, Virulence and Type Profiling using Selected Reaction Monitoring Mass Spectrometry. *Sci. Rep.* **5**, 13944; doi: 10.1038/srep13944 (2015).

## Supplementary Material

Supplementary Information

Supplementary Dataset 1

## Figures and Tables

**Table 1:** Identification, resistance and virulence results using SRM *versus* reference methods.

		Reference methods: MALDI-TOF for identification, PCR test for mecA, mecC, lukF, lukS and tst genes detection									
		Train set		Test set		Blind Evaluation set 1		Blind Evaluation set 2		Repeat set 1	
Experimental method: SRM profiling		Positive controls	Negative controls	Positive controls	Negative controls	Positive controls	Negative controls	Positive controls	Negative controls	Positive controls	Negative controls
*S. aureus* strains	Positive results	29	0	29	0	20	0	14	0	32	0
	Negative results	0	9	0	9	0	0	0	0	0	2
PBP2a producing strains	Positive results	16	0	14	0	14	0	1	0	17	0
	Negative results	0	22	0	24	0	6	0	13	0	17
PBP2c producing strains	Positive results	8	0	8	0	2	0	0	0	6	0
	Negative results	0	30	0	30	0	18	0	14	0	28
PVL producing strains	Positive results	9	0	8	0	5	0	0	0	9	0
	Negative results	0	29	0	30	0	15	0	14	0	25
TSST-1 producing strains	Positive results	1	0	4	0	4	0	0	0	9	0
	Negative results	0	37	1^a^	33	0	16	0	14	0	25

a A strain harboring TSST-1 gene was seen as negative for TSST-1 by SRM. However, sequencing detected a mutation in the proteotypic TSST-1 peptide selected for the SRM method. LPTPIELPLK peptide was mutated into LLTPIELPLK peptide. The corresponding precursor masses are different and the LLTPIELPLK peptide detection was impossible with the SRM method. Nevertheless, the current SRM method should be easily modified to integrate the transitions of the mutated peptide (data not shown).
