# Supplementary material for: Rapid Bacterial Identification, Resistance, Virulence and Type Profiling using Selected Reaction Monitoring Mass Spectrometry
Source: Sci Rep. 2015 Sep 9;5:13944. doi: 10.1038/srep13944 (PMC4563557; doi:10.1038/srep13944)
Supplement: Supplementary Information [file srep13944-s1.pdf]

Rapid Bacterial Identification, Resistance and Virulence profiling using Selected Reaction Monitoring Mass Spectrometry

Yannick Charretier, Olivier Dauwalder, Christine Franceschi, Elodie Degout-Charmette, Gilles Zambardi, Tiphaine Cecchini, Chloe Bardet, Xavier Lacoux, Philippe Dufour, Laurent Veron, Hervé Rostaing, Veronique Lanet, Tanguy Fortin, Corinne Beaulieu, Nadine Perrot, Dominique Dechaume, Sylvie Pons, Victoria Girard, Arnaud Salvador, Géraldine Durand, Frédéric Mallard, Alain Theretz, Patrick Broyer, Sonia Chatellier, Gaspard Gervasi, Marc Van Nuenen, Carolyn Ann Roitsch, Alex Van Belkum, Jérôme Lemoine, François Vandenesch, Jean-Philippe Charrier

Supplementary Information

Supplementary Table 1: SRM method for S. aureus multiplexed characterisation 5

Supplementary Table 2: Datasets characterisation using refrence methods 47

Supplementary Table 2a: Train set characterisation 47

Supplementary Table 2b: Test set characterisation 49

Supplementary Table 2c: characteristics of Staphylococcus aureus blind evaluation set 1 50

Supplementary Table 2d: characteristics of Staphylococcus aureus blind evaluation set 2 52

Supplementary Table 2e: repeat set characterisation 53

Supplementary Table 3: Results 54

Supplementary Table 3a: Train set results 54

Supplementary Table 3b: Test set results 56

Supplementary Table 3c: Results of blind evaluation with set 1 strains 58

Supplementary Table 3d: Results of blind evaluation with set 2 strains 59

Supplementary Table 3e: Results of repeat set strains 60

Supplementary Table 4: Typing information, Staphylococcus aureus blind evaluation set 1 62

Supplementary Figure 1: Screenshot of Strain_067, analysed in blind conditions and identified as S. aureus, expressing both Penicillin Binding Protein 2a (PBP2a) and Panton-Valentine Leukocidin (PVL), but neither Penicillin Binding Protein 2c (PBP2c), nor Toxic Shock Syndrome Toxin 1 (TSST-1). 63

A - Extracted ion chromatogram of peptides used for identification. 63

B - Extracted Ion chromatogram of peptides used for Penicillin Binding Protein 2a (PBP2a) detection. 63

C - Extracted Ion chromatogram of peptides used for Penicillin Binding Protein 2c (PBP2c) detection. 64

D - Extracted ion chromatogram of peptide used for Panton-Valentine Leukocidin (PVL) detection. 64

E - Extracted ion chromatogram of peptide used for Toxic Shock Syndrome Toxin 1 (TSST-1) detection. 65

F - Total extracted ion chromatogram 65

Supplementary Figure 2: Screenshot of Strain_080, analysed in blind condition, identified as S. aureus, expressing neither Penicillin Binding Protein 2a (PBP2a), Penicillin Binding Protein 2c (PBP2c), Panton-Valentine Leukocidin (PVL) nor Toxic Shock Syndrom Toxin 1 (TSST-1). 66

A - Extracted ion chromatogram of peptides used for identification. 66

B - Extracted Ion chromatogram of peptides used for Penicillin Binding Protein 2a (PBP2a) detection. 66

C - Extracted Ion chromatogram of peptides used for Penicillin Binding Protein 2c (PBP2c) detection. 67

D - Extracted ion chromatogram of peptide used for Panton-Valentine Leukocidin (PVL) detection. 67

E - Extracted ion chromatogram of peptide used for Toxic Shock Syndrome Toxin 1 (TSST-1) detection. 68

F - Total extracted ion chromatogram 68

Supplementary Figure 3: Screenshot of Strain_040, analysed in blind conditions, and identified as Staphylococcus hominis and expressing neither Penicillin Binding Protein 2a (PBP2a) neither Penicillin Binding Protein 2c (PBP2c), nor Panton-Valentine Leukocidin (PVL), nor Toxic Shock Syndrome Toxin 1 (TSST-1). 69

A - Extracted ion chromatogram of peptides used for identification. 69

B - Extracted Ion chromatogram of peptides used for Penicillin Binding Protein 2a (PBP2a) detection. 69

C - Extracted Ion chromatogram of peptides used for Penicillin Binding Protein 2c (PBP2c) detection. 70

D - Extracted ion chromatogram of peptide used for Panton-Valentine Leukocidin (PVL) detection. 70

E - Extracted ion chromatogram of peptide used for Toxic Shock Syndrome Toxin 1 (TSST-1) detection. 71

F: Total extracted ion chromatogram......71

Supplementary Figure 4: Screenshot of Strain_071, analysed in blind conditions, and identified as Staphylococcus aureus, expressing Penicillin Binding Protein 2a (PBP2a) and Toxic Shock Syndrome Toxin 1 (TSST-1), but neither Penicillin Binding Protein 2c (PBP2c), nor Panton-Valentine Leukocidin (PVL). ...72

A - Extracted ion chromatogram of peptides used for identification 72

B - Extracted Ion chromatogram of peptides used for Penicillin Binding Protein 2a (PBP2a) detection. 72

C - Extracted Ion chromatogram of peptides used for Penicillin Binding Protein 2c (PBP2c) detection. 73

D - Extracted ion chromatogram of peptide used for Panton-Valentine Leukocidin (PVL) detection. 73

E - Extracted ion chromatogram of peptide used for Toxic Shock Syndrome Toxin 1 (TSST-1) detection. 74

F: - Total extracted ion chromatogram 74

Supplementary Figure 5: Screenshot of Strain_078, analysed in blind conditions, and identified as Staphylococcus aureus, expressing Penicillin Binding Protein 2c (PBP2c) but neither Penicillin Binding Protein 2a (PBP2a), nor Panton-Valentine Leukocidin (PVL), nor Toxic Shock Syndrome Toxin 1 (TSST-1). 75

A - Extracted ion chromatogram of peptides used for identification 75

B - Extracted Ion chromatogram of peptides used for Penicillin Binding Protein 2a (PBP2a) detection....75

C - Extracted Ion chromatogram of peptides used for Penicillin Binding Protein 2c (PBP2c) detection. 76

D - Extracted ion chromatogram of peptide used for Panton-Valentine Leukocidin (PVL) detection. 76

E - Extracted ion chromatogram of peptide used for Toxic Shock Syndrome Toxin 1 (TSST-1) detection. 77

F - Total extracted ion chromatogram. 77

Supplementary Figure 6: Frequency distribution of the coefficient of variation from the normalised area ratios across the training set....78

Supplementary Figure 7: feature detection in Strain_093....79

Supplementary Figure 7a: S. aureus identification based on peptide ratio conservation. 79

Supplementary Figure 7b: Penicillin Binding Protein 2a (PBP2a) detection based on peptide ratio conservation. 80

Supplementary Figure 8: Slope and the Pearson product-moment correlation coefficients (r) from the linear regression across the data sets 82

Supplementary Figure 8a: *S. aureus* identification 82

Supplementary Figure 8b: Penicillin Binding Protein 2c (PBP2c) detection....83

Supplementary Figure 8c: Panton-Valentine Leukocidin (PVL) detection....83

References....85

Supplementary Table 1: SRM method for S. aureus multiplexed characterisation

Feature (if not excluded)	Protein name (from UniProt KB)	Accession number (from UniProt KB)	Gene name	Proteotypic peptide sequence	Q1 - Precursor ion (m/z)	Q3 - Fragment ion (m/z)	Target retention time (min)	Collision energy (Volts)	Conserved *versus* excluded transitions (based on train set analysis)[a]
	Alkyl hydroperoxide reductase subunit C	P99074	*ahpC*	NFDVLDEATGLAQR	774.886	960.474	19.40	39.9	excluded
	Alkyl hydroperoxide reductase subunit C	P99074	*ahpC*	NFDVLDEATGLAQR	774.886	1073.559	19.40	39.9	excluded
	Alkyl hydroperoxide reductase subunit C	P99074	*ahp*C	NFDVLDEATGLAQR	774.886	1172.627	19.40	39.9	excluded
I	Bifunctional autolysin	P0C5Z8	*atl*	AYLAVPAAPK	500.790	653.400	14.00	27.0	conserved
I	Bifunctional autolysin	P0C5Z8	*atl*	AYLAVPAAPK	500.790	766.480	14.00	27.0	conserved
I	Bifunctional autolysin	P0C5Z8	*atl*	AYLAVPAAPK	500.790	929.550	14.00	27.0	conserved
	Bifunctional autolysin	P0C5Z8	*atl*	AYLAVPAAPK	504.790	661.400	14.00	27.0	excluded
	Bifunctional autolysin	P0C5Z8	*atl*	AYLAVPAAPK	504.790	774.480	14.00	27.0	excluded
	Bifunctional autolysin	P0C5Z8	*atl*	AYLAVPAAPK	504.790	937.550	14.00	27.0	excluded

	Bifunctional autolysin	P0C5Z8	*atl*	DYNSPTLIGWVK	696.862	913.551	19.60	35.5	conserved
	Bifunctional autolysin	P0C5Z8	*atl*	DYNSPTLIGWVK	696.862	1000.583	19.60	35.5	conserved
	Bifunctional autolysin	P0C5Z8	*atl*	DYNSPTLIGWVK	696.862	1114.625	19.60	35.5	conserved
I	Bifunctional autolysin	P0C5Z8	*atl*	LYSVPWGTYK	607.320	751.380	17.40	32.0	excluded
I	Bifunctional autolysin	P0C5Z8	*atl*	LYSVPWGTYK	607.320	850.450	17.40	32.0	excluded
I	Bifunctional autolysin	P0C5Z8	*atl*	LYSVPWGTYK	607.320	937.480	17.40	32.0	excluded
	Bifunctional autolysin	P0C5Z8	*atl*	LYSVPWGTYK	611.320	759.380	17.40	32.0	excluded
	Bifunctional autolysin	P0C5Z8	*atl*	LYSVPWGTYK	611.320	858.450	17.40	32.0	excluded
	Bifunctional autolysin	P0C5Z8	*atl*	LYSVPWGTYK	611.320	945.480	17.40	32.0	excluded
	Virulence factor EsxA	Q7A7S4	*esxA*	FAQLLEEIK	545.811	744.450	18.30	26.8	excluded
	Virulence factor EsxA	Q7A7S4	*esx*A	FAQLLEEIK	545.811	872.509	18.30	26.8	excluded
	Virulence factor EsxA	Q7A7S4	*esx*A	FAQLLEEIK	545.811	943.546	18.30	26.8	excluded
I	Virulence factor EsxA	Q7A7S4	*esx*A	SQSYGQGSDQIR	663.308	860.422	8.80	33.5	conserved
I	Virulence factor EsxA	Q7A7S4	*esx*A	SQSYGQGSDQIR	663.308	1023.485	8.80	33.5	conserved
I	Virulence factor EsxA	Q7A7S4	*esx*A	SQSYGQGSDQIR	663.308	1110.517	8.80	33.5	conserved
	Glyceraldehyde-3-phosphate dehydrogenase 1	P0A038	*gap*A1	TLAYLAELSK	554.816	823.456	17.80	27.4	excluded

Glyceraldehyde-3-phosphate dehydrogenase 1	P0A038	*gap*A1	TLAYLAELSK	554.816	894.493	17.80	27.4	excluded
Glyceraldehyde-3-phosphate dehydrogenase 1	P0A038	*gap*A1	TLAYLAELSK	554.816	1007.577	17.80	27.4	excluded
Dihydrolipoyl dehydrogenase	P0A0E8	*pdh*D	ALSLDDTNGFVK	640.330	780.389	16.30	32.2	excluded
Dihydrolipoyl dehydrogenase	P0A0E8	*pdh*D	ALSLDDTNGFVK	640.330	895.416	16.30	32.2	excluded
Dihydrolipoyl dehydrogenase	P0A0E8	*pdh*D	ALSLDDTNGFVK	640.330	1008.500	16.30	32.2	excluded
Surface protein	P80544	*pls*	ATTDDLGIDK	524.761	775.383	11.84	25.6	excluded
Surface protein	P80544	*pls*	ATTDDLGIDK	524.761	876.431	11.84	25.6	excluded
Surface protein	P80544	*pls*	ATTDDLGIDK	524.761	977.479	11.84	25.6	excluded
Surface protein	P80544	*pls*	EEIPFEK	446.227	520.277	13.70	21.2	excluded
Surface protein	P80544	*pls*	EEIPFEK	446.227	633.361	13.70	21.2	excluded
Surface protein	P80544	*pls*	EEIPFEK	446.227	762.403	13.70	21.2	excluded
Surface protein	P80544	*pls*	EFNPDLKPGEEK	701.846	800.451	12.03	35.7	excluded
Surface protein	P80544	*pls*	EFNPDLKPGEEK	701.846	915.478	12.03	35.7	excluded

Surface protein	P80544	*pls*	EFNPDLKPGEEK	701.846	1012.531	12.03	35.7	excluded
Surface protein	P80544	*pls*	TQVVDTVAK	480.772	632.361	10.26	23.1	excluded
Surface protein	P80544	*pls*	TQVVDTVAK	480.772	731.430	10.26	23.1	excluded
Surface protein	P80544	*pls*	TQVVDTVAK	480.772	859.488	10.26	23.1	excluded
Surface protein	P80544	*pls*	TTTTPTTINPLTGEK	787.917	871.488	14.82	40.6	excluded
Surface protein	P80544	*pls*	TTTTPTTINPLTGEK	787.917	972.536	14.82	40.6	excluded
Surface protein	P80544	*pls*	TTTTPTTINPLTGEK	787.917	1073.584	14.82	40.6	excluded
Surface protein	P80544	*pls*	VGYGTFVK	435.740	551.319	13.61	20.6	excluded
Surface protein	P80544	*pls*	VGYGTFVK	435.740	714.382	13.61	20.6	excluded
Surface protein	P80544	*pls*	VGYGTFVK	435.740	771.404	13.61	20.6	excluded
50S ribosomal protein L6	Q7A466	*rpl*F	VLELVGVGYR	552.824	763.446	17.40	27.2	excluded
50S ribosomal protein L6	Q7A466	*rpl*F	VLELVGVGYR	552.824	892.489	17.40	27.2	excluded
50S ribosomal protein L6	Q7A466	*rpl*F	VLELVGVGYR	552.824	1005.573	17.40	27.2	excluded
50S ribosomal protein L17	Q7A469	*rpl*Q	DLATSLIISER	609.340	817.480	18.40	32.0	excluded
50S ribosomal protein L17	Q7A469	*rpl*Q	DLATSLIISER	609.340	918.530	18.40	32.0	excluded
50S ribosomal protein L17	Q7A469	*rpl*Q	DLATSLIISER	609.340	989.560	18.40	32.0	excluded
50S ribosomal protein L17	Q7A469	*rpl*Q	DLATSLIISER	614.340	827.480	18.40	32.0	excluded

	50S ribosomal protein L17	Q7A469	*rpl*Q	DLATSLIISER	614.340	928.530	18.40	32.0	excluded
	50S ribosomal protein L17	Q7A469	*rpl*Q	DLATSLIISER	614.340	999.560	18.40	32.0	excluded
I	50S ribosomal protein L20	P66108	*rpl*T	AFAQLVTK	439.261	588.372	13.50	20.8	conserved
I	50S ribosomal protein L20	P66108	*rpl*T	AFAQLVTK	439.261	659.409	13.50	20.8	conserved
I	50S ribosomal protein L20	P66108	*rpl*T	AFAQLVTK	439.261	806.477	13.50	20.8	conserved
	30S ribosomal protein S9	P66646	*rps*I	LVPGEGNITVNNR	691.873	887.469	12.40	35.2	excluded
	30S ribosomal protein S9	P66646	*rps*I	LVPGEGNITVNNR	691.873	1016.512	12.40	35.2	excluded
	30S ribosomal protein S9	P66646	*rps*I	LVPGEGNITVNNR	691.873	1073.533	12.40	35.2	excluded
	30S ribosomal protein S20	Q7A5C0	*rps*T	AAQSNLIHSNK	591.815	825.458	8.00	29.5	excluded
	30S ribosomal protein S20	Q7A5C0	*rps*T	AAQSNLIHSNK	591.815	912.490	8.00	29.5	excluded
	30S ribosomal protein S20	Q7A5C0	*rps*T	AAQSNLIHSNK	591.815	1040.548	8.00	29.5	excluded
I	Staphylococcal secretory antigen ssaA2	Q7A423	*ssa*A2	AGYTVNNTPK	532.770	672.370	8.80	28.0	conserved
I	Staphylococcal secretory antigen ssaA2	Q7A423	*ssa*A2	AGYTVNNTPK	532.770	773.420	8.80	28.0	conserved
I	Staphylococcal secretory antigen ssaA2	Q7A423	*ssa*A2	AGYTVNNTPK	532.770	936.480	8.80	28.0	conserved
	Staphylococcal secretory antigen ssaA2	Q7A423	*ssa*A2	AGYTVNNTPK	536.770	680.370	8.80	28.0	excluded

	Staphylococcal secretory antigen ssaA2	Q7A423	*ssa*A2	AGYTVNNTPK	536.770	781.420	8.80	28.0	excluded
	Staphylococcal secretory antigen ssaA2	Q7A423	*ssa*A2	AGYTVNNTPK	536.770	944.480	8.80	28.0	excluded
I	Staphylococcal secretory antigen ssaA2	Q7A423	*ssa*A2	SISSGYTSGR	507.750	640.300	8.60	27.0	conserved
I	Staphylococcal secretory antigen ssaA2	Q7A423	*ssa*A2	SISSGYTSGR	507.750	727.340	8.60	27.0	conserved
I	Staphylococcal secretory antigen ssaA2	Q7A423	*ssa*A2	SISSGYTSGR	507.750	814.370	8.60	27.0	conserved
	Staphylococcal secretory antigen ssaA2	Q7A423	*ssa*A2	SISSGYTSGR	512.750	650.300	8.60	27.0	excluded
	Staphylococcal secretory antigen ssaA2	Q7A423	*ssa*A2	SISSGYTSGR	512.750	737.340	8.60	27.0	excluded
	Staphylococcal secretory antigen ssaA2	Q7A423	*ssa*A2	SISSGYTSGR	512.750	824.370	8.60	27.0	excluded
I	Triosephosphate isomerase	P68823	*tpi*A	DFVNALPTLPDSK	708.872	870.493	18.60	36.1	conserved
I	Triosephosphate isomerase	P68823	*tpi*A	DFVNALPTLPDSK	708.872	941.530	18.60	36.1	conserved
I	Triosephosphate isomerase	P68823	*tpi*A	DFVNALPTLPDSK	708.872	1055.573	18.60	36.1	conserved

Thioredoxin	P0A0K6	*trx*A	VTDADFDSK	499.227	682.304	9.20	24.2	excluded
Thioredoxin	P0A0K6	*trx*A	VTDADFDSK	499.227	797.331	9.20	24.2	excluded
Thioredoxin	P0A0K6	*trx*A	VTDADFDSK	499.227	898.379	9.20	24.2	excluded
Elongation factor Ts	P99171	*Tsf*	NPDVTVEAFLK	616.830	807.461	18.60	30.9	excluded
Elongation factor Ts	P99171	*tsf*	NPDVTVEAFLK	616.830	906.529	18.60	30.9	excluded
Elongation factor Ts	P99171	*tsf*	NPDVTVEAFLK	616.830	1021.556	18.60	30.9	excluded
Elongation factor Tu	P99152	*tuf*	TVGSGVVTEIIK	601.850	801.510	16.80	31.0	excluded
Elongation factor Tu	P99152	*tuf*	TVGSGVVTEIIK	601.850	858.530	16.80	31.0	excluded
Elongation factor Tu	P99152	*tuf*	TVGSGVVTEIIK	601.850	945.560	16.80	31.0	excluded
Elongation factor Tu	P99152	*tuf*	TVGSGVVTEIIK	605.850	809.510	16.80	31.0	excluded
Elongation factor Tu	P99152	*tuf*	TVGSGVVTEIIK	605.850	866.530	16.80	31.0	excluded
Elongation factor Tu	P99152	*tuf*	TVGSGVVTEIIK	605.850	953.560	16.80	31.0	excluded
UPF0337 protein SA0772	Q7A6L9	SA0772	ADESKFEQAK	576.780	837.446	7.50	28.6	excluded
UPF0337 protein SA0772	Q7A6L9	SA0772	ADESKFEQAK	576.780	966.489	7.50	28.6	excluded
UPF0337 protein SA0772	Q7A6L9	SA0772	ADESKFEQAK	576.780	1081.516	7.50	28.6	excluded
UPF0337 protein SA0772	Q7A6L9	SA0772	AKEFVENAK	518.277	707.372	8.00	25.3	excluded
UPF0337 protein SA0772	Q7A6L9	SA0772	AKEFVENAK	518.277	836.415	8.00	25.3	excluded

UPF0337 protein SA0772	Q7A6L9	SA0772	AKEFVENAK	518.277	964.510	8.00	25.3	excluded
UPF0337 protein SA0772	Q7A6L9	SA0772	ATDFIDKVK	518.790	749.460	12.30	28.0	excluded
UPF0337 protein SA0772	Q7A6L9	SA0772	ATDFIDKVK	518.790	864.480	12.30	28.0	excluded
UPF0337 protein SA0772	Q7A6L9	SA0772	ATDFIDKVK	518.790	965.530	12.30	28.0	excluded
UPF0337 protein SA0772	Q7A6L9	SA0772	ATDFIDKVK	522.790	757.460	12.30	28.0	excluded
UPF0337 protein SA0772	Q7A6L9	SA0772	ATDFIDKVK	522.790	872.480	12.30	28.0	excluded
UPF0337 protein SA0772	Q7A6L9	SA0772	ATDFIDKVK	522.790	973.530	12.30	28.0	excluded
UPF0337 protein SA0772	Q7A6L9	SA0772	EFVENAKEK	547.280	718.370	7.90	29.0	excluded
UPF0337 protein SA0772	Q7A6L9	SA0772	EFVENAKEK	547.280	817.440	7.90	29.0	excluded
UPF0337 protein SA0772	Q7A6L9	SA0772	EFVENAKEK	547.280	964.510	7.90	29.0	excluded
UPF0337 protein SA0772	Q7A6L9	SA0772	EFVENAKEK	551.280	726.370	7.90	29.0	excluded
UPF0337 protein SA0772	Q7A6L9	SA0772	EFVENAKEK	551.280	825.440	7.90	29.0	excluded
UPF0337 protein SA0772	Q7A6L9	SA0772	EFVENAKEK	551.280	972.510	7.90	29.0	excluded
Putative universal stress protein SA1532	Q7A551	SA1532	FIVGSVSESIVR	646.864	876.479	17.40	32.6	excluded
Putative universal stress protein SA1532	Q7A551	SA1532	FIVGSVSESIVR	646.864	933.500	17.40	32.6	excluded
Putative universal stress protein SA1532	Q7A551	SA1532	FIVGSVSESIVR	646.864	1032.568	17.40	32.6	excluded

I	Probable tautomerase SA1195.1	P99132	SAS044	NLVSEVTDAVEK	652.341	890.447	18.90	32.9	conserved
I	Probable tautomerase SA1195.1	P99132	SAS044	NLVSEVTDAVEK	652.341	977.479	18.90	32.9	conserved
I	Probable tautomerase SA1195.1	P99132	SAS044	NLVSEVTDAVEK	652.341	1076.547	18.90	32.9	conserved
	UPF0457 protein SA1975.1	Q99S93	SAS074	LDSKDVSR	460.250	604.340	7.14	25.0	excluded
	UPF0457 protein SA1975.1	Q99S93	SAS074	LDSKDVSR	460.250	691.370	7.14	25.0	excluded
	UPF0457 protein SA1975.1	Q99S93	SAS074	LDSKDVSR	460.250	806.400	7.14	25.0	excluded
	UPF0457 protein SA1975.1	Q99S93	SAS074	LDSKDVSR	465.250	614.340	7.14	25.0	excluded
	UPF0457 protein SA1975.1	Q99S93	SAS074	LDSKDVSR	465.250	701.370	7.14	25.0	excluded
	UPF0457 protein SA1975.1	Q99S93	SAS074	LDSKDVSR	465.250	816.400	7.14	25.0	excluded
I	UPF0457 protein SA1975.1	Q99S93	SAS074	NITQDQDIHAVPK	739.880	664.410	10.90	38.0	conserved
I	UPF0457 protein SA1975.1	Q99S93	SAS074	NITQDQDIHAVPK	739.880	779.440	10.90	38.0	conserved
I	UPF0457 protein SA1975.1	Q99S93	SAS074	NITQDQDIHAVPK	739.880	907.500	10.90	38.0	conserved
	UPF0457 protein SA1975.1	Q99S93	SAS074	NITQDQDIHAVPK	743.880	672.410	10.90	38.0	excluded
	UPF0457 protein SA1975.1	Q99S93	SAS074	NITQDQDIHAVPK	743.880	787.440	10.90	38.0	excluded
	UPF0457 protein SA1975.1	Q99S93	SAS074	NITQDQDIHAVPK	743.880	915.500	10.90	38.0	excluded
R	Penicillin binding protein 2	Q7A8C6	*mec*A[c]	DINIQDR	437.220	418.220	10.30	24.0	conserved

	prime[b]								
R	Penicillin binding protein 2 prime[b]	Q7A8C6	*mec*A[c]	DINIQDR	437.220	531.300	10.30	24.0	conserved
R	Penicillin binding protein 2 prime[b]	Q7A8C6	*mec*A[c]	DINIQDR	437.220	645.330	10.30	24.0	conserved
	Penicillin binding protein 2 prime[b]	Q7A8C6	*mec*A[c]	DINIQDR	442.220	428.220	10.30	24.0	excluded
	Penicillin binding protein 2 prime[b]	Q7A8C6	*mec*A[c]	DINIQDR	442.220	541.300	10.30	24.0	excluded
	Penicillin binding protein 2 prime[b]	Q7A8C6	*mec*A[c]	DINIQDR	442.220	655.330	10.30	24.0	excluded
R	Penicillin binding protein 2 prime[b]	Q7A8C6	*mec*A[c]	IYNSLGVK	447.260	503.300	11.70	25.0	conserved
R	Penicillin binding protein 2 prime[b]	Q7A8C6	*mec*A[c]	IYNSLGVK	447.260	617.360	11.70	25.0	conserved
R	Penicillin binding protein 2 prime[b]	Q7A8C6	*mec*A[c]	IYNSLGVK	447.260	780.430	11.70	25.0	conserved
	Penicillin binding protein 2 prime[b]	Q7A8C6	*mec*A[c]	IYNSLGVK	451.260	511.300	11.70	25.0	excluded
	Penicillin binding protein 2 prime[b]	Q7A8C6	*mec*A[c]	IYNSLGVK	451.260	625.360	11.70	25.0	excluded

	Penicillin binding protein 2 prime[b]	Q7A8C6	*mec*A[c]	IYNSLGVK	451.260	788.430	11.70	25.0	excluded
R	Penicillin binding protein 2 prime[b]	Q7A8C6	*mec*A[c]	ILTAMIGLNNK	594.340	789.430	17.30	31.0	conserved
R	Penicillin binding protein 2 prime[b]	Q7A8C6	*mec*A[c]	ILTAMIGLNNK	594.340	860.470	17.30	31.0	conserved
R	Penicillin binding protein 2 prime[b]	Q7A8C6	*mec*A[c]	ILTAMIGLNNK	594.340	961.510	17.30	31.0	conserved
	Penicillin binding protein 2 prime[b]	Q7A8C6	*mec*A[c]	ILTAMIGLNNK	598.340	797.430	17.30	31.0	excluded
	Penicillin binding protein 2 prime[b]	Q7A8C6	*mec*A[c]	ILTAMIGLNNK	598.340	868.470	17.30	31.0	excluded
	Penicillin binding protein 2 prime[b]	Q7A8C6	*mec*A[c]	ILTAMIGLNNK	598.340	969.510	17.30	31.0	excluded
R	Penicillin binding protein 2 prime[b]	Q7A8C6	*mec*A[c]	ELSISEDYIK	598.810	754.360	15.80	31.0	conserved
R	Penicillin binding protein 2 prime[b]	Q7A8C6	*mec*A[c]	ELSISEDYIK	598.810	867.450	15.80	31.0	conserved
R	Penicillin binding protein 2 prime[b]	Q7A8C6	*mec*A[c]	ELSISEDYIK	598.810	954.480	15.80	31.0	conserved
	Penicillin binding protein 2 prime[b]	Q7A8C6	*mec*A[c]	ELSISEDYIK	602.810	762.360	15.80	31.0	excluded

	Penicillin binding protein 2 prime[b]	Q7A8C6	*mec*A[c]	ELSISEDYIK	602.810	875.450	15.80	31.0	excluded
	Penicillin binding protein 2 prime[b]	Q7A8C6	*mec*A[c]	ELSISEDYIK	602.810	962.480	15.80	31.0	excluded
R	Penicillin binding protein 2 prime[b]	Q7A8C6	*mec*A[c]	FQITTSPGSTQK	647.840	617.300	12.20	26.0	conserved[e]
R	Penicillin binding protein 2 prime[b]	Q7A8C6	*mec*A[c]	FQITTSPGSTQK	647.840	704.400	12.20	34.0	conserved[e]
R	Penicillin binding protein 2 prime[b]	Q7A8C6	*mec*A[c]	FQITTSPGSTQK	647.840	906.450	12.20	34.0	conserved[e]
	Penicillin binding protein 2 prime[b]	Q7A8C6	*mec*A[c]	FQITTSPGSTQK	651.840	625.300	12.20	26.0	excluded
	Penicillin binding protein 2 prime[b]	Q7A8C6	*mec*A[c]	FQITTSPGSTQK	651.840	712.400	12.20	34.0	excluded
	Penicillin binding protein 2 prime[b]	Q7A8C6	*mec*A[c]	FQITTSPGSTQK	651.840	914.450	12.20	34.0	excluded
R	Penicillin binding protein 2 prime[b]	Q7A8C6	*mec*A[c]	SYANLIGK	433.240	544.300	13.00	24.0	conserved
	Penicillin binding protein 2 prime[b]	Q7A8C6	*mec*A[c]	SYANLIGK	437.240	552.300	13.00	24.0	excluded
R	Penicillin binding protein 2 prime[b]	Q7A8C6	*mec*A[c]	SYANLIGK	433.240	615.380	13.00	24.0	conserved

	Penicillin binding protein 2 prime[b]	Q7A8C6	*mec*A[c]	SYANLIGK	437.240	623.380	13.00	24.0	excluded
R	Penicillin binding protein 2 prime[b]	Q7A8C6	*mec*A[c]	SYANLIGK	433.240	778.450	13.00	24.0	conserved
	Penicillin binding protein 2 prime[b]	Q7A8C6	*mec*A[c]	SYANLIGK	437.240	786.450	13.00	24.0	excluded
R	Penicillin binding protein 2 prime[b]	Q7A8C6	*mec*A[c]	VALELGSK	408.740	533.290	12.70	23.0	conserved
	Penicillin binding protein 2 prime[b]	Q7A8C6	*mec*A[c]	VALELGSK	412.740	541.290	12.70	23.0	excluded
R	Penicillin binding protein 2 prime[b]	Q7A8C6	*mec*A[c]	VALELGSK	408.740	646.380	12.70	23.0	conserved
	Penicillin binding protein 2 prime[b]	Q7A8C6	*mec*A[c]	VALELGSK	412.740	654.380	12.70	23.0	excluded
R	Penicillin binding protein 2 prime[b]	Q7A8C6	*mec*A[c]	VALELGSK	408.740	717.410	12.70	23.0	conserved
	Penicillin binding protein 2 prime[b]	Q7A8C6	*mec*A[c]	VALELGSK	412.740	725.410	12.70	23.0	excluded
R	Penicillin binding protein 2 prime[b]	Q7A8C6	*mec*A[c]	YEVVNGNIDLK	632.330	773.420	14.65	33.0	conserved
	Penicillin binding protein 2 prime[b]	Q7A8C6	*mec*A[c]	YEVVNGNIDLK	636.330	781.420	14.65	33.0	excluded

R	Penicillin binding protein 2 prime[b]	Q7A8C6	*mec*A[c]	YEVVNGNIDLK	632.330	872.480	14.65	33.0	conserved
	Penicillin binding protein 2 prime[b]	Q7A8C6	*mec*A[c]	YEVVNGNIDLK	636.330	880.480	14.65	33.0	excluded
R	Penicillin binding protein 2 prime[b]	Q7A8C6	*mec*A[c]	YEVVNGNIDLK	632.330	971.550	14.65	33.0	conserved
	Penicillin binding protein 2 prime[b]	Q7A8C6	*mec*A[c]	YEVVNGNIDLK	636.330	979.550	14.65	33.0	excluded
R	Beta-lactam-inducible penicillin-binding protein[d]	G0LPN8	*mec*C[c]	FQITTSPGSTQK	647.840	617.330	12.30	26.0	conserved[e]
R	Beta-lactam-inducible penicillin-binding protein[d]	G0LPN8	*mec*C[c]	FQITTSPGSTQK	647.840	805.410	12.30	34.0	conserved[e]
R	Beta-lactam-inducible penicillin-binding protein[d]	G0LPN8	*mec*C[c]	FQITTSPGSTQK	647.840	906.450	12.30	34.0	conserved[e]
R	Beta-lactam-inducible penicillin-binding protein[d]	G0LPN8	*mec*C[c]	IALALGAK	378.750	459.290	14.10	22.0	conserved
R	Beta-lactam-inducible penicillin-binding protein[d]	G0LPN8	*mec*C[c]	IALALGAK	378.750	572.380	14.10	22.0	conserved
R	Beta-lactam-inducible penicillin-binding protein[d]	G0LPN8	*mec*C[c]	IALALGAK	378.750	643.410	14.10	22.0	conserved
R	Beta-lactam-inducible penicillin-binding protein[d]	G0LPN8	*mec*C[c]	ILTSIIALK	486.330	644.430	18.80	26.0	conserved

R	Beta-lactam-inducible penicillin-binding protein[d]	G0LPN8	*mec*C[c]	ILTSIIALK	486.330	745.480	18.80	26.0	conserved
R	Beta-lactam-inducible penicillin-binding protein[d]	G0LPN8	*mec*C[c]	ILTSIIALK	486.330	858.570	18.80	26.0	conserved
	Beta-lactam-inducible penicillin-binding protein[d]	G0LPN8	*mec*C[c]	INIETLK	415.750	490.290	13.65	23.0	excluded
	Beta-lactam-inducible penicillin-binding protein[d]	G0LPN8	*mec*C[c]	INIETLK	415.750	603.370	13.65	23.0	excluded
	Beta-lactam-inducible penicillin-binding protein[d]	G0LPN8	*mec*C[c]	INIETLK	415.750	717.410	13.65	23.0	excluded
R	Beta-lactam-inducible penicillin-binding protein[d]	G0LPN8	*mec*C[c]	LAYGEEEIVDR	647.320	760.380	13.50	33.0	conserved
R	Beta-lactam-inducible penicillin-binding protein[d]	G0LPN8	*mec*C[c]	LAYGEEEIVDR	647.320	946.450	13.50	33.0	conserved
R	Beta-lactam-inducible penicillin-binding protein[d]	G0LPN8	*mec*C[c]	LAYGEEEIVDR	647.320	1109.510	13.50	33.0	conserved
	Beta-lactam-inducible penicillin-binding protein[d]	G0LPN8	*mec*C[c]	NNPNMLMAINVK	679.850	565.810	17.97	35.0	excluded
	Beta-lactam-inducible penicillin-binding protein[d]	G0LPN8	*mec*C[c]	NNPNMLMAINVK	679.850	919.510	17.97	35.0	excluded
	Beta-lactam-inducible penicillin-binding protein[d]	G0LPN8	*mec*C[c]	NNPNMLMAINVK	679.850	1130.610	17.97	35.0	excluded

	Beta-lactam-inducible penicillin-binding protein[d]	G0LPN8	*mec*C[c]	NTNFDIYGK	536.260	742.380	13.99	29.0	excluded
	Beta-lactam-inducible penicillin-binding protein[d]	G0LPN8	*mec*C[c]	NTNFDIYGK	536.260	856.420	13.99	29.0	excluded
	Beta-lactam-inducible penicillin-binding protein[d]	G0LPN8	*mec*C[c]	NTNFDIYGK	536.260	957.470	13.99	29.0	excluded
	Beta-lactam-inducible penicillin-binding protein[d]	G0LPN8	*mec*C[c]	NTQLNFIYEDK	692.840	667.330	16.61	35.0	excluded
	Beta-lactam-inducible penicillin-binding protein[d]	G0LPN8	*mec*C[c]	NTQLNFIYEDK	692.840	814.400	16.61	35.0	excluded
	Beta-lactam-inducible penicillin-binding protein[d]	G0LPN8	*mec*C[c]	NTQLNFIYEDK	692.840	928.440	16.61	35.0	excluded
	Beta-lactam-inducible penicillin-binding protein[d]	G0LPN8	*mec*C[c]	QAIESSDNIFFAR	749.370	540.290	17.60	38.0	excluded
	Beta-lactam-inducible penicillin-binding protein[d]	G0LPN8	*mec*C[c]	QAIESSDNIFFAR	749.370	767.420	17.60	38.0	excluded
	Beta-lactam-inducible penicillin-binding protein[d]	G0LPN8	*mec*C[c]	QAIESSDNIFFAR	749.370	882.450	17.60	38.0	excluded
R	Beta-lactam-inducible penicillin-binding protein[d]	G0LPN8	*mec*C[c]	SYNLQINTIK	597.330	716.430	15.10	31.0	conserved
R	Beta-lactam-inducible penicillin-binding protein[d]	G0LPN8	*mec*C[c]	SYNLQINTIK	597.330	829.510	15.10	31.0	conserved

R	Beta-lactam-inducible penicillin-binding protein[d]	G0LPN8	*mec*C[c]	SYNLQINTIK	597.330	943.560	15.10	31.0	conserved
	Beta-lactam-inducible penicillin-binding protein[d]	G0LPN8	*mec*C[c]	TGNTYEIGIVPNK	703.370	358.210	14.70	36.0	excluded
	Beta-lactam-inducible penicillin-binding protein[d]	G0LPN8	*mec*C[c]	TGNTYEIGIVPNK	703.370	740.470	14.70	36.0	excluded
	Beta-lactam-inducible penicillin-binding protein[d]	G0LPN8	*mec*C[c]	TGNTYEIGIVPNK	703.370	869.510	14.70	36.0	excluded
	Beta-lactam-inducible penicillin-binding protein[d]	G0LPN8	*mec*C[c]	VYDDLYDNGK	601.270	824.380	11.65	31.0	excluded
	Beta-lactam-inducible penicillin-binding protein[d]	G0LPN8	*mec*C[c]	VYDDLYDNGK	601.270	939.410	11.65	31.0	excluded
	Beta-lactam-inducible penicillin-binding protein[d]	G0LPN8	*mec*C[c]	VYDDLYDNGK	601.270	1102.470	11.65	31.0	excluded
	Immunoglobulin G-binding protein A	P38507	*spa*	ADAQQNK	387.690	517.270	1.38	17.0	excluded
	Immunoglobulin G-binding protein A	P38507	*spa*	ADAQQNK	387.690	588.310	1.38	18.0	excluded
	Immunoglobulin G-binding protein A	P38507	*spa*	ADAQQNK	387.690	703.340	1.38	18.0	excluded
	Immunoglobulin G-binding protein A	P38507	*spa*	ADAQQNK	391.690	525.270	1.38	17.0	excluded

Immunoglobulin G-binding protein A	P38507	*spa*	ADAQQNK	391.690	596.310	1.38	18.0	excluded
Immunoglobulin G-binding protein A	P38507	*spa*	ADAQQNK	391.690	711.340	1.38	18.0	excluded
Immunoglobulin G-binding protein A	P38507	*spa*	ADNNFNK	411.690	522.270	5.68	20.0	excluded
Immunoglobulin G-binding protein A	P38507	*spa*	ADNNFNK	411.690	636.310	5.68	18.0	excluded
Immunoglobulin G-binding protein A	P38507	*spa*	ADNNFNK	411.690	751.340	5.68	18.0	excluded
Immunoglobulin G-binding protein A	P38507	*spa*	ADNNFNK	415.690	530.270	5.68	20.0	excluded
Immunoglobulin G-binding protein A	P38507	*spa*	ADNNFNK	415.690	644.310	5.68	18.0	excluded
Immunoglobulin G-binding protein A	P38507	*spa*	ADNNFNK	415.690	759.340	5.68	18.0	excluded
Immunoglobulin G-binding protein A	P38507	*spa*	ANGTTADK	389.190	434.220	1.38	20.0	excluded
Immunoglobulin G-binding protein A	P38507	*spa*	ANGTTADK	389.190	592.290	1.38	18.0	excluded
Immunoglobulin G-binding protein A	P38507	*spa*	ANGTTADK	389.190	706.340	1.38	18.0	excluded

	Immunoglobulin G-binding protein A	P38507	*spa*	ANGTTADK	393.190	442.220	1.38	20.0	excluded
	Immunoglobulin G-binding protein A	P38507	*spa*	ANGTTADK	393.190	600.290	1.38	18.0	excluded
	Immunoglobulin G-binding protein A	P38507	*spa*	ANGTTADK	393.190	714.340	1.38	18.0	excluded
T	Immunoglobulin G-binding protein A	P38507	*spa*	DDPSQSANLLAEAK	729.860	614.830	14.00	32.0	conserved
T	Immunoglobulin G-binding protein A	P38507	*spa*	DDPSQSANLLAEAK	729.860	829.480	14.00	35.0	conserved
T	Immunoglobulin G-binding protein A	P38507	*spa*	DDPSQSANLLAEAK	729.860	916.510	14.00	35.0	conserved
	Immunoglobulin G-binding protein A	P38507	*spa*	DDPSQSANLLAEAK	733.860	618.830	14.00	32.0	excluded
	Immunoglobulin G-binding protein A	P38507	*spa*	DDPSQSANLLAEAK	733.860	837.480	14.00	35.0	excluded
	Immunoglobulin G-binding protein A	P38507	*spa*	DDPSQSANLLAEAK	733.860	924.510	14.00	35.0	excluded
T	Immunoglobulin G-binding protein A	P38507	*spa*	DDPSQSANVLGEAQK	779.870	532.270	11.90	34.0	conserved
T	Immunoglobulin G-binding protein A	P38507	*spa*	DDPSQSANVLGEAQK	779.870	664.840	11.90	34.0	conserved

T	Immunoglobulin G-binding protein A	P38507	*spa*	DDPSQSANVLGEAQK	779.870	929.510	11.90	35.0	conserved
	Immunoglobulin G-binding protein A	P38507	*spa*	DDPSQSANVLGEAQK	783.870	540.270	11.90	34.0	excluded
	Immunoglobulin G-binding protein A	P38507	*spa*	DDPSQSANVLGEAQK	783.870	668.870	11.90	34.0	excluded
	Immunoglobulin G-binding protein A	P38507	*spa*	DDPSQSANVLGEAQK	783.870	937.510	11.90	35.0	excluded
T	Immunoglobulin G-binding protein A	P38507	*spa*	DDPSQSTNVLGEAK	730.850	615.820	11.70	33.0	conserved
T	Immunoglobulin G-binding protein A	P38507	*spa*	DDPSQSTNVLGEAK	730.850	831.460	11.70	40.0	conserved
T	Immunoglobulin G-binding protein A	P38507	*spa*	DDPSQSTNVLGEAK	730.850	918.490	11.70	38.0	conserved
	Immunoglobulin G-binding protein A	P38507	*spa*	DDPSQSTNVLGEAK	734.850	619.820	11.70	33.0	excluded
	Immunoglobulin G-binding protein A	P38507	*spa*	DDPSQSTNVLGEAK	734.850	839.460	11.70	40.0	excluded
	Immunoglobulin G-binding protein A	P38507	*spa*	DDPSQSTNVLGEAK	734.850	926.490	11.70	38.0	excluded
	Immunoglobulin G-binding protein A	P38507	*spa*	DDPSVSK	374.180	259.150	5.39	18.0	excluded

	Immunoglobulin G-binding protein A	P38507	*spa*	DDPSVSK	374.180	420.250	5.39	24.0	excluded
	Immunoglobulin G-binding protein A	P38507	*spa*	DDPSVSK	374.180	517.300	5.39	16.0	excluded
	Immunoglobulin G-binding protein A	P38507	*spa*	DDPSVSK	378.180	263.150	5.39	18.0	excluded
	Immunoglobulin G-binding protein A	P38507	*spa*	DDPSVSK	378.180	428.250	5.39	24.0	excluded
	Immunoglobulin G-binding protein A	P38507	*spa*	DDPSVSK	378.180	525.300	5.39	16.0	excluded
T	Immunoglobulin G-binding protein A	P38507	*spa*	DQQSAFYEILNMPNLNEEQR	813.710	500.250	22.50	28.0	conserved
T	Immunoglobulin G-binding protein A	P38507	*spa*	DQQSAFYEILNMPNLNEEQR	813.710	969.390	22.50	32.0	conserved
T	Immunoglobulin G-binding protein A	P38507	*spa*	DQQSAFYEILNMPNLNEEQR	813.710	999.490	22.50	33.0	conserved
	Immunoglobulin G-binding protein A	P38507	*spa*	DQQSAFYEILNMPNLNEEQR	817.040	505.250	22.50	28.0	excluded
	Immunoglobulin G-binding protein A	P38507	*spa*	DQQSAFYEILNMPNLNEEQR	817.040	969.390	22.50	32.0	excluded
	Immunoglobulin G-binding protein A	P38507	*spa*	DQQSAFYEILNMPNLNEEQR	817.040	1009.490	22.50	33.0	excluded

T	Immunoglobulin G-binding protein A	P38507	*spa*	EDGNGVHVVKPGDTVNDIAK	688.680	515.260	12.40	34.0	conserved
T	Immunoglobulin G-binding protein A	P38507	*spa*	EDGNGVHVVKPGDTVNDIAK	688.680	746.910	12.40	33.0	conserved
T	Immunoglobulin G-binding protein A	P38507	*spa*	EDGNGVHVVKPGDTVNDIAK	688.680	1029.520	12.40	36.0	conserved
	Immunoglobulin G-binding protein A	P38507	*spa*	EDGNGVHVVKPGDTVNDIAK	691.350	519.260	12.40	34.0	excluded
	Immunoglobulin G-binding protein A	P38507	*spa*	EDGNGVHVVKPGDTVNDIAK	691.350	750.910	12.40	33.0	excluded
	Immunoglobulin G-binding protein A	P38507	*spa*	EDGNGVHVVKPGDTVNDIAK	691.350	1037.520	12.40	36.0	excluded
	Immunoglobulin G-binding protein A	P38507	*spa*	EDGNKPGK	422.710	301.190	1.38	27.0	excluded
	Immunoglobulin G-binding protein A	P38507	*spa*	EDGNKPGK	422.710	429.280	1.38	26.0	excluded
	Immunoglobulin G-binding protein A	P38507	*spa*	EDGNKPGK	422.710	600.350	1.38	24.0	excluded
	Immunoglobulin G-binding protein A	P38507	*spa*	EDGNKPGK	426.710	309.190	1.38	27.0	excluded
	Immunoglobulin G-binding protein A	P38507	*spa*	EDGNKPGK	426.710	437.280	1.38	26.0	excluded

	Immunoglobulin G-binding protein A	P38507	*spa*	EDGNKPGK	426.710	608.350	1.38	24.0	excluded
	Immunoglobulin G-binding protein A	P38507	*spa*	EEDNNKPGK	515.740	301.190	1.37	31.0	excluded
	Immunoglobulin G-binding protein A	P38507	*spa*	EEDNNKPGK	515.740	429.280	1.37	31.0	excluded
	Immunoglobulin G-binding protein A	P38507	*spa*	EEDNNKPGK	515.740	772.400	1.37	27.0	excluded
	Immunoglobulin G-binding protein A	P38507	*spa*	EEDNNKPGK	519.740	309.190	1.37	31.0	excluded
	Immunoglobulin G-binding protein A	P38507	*spa*	EEDNNKPGK	519.740	437.280	1.37	31.0	excluded
	Immunoglobulin G-binding protein A	P38507	*spa*	EEDNNKPGK	519.740	780.400	1.37	27.0	excluded
T	Immunoglobulin G-binding protein A	P38507	*spa*	EILAEAK	387.220	347.190	10.00	15.0	conserved
T	Immunoglobulin G-binding protein A	P38507	*spa*	EILAEAK	387.220	418.230	10.00	18.0	conserved
T	Immunoglobulin G-binding protein A	P38507	*spa*	EILAEAK	387.220	531.310	10.00	19.0	conserved
	Immunoglobulin G-binding protein A	P38507	*spa*	EILAEAK	391.220	355.190	10.00	15.0	excluded

	Immunoglobulin G-binding protein A	P38507	*spa*	EILAEAK	391.220	426.230	10.00	18.0	excluded
	Immunoglobulin G-binding protein A	P38507	*spa*	EILAEAK	391.220	539.310	10.00	19.0	excluded
T	Immunoglobulin G-binding protein A	P38507	*spa*	EQQNAFYEILHLPNLNEEQR	829.080	884.450	22.55	33.0	conserved
T	Immunoglobulin G-binding protein A	P38507	*spa*	EQQNAFYEILHLPNLNEEQR	829.080	962.980	22.55	33.0	conserved
T	Immunoglobulin G-binding protein A	P38507	*spa*	EQQNAFYEILHLPNLNEEQR	829.080	993.510	22.55	33.0	conserved
	Immunoglobulin G-binding protein A	P38507	*spa*	EQQNAFYEILHLPNLNEEQR	832.400	889.450	22.55	33.0	excluded
	Immunoglobulin G-binding protein A	P38507	*spa*	EQQNAFYEILHLPNLNEEQR	832.400	962.980	22.55	33.0	excluded
	Immunoglobulin G-binding protein A	P38507	*spa*	EQQNAFYEILHLPNLNEEQR	832.400	998.510	22.55	33.0	excluded
T	Immunoglobulin G-binding protein A	P38507	*spa*	EQQNAFYEILHLPNLTEEQR	824.740	877.960	23.20	33.0	conserved
T	Immunoglobulin G-binding protein A	P38507	*spa*	EQQNAFYEILHLPNLTEEQR	824.740	951.490	23.20	33.0	conserved
T	Immunoglobulin G-binding protein A	P38507	*spa*	EQQNAFYEILHLPNLTEEQR	824.740	986.490	23.20	44.0	conserved

	Immunoglobulin G-binding protein A	P38507	*spa*	EQQNAFYEILHLPNLTEEQR	828.070	882.960	23.20	33.0	excluded
	Immunoglobulin G-binding protein A	P38507	*spa*	EQQNAFYEILHLPNLTEEQR	828.070	956.490	23.20	33.0	excluded
	Immunoglobulin G-binding protein A	P38507	*spa*	EQQNAFYEILHLPNLTEEQR	828.070	996.490	23.20	44.0	excluded
T	Immunoglobulin G-binding protein A	P38507	*spa*	EQQNAFYEILNMPNLNEEQR	827.390	500.250	22.50	30.0	conserved
T	Immunoglobulin G-binding protein A	P38507	*spa*	EQQNAFYEILNMPNLNEEQR	827.390	735.870	22.50	24.0	conserved
T	Immunoglobulin G-binding protein A	P38507	*spa*	EQQNAFYEILNMPNLNEEQR	827.390	999.490	22.50	31.0	conserved
	Immunoglobulin G-binding protein A	P38507	*spa*	EQQNAFYEILNMPNLNEEQR	830.720	505.250	22.50	30.0	excluded
	Immunoglobulin G-binding protein A	P38507	*spa*	EQQNAFYEILNMPNLNEEQR	830.720	740.870	22.50	24.0	excluded
	Immunoglobulin G-binding protein A	P38507	*spa*	EQQNAFYEILNMPNLNEEQR	830.720	1009.490	22.50	31.0	excluded
	Immunoglobulin G-binding protein A	P38507	*spa*	IAADNK	316.170	376.180	2.18	22.0	excluded
	Immunoglobulin G-binding protein A	P38507	*spa*	IAADNK	316.170	447.220	2.18	14.0	excluded

	Immunoglobulin G-binding protein A	P38507	*spa*	IAADNK	316.170	518.260	2.18	15.0	excluded
	Immunoglobulin G-binding protein A	P38507	*spa*	IAADNK	320.170	384.180	2.18	22.0	excluded
	Immunoglobulin G-binding protein A	P38507	*spa*	IAADNK	320.170	455.220	2.18	14.0	excluded
	Immunoglobulin G-binding protein A	P38507	*spa*	IAADNK	320.170	526.260	2.18	15.0	excluded
T	Immunoglobulin G-binding protein A	P38507	*spa*	LNDAQAPK	428.730	514.300	7.00	23.0	conserved
T	Immunoglobulin G-binding protein A	P38507	*spa*	LNDAQAPK	428.730	629.330	7.00	21.0	conserved
T	Immunoglobulin G-binding protein A	P38507	*spa*	LNDAQAPK	428.730	743.370	7.00	20.0	conserved
	Immunoglobulin G-binding protein A	P38507	*spa*	LNDAQAPK	432.760	522.300	7.00	23.0	excluded
	Immunoglobulin G-binding protein A	P38507	*spa*	LNDAQAPK	432.760	637.330	7.00	21.0	excluded
	Immunoglobulin G-binding protein A	P38507	*spa*	LNDAQAPK	432.760	751.370	7.00	20.0	excluded
T	Immunoglobulin G-binding protein A	P38507	*spa*	LNDSQAPK	436.730	530.290	6.80	23.0	conserved

T	Immunoglobulin G-binding protein A	P38507	*spa*	LNDSQAPK	436.730	645.320	6.80	19.0	conserved
T	Immunoglobulin G-binding protein A	P38507	*spa*	LNDSQAPK	436.730	759.360	6.80	20.0	conserved
	Immunoglobulin G-binding protein A	P38507	*spa*	LNDSQAPK	440.730	538.290	6.80	23.0	excluded
	Immunoglobulin G-binding protein A	P38507	*spa*	LNDSQAPK	440.730	653.320	6.80	19.0	excluded
	Immunoglobulin G-binding protein A	P38507	*spa*	LNDSQAPK	440.730	767.360	6.80	20.0	excluded
T	Immunoglobulin G-binding protein A	P38507	*spa*	LNESQAPK	443.740	530.290	7.10	23.0	conserved
T	Immunoglobulin G-binding protein A	P38507	*spa*	LNESQAPK	443.740	659.340	7.10	21.0	conserved
T	Immunoglobulin G-binding protein A	P38507	*spa*	LNESQAPK	443.740	773.380	7.10	22.0	conserved
	Immunoglobulin G-binding protein A	P38507	*spa*	LNESQAPK	447.740	538.290	7.10	23.0	excluded
	Immunoglobulin G-binding protein A	P38507	*spa*	LNESQAPK	447.740	667.340	7.10	21.0	excluded
	Immunoglobulin G-binding protein A	P38507	*spa*	LNESQAPK	447.740	781.380	7.10	22.0	excluded

T	Immunoglobulin G-binding protein A	P38507	*spa*	NGFIQSLK	453.760	475.290	14.40	23.0	conserved
T	Immunoglobulin G-binding protein A	P38507	*spa*	NGFIQSLK	453.760	588.370	14.40	23.0	conserved
T	Immunoglobulin G-binding protein A	P38507	*spa*	NGFIQSLK	453.760	792.460	14.40	23.0	conserved
	Immunoglobulin G-binding protein A	P38507	*spa*	NGFIQSLK	457.760	483.290	14.40	23.0	excluded
	Immunoglobulin G-binding protein A	P38507	*spa*	NGFIQSLK	457.760	596.370	14.40	23.0	excluded
	Immunoglobulin G-binding protein A	P38507	*spa*	NGFIQSLK	457.760	800.460	14.40	23.0	excluded
T	Immunoglobulin G-binding protein A	P38507	*spa*	NMIKPGQELVVDK	735.900	556.820	13.40	38.0	conserved
T	Immunoglobulin G-binding protein A	P38507	*spa*	NMIKPGQELVVDK	735.900	830.460	13.40	41.0	conserved
T	Immunoglobulin G-binding protein A	P38507	*spa*	NMIKPGQELVVDK	735.900	984.540	13.40	38.0	conserved
	Immunoglobulin G-binding protein A	P38507	*spa*	NMIKPGQELVVDK	739.900	560.820	13.40	38.0	excluded
	Immunoglobulin G-binding protein A	P38507	*spa*	NMIKPGQELVVDK	739.900	838.460	13.40	41.0	excluded

Immunoglobulin G-binding protein A	P38507	*spa*	NMIKPGQELVVDK	739.900	992.540	13.40	38.0	excluded
Immunoglobulin G-binding protein A	P38507	*spa*	QPANHADANK	533.260	332.190	9.31	29.0	excluded
Immunoglobulin G-binding protein A	P38507	*spa*	QPANHADANK	533.260	447.220	9.31	29.0	excluded
Immunoglobulin G-binding protein A	P38507	*spa*	QPANHADANK	533.260	840.400	9.31	28.0	excluded
Immunoglobulin G-binding protein A	P38507	*spa*	QPANHADANK	537.260	340.190	9.31	29.0	excluded
Immunoglobulin G-binding protein A	P38507	*spa*	QPANHADANK	537.260	455.220	9.31	29.0	excluded
Immunoglobulin G-binding protein A	P38507	*spa*	QPANHADANK	537.260	848.400	9.31	28.0	excluded
Delta-hemolysin	P0C1V1	*hld*	AQDIISTIGDLVK	686.890	745.450	22.80	35.0	excluded
Delta-hemolysin	P0C1V1	*hld*	AQDIISTIGDLVK	686.890	832.480	22.80	35.0	excluded
Delta-hemolysin	P0C1V1	*hld*	AQDIISTIGDLVK	686.890	945.560	22.80	35.0	excluded
Delta-hemolysin	P0C1V1	*hld*	AQDIISTISDLVK	701.890	775.460	23.47	36.0	excluded
Delta-hemolysin	P0C1V1	*hld*	AQDIISTISDLVK	701.890	862.490	23.47	36.0	excluded
Delta-hemolysin	P0C1V1	*hld*	AQDIISTISDLVK	701.890	975.570	23.47	36.0	excluded

Delta-hemolysin	P0C1V1	*hld*	M[For]AQDIISTIGDLVK	766.410	474.170	28.44	38.0	excluded
Delta-hemolysin	P0C1V1	*hld*	M[For]AQDIISTIGDLVK	766.410	832.480	28.44	38.0	excluded
Delta-hemolysin	P0C1V1	*hld*	M[For]AQDIISTIGDLVK	766.410	945.560	28.44	38.0	excluded
Delta-hemolysin	P0C1V1	*hld*	M[For]AQDIISTISDLVK	781.410	474.170	28.68	38.0	excluded
Delta-hemolysin	P0C1V1	*hld*	M[For]AQDIISTISDLVK	781.410	862.490	28.68	38.0	excluded
Delta-hemolysin	P0C1V1	*hld*	M[For]AQDIISTISDLVK	781.410	975.570	28.68	38.0	excluded
Delta-hemolysin	P0C1V1	*hld*	M[Oxi]AQDIISTIGDLVK	760.410	462.170	22.80	38.0	excluded
Delta-hemolysin	P0C1V1	*hld*	M[Oxi]AQDIISTIGDLVK	760.410	832.480	22.80	38.0	excluded
Delta-hemolysin	P0C1V1	*hld*	M[Oxi]AQDIISTIGDLVK	760.410	945.560	22.80	38.0	excluded
Delta-hemolysin	P0C1V1	*hld*	M[Oxi]AQDIISTISDLVK	775.410	674.410	23.56	39.0	excluded
Delta-hemolysin	P0C1V1	*hld*	M[Oxi]AQDIISTISDLVK	775.410	862.490	23.56	39.0	excluded
Delta-hemolysin	P0C1V1	*hld*	M[Oxi]AQDIISTISDLVK	775.410	975.570	23.56	39.0	excluded
Delta-hemolysin	P0C1V1	*hld*	MAQDIISTIGDLVK	752.410	446.170	23.49	38.0	excluded
Delta-hemolysin	P0C1V1	*hld*	MAQDIISTIGDLVK	752.410	832.480	23.49	38.0	excluded
Delta-hemolysin	P0C1V1	*hld*	MAQDIISTIGDLVK	752.410	945.560	23.49	38.0	excluded
Delta-hemolysin	P0C1V1	*hld*	MAQDIISTISDLVK	767.410	674.410	24.21	39.0	excluded
Delta-hemolysin	P0C1V1	*hld*	MAQDIISTISDLVK	767.410	775.460	24.21	39.0	excluded

	Delta-hemolysin	P0C1V1	*hld*	MAQDIISTISDLVK	767.410	862.490	24.21	39.0	excluded
	Delta-hemolysin	P0C1V1	*hld*	WIIDTVNK	494.780	576.300	16.35	27.0	excluded
	Delta-hemolysin	P0C1V1	*hld*	WIIDTVNK	494.780	689.380	16.35	27.0	excluded
	Delta-hemolysin	P0C1V1	*hld*	WIIDTVNK	494.780	802.470	16.35	27.0	excluded
	Delta-hemolysin	P0C1V1	*hld*	WIIDTVNKFTK	682.880	736.440	18.21	35.0	excluded
	Delta-hemolysin	P0C1V1	*hld*	WIIDTVNKFTK	682.880	837.480	18.21	35.0	excluded
	Delta-hemolysin	P0C1V1	*hld*	WIIDTVNKFTK	682.880	952.510	18.21	35.0	excluded
V	Leukocidin-F subunit (Part of Panton Valentine Leukocidin)	P31715	*luk*F	GNFNPEFIGVLSR	725.380	531.420	19.90	37.0	conserved
V	Leukocidin-F subunit (Part of Panton Valentine Leukocidin)	P31715	*luk*F	GNFNPEFIGVLSR	725.380	791.480	19.90	37.0	conserved
V	Leukocidin-F subunit (Part of Panton Valentine Leukocidin)	P31715	*luk*F	GNFNPEFIGVLSR	725.380	920.520	19.90	37.0	conserved
	Leukocidin-F subunit (Part of Panton Valentine Leukocidin)	P31715	*luk*F	GNFNPEFIGVLSR	730.380	541.320	19.90	37.0	excluded
	Leukocidin-F subunit (Part of Panton Valentine	P31715	*luk*F	GNFNPEFIGVLSR	730.380	801.480	19.90	37.0	excluded

Leukocidin)								
Leukocidin-F subunit (Part of Panton Valentine Leukocidin)	P31715	*luk*F	GNFNPEFIGVLSR	730.380	930.520	19.90	37.0	excluded
Leukocidin-F subunit (Part of Panton Valentine Leukocidin)	P31715	*luk*F	ITVTYQR	440.750	567.290	10.40	24.0	excluded
Leukocidin-F subunit (Part of Panton Valentine Leukocidin)	P31715	*luk*F	ITVTYQR	440.750	666.360	10.40	24.0	excluded
Leukocidin-F subunit (Part of Panton Valentine Leukocidin)	P31715	*luk*F	ITVTYQR	440.750	767.400	10.40	24.0	excluded
Leukocidin-F subunit (Part of Panton Valentine Leukocidin)	P31715	*luk*F	ITVTYQR	445.750	577.290	10.40	24.0	excluded
Leukocidin-F subunit (Part of Panton Valentine Leukocidin)	P31715	*luk*F	ITVTYQR	445.750	676.360	10.40	24.0	excluded
Leukocidin-F subunit (Part of Panton Valentine Leukocidin)	P31715	*luk*F	ITVTYQR	445.750	777.400	10.40	24.0	excluded
Leukocidin-F subunit (Part of Panton Valentine	P31715	*luk*F	LIDTQSK	402.730	463.250	7.70	23.0	excluded

Leukocidin)								
Leukocidin-F subunit (Part of Panton Valentine Leukocidin)	P31715	*luk*F	LIDTQSK	402.730	578.280	7.70	23.0	excluded
Leukocidin-F subunit (Part of Panton Valentine Leukocidin)	P31715	*luk*F	LIDTQSK	402.730	691.360	7.70	23.0	excluded
Leukocidin-F subunit (Part of Panton Valentine Leukocidin)	P31715	*luk*F	LIDTQSK	406.730	471.250	7.70	23.0	excluded
Leukocidin-F subunit (Part of Panton Valentine Leukocidin)	P31715	*luk*F	LIDTQSK	406.730	586.280	7.70	23.0	excluded
Leukocidin-F subunit (Part of Panton Valentine Leukocidin)	P31715	*luk*F	LIDTQSK	406.730	699.360	7.70	23.0	excluded
Leukocidin-F subunit (Part of Panton Valentine Leukocidin)	P31715	*luk*F	MPVLSR	351.700	375.240	11.60	20.0	excluded
Leukocidin-F subunit (Part of Panton Valentine Leukocidin)	P31715	*luk*F	MPVLSR	351.700	474.300	11.60	20.0	excluded
Leukocidin-F subunit (Part of Panton Valentine	P31715	*luk*F	MPVLSR	351.700	571.360	11.60	20.0	excluded

	Leukocidin)								
	Leukocidin-F subunit (Part of Panton Valentine Leukocidin)	P31715	*luk*F	MPVLSR	356.700	385.240	11.60	20.0	excluded
	Leukocidin-F subunit (Part of Panton Valentine Leukocidin)	P31715	*luk*F	MPVLSR	356.700	484.300	11.60	20.0	excluded
	Leukocidin-F subunit (Part of Panton Valentine Leukocidin)	P31715	*luk*F	MPVLSR	356.700	581.360	11.60	20.0	excluded
V	Leukocidin-S subunit (Part of Panton Valentine Leukocidin)	P31716	*luk*S	ANSFITSLGK	519.280	618.400	15.60	28.0	conserved
V	Leukocidin-S subunit (Part of Panton Valentine Leukocidin)	P31716	*luk*S	ANSFITSLGK	519.280	765.450	15.60	28.0	conserved
V	Leukocidin-S subunit (Part of Panton Valentine Leukocidin)	P31716	*luk*S	ANSFITSLGK	519.280	852.480	15.60	28.0	conserved
	Leukocidin-S subunit (Part of Panton Valentine Leukocidin)	P31716	*luk*S	ANSFITSLGK	523.280	626.400	15.60	28.0	excluded
	Leukocidin-S subunit (Part of Panton Valentine	P31716	*luk*S	ANSFITSLGK	523.280	773.450	15.60	28.0	excluded

	Leukocidin)								
	Leukocidin-S subunit (Part of Panton Valentine Leukocidin)	P31716	*luk*S	ANSFITSLGK	523.280	860.480	15.60	28.0	excluded
	Leukocidin-S subunit (Part of Panton Valentine Leukocidin)	P31716	*luk*S	DALILK	336.720	373.300	14.08	20.0	excluded
	Leukocidin-S subunit (Part of Panton Valentine Leukocidin)	P31716	*luk*S	DALILK	336.720	486.360	14.08	20.0	excluded
	Leukocidin-S subunit (Part of Panton Valentine Leukocidin)	P31716	*luk*S	DALILK	336.720	557.400	14.08	20.0	excluded
	Leukocidin-S subunit (Part of Panton Valentine Leukocidin)	P31716	*luk*S	DALILK	340.720	381.300	14.08	20.0	excluded
	Leukocidin-S subunit (Part of Panton Valentine Leukocidin)	P31716	*luk*S	DALILK	340.720	494.360	14.08	20.0	excluded
	Leukocidin-S subunit (Part of Panton Valentine Leukocidin)	P31716	*luk*S	DALILK	340.720	565.400	14.08	20.0	excluded
V	Leukocidin-S subunit (Part of Panton Valentine	P31716	*luk*S	SVQWGIK	409.230	503.300	13.60	23.0	conserved

	Leukocidin)								
V	Leukocidin-S subunit (Part of Panton Valentine Leukocidin)	P31716	*luk*S	SVQWGIK	409.230	631.360	13.60	23.0	conserved
V	Leukocidin-S subunit (Part of Panton Valentine Leukocidin)	P31716	*luk*S	SVQWGIK	409.230	730.420	13.60	23.0	conserved
	Leukocidin-S subunit (Part of Panton Valentine Leukocidin)	P31716	*luk*S	SVQWGIK	413.230	511.300	13.60	23.0	excluded
	Leukocidin-S subunit (Part of Panton Valentine Leukocidin)	P31716	*luk*S	SVQWGIK	413.230	639.360	13.60	23.0	excluded
	Leukocidin-S subunit (Part of Panton Valentine Leukocidin)	P31716	*luk*S	SVQWGIK	413.230	738.420	13.60	23.0	excluded
V	Leukocidin-S subunit (Part of Panton Valentine Leukocidin)	P31716	*luk*S	TNDPNVDLINYLPK	808.420	643.400	20.00	40.0	conserved
V	Leukocidin-S subunit (Part of Panton Valentine Leukocidin)	P31716	*luk*S	TNDPNVDLINYLPK	808.420	860.500	20.00	40.0	conserved
V	Leukocidin-S subunit (Part of Panton Valentine	P31716	*luk*S	TNDPNVDLINYLPK	808.420	975.550	20.00	40.0	conserved

Leukocidin)								
Leukocidin-S subunit (Part of Panton Valentine Leukocidin)	P31716	*luk*S	TNDPNVDLINYLPK	812.420	647.400	20.00	40.0	excluded
Leukocidin-S subunit (Part of Panton Valentine Leukocidin)	P31716	*luk*S	TNDPNVDLINYLPK	812.420	868.500	20.00	40.0	excluded
Leukocidin-S subunit (Part of Panton Valentine Leukocidin)	P31716	*luk*S	TNDPNVDLINYLPK	812.420	983.550	20.00	40.0	excluded
Leukocidin-S subunit (Part of Panton Valentine Leukocidin)	P31716	*luk*S	YEVNWK	419.710	447.200	13.19	23.0	excluded
Leukocidin-S subunit (Part of Panton Valentine Leukocidin)	P31716	*luk*S	YEVNWK	419.710	546.300	13.19	23.0	excluded
Leukocidin-S subunit (Part of Panton Valentine Leukocidin)	P31716	*luk*S	YEVNWK	419.710	675.350	13.19	23.0	excluded
Leukocidin-S subunit (Part of Panton Valentine Leukocidin)	P31716	*luk*S	YEVNWK	423.710	455.200	13.19	23.0	excluded
Leukocidin-S subunit (Part of Panton Valentine	P31716	*luk*S	YEVNWK	423.710	554.300	13.19	23.0	excluded

Leukocidin)								
Leukocidin-S subunit (Part of Panton Valentine Leukocidin)	P31716	*luk*S	YEVNWK	423.710	683.350	13.19	23.0	excluded
Toxic shock syndrome toxin-1	P06886	*tst*	FEYNTEKPPINIDEIK	650.670	519.800	17.35	37.0	excluded
Toxic shock syndrome toxin-1	P06886	*tst*	FEYNTEKPPINIDEIK	650.670	755.910	17.35	37.0	excluded
Toxic shock syndrome toxin-1	P06886	*tst*	FEYNTEKPPINIDEIK	650.670	941.530	17.35	37.0	excluded
Toxic shock syndrome toxin-1	P06886	*tst*	HQLTQIHGLYR	683.370	645.350	12.59	35.0	excluded
Toxic shock syndrome toxin-1	P06886	*tst*	HQLTQIHGLYR	683.370	987.540	12.59	35.0	excluded
Toxic shock syndrome toxin-1	P06886	*tst*	HQLTQIHGLYR	683.370	1100.620	12.59	35.0	excluded
Toxic shock syndrome toxin-1	P06886	*tst*	IKNTDGSISLIIFPSPYYSPAFTK	887.140	537.270	22.89	48.0	excluded
Toxic shock syndrome toxin-1	P06886	*tst*	IKNTDGSISLIIFPSPYYSPAFTK	887.140	563.320	22.89	48.0	excluded
Toxic shock syndrome toxin-1	P06886	*tst*	IKNTDGSISLIIFPSPYYSPAFTK	887.140	1073.530	22.89	48.0	excluded

	Toxic shock syndrome toxin-1	P06886	*tst*	ITMNDGSTYQSDLSK	830.380	549.290	12.60	42.0	excluded
	Toxic shock syndrome toxin-1	P06886	*tst*	ITMNDGSTYQSDLSK	830.380	941.460	12.60	42.0	excluded
	Toxic shock syndrome toxin-1	P06886	*tst*	ITMNDGSTYQSDLSK	830.380	1085.510	12.60	42.0	excluded
	Toxic shock syndrome toxin-1	P06886	*tst*	KQLAISTLDFEIR	767.440	564.310	19.36	39.0	excluded
	Toxic shock syndrome toxin-1	P06886	*tst*	KQLAISTLDFEIR	767.440	980.500	19.36	39.0	excluded
	Toxic shock syndrome toxin-1	P06886	*tst*	KQLAISTLDFEIR	767.440	1093.590	19.36	39.0	excluded
V	Toxic shock syndrome toxin-1	P06886	*tst*	LPTPIELPLK	560.850	405.260	19.60	30.0	conserved
V	Toxic shock syndrome toxin-1	P06886	*tst*	LPTPIELPLK	560.850	809.510	19.60	30.0	conserved
V	Toxic shock syndrome toxin-1	P06886	*tst*	LPTPIELPLK	560.850	910.560	19.60	30.0	conserved
	Toxic shock syndrome toxin-1	P06886	*tst*	LPTPIELPLKVK	674.430	518.840	19.20	35.0	excluded
	Toxic shock syndrome toxin-1	P06886	*tst*	LPTPIELPLKVK	674.430	617.890	19.20	35.0	excluded

Toxic shock syndrome toxin-1	P06886	*tst*	LPTPIELPLKVK	674.430	1036.680	19.20	35.0	excluded
Toxic shock syndrome toxin-1	P06886	*tst*	NTDGSISLIIFPSPYYSPAFTK	806.750	537.270	23.98	40.0	excluded
Toxic shock syndrome toxin-1	P06886	*tst*	NTDGSISLIIFPSPYYSPAFTK	806.750	563.320	23.98	40.0	excluded
Toxic shock syndrome toxin-1	P06886	*tst*	NTDGSISLIIFPSPYYSPAFTK	806.750	1073.530	23.98	40.0	excluded
Toxic shock syndrome toxin-1	P06886	*tst*	NTDGSISLIIFPSPYYSPAFTKGEK	911.470	694.350	22.57	45.0	excluded
Toxic shock syndrome toxin-1	P06886	*tst*	NTDGSISLIIFPSPYYSPAFTKGEK	911.470	786.390	22.57	45.0	excluded
Toxic shock syndrome toxin-1	P06886	*tst*	NTDGSISLIIFPSPYYSPAFTKGEK	911.470	859.920	22.57	45.0	excluded
Toxic shock syndrome toxin-1	P06886	*tst*	QLAISTLDFEIR	703.390	564.310	21.14	36.0	excluded
Toxic shock syndrome toxin-1	P06886	*tst*	QLAISTLDFEIR	703.390	893.470	21.14	36.0	excluded
Toxic shock syndrome toxin-1	P06886	*tst*	QLAISTLDFEIR	703.390	980.500	21.14	36.0	excluded
Toxic shock syndrome toxin-1	P06886	*tst*	SSDKTGGYWK	564.770	610.300	10.10	30.0	excluded

Toxic shock syndrome toxin-1	P06886	*tst*	SSDKTGGYWK	564.770	711.350	10.10	30.0	excluded
Toxic shock syndrome toxin-1	P06886	*tst*	SSDKTGGYWK	564.770	954.470	10.10	30.0	excluded
Toxic shock syndrome toxin-1	P06886	*tst*	TGGYWK	356.180	496.300	10.71	21.0	excluded
Toxic shock syndrome toxin-1	P06886	*tst*	TGGYWK	356.180	553.280	10.71	21.0	excluded
Toxic shock syndrome toxin-1	P06886	*tst*	TGGYWK	356.180	610.300	10.71	21.0	excluded
Toxic shock syndrome toxin-1	P06886	*tst*	TIEAEIN	395.200	415.220	12.54	22.0	excluded
Toxic shock syndrome toxin-1	P06886	*tst*	TIEAEIN	395.200	526.250	12.54	22.0	excluded
Toxic shock syndrome toxin-1	P06886	*tst*	TIEAEIN	395.200	544.260	12.54	22.0	excluded
Toxic shock syndrome toxin-1	P06886	*tst*	VDLNTK	345.190	362.200	8.77	20.0	excluded
Toxic shock syndrome toxin-1	P06886	*tst*	VDLNTK	345.190	475.290	8.77	20.0	excluded
Toxic shock syndrome toxin-1	P06886	*tst*	VDLNTK	345.190	590.310	8.77	20.0	excluded

Toxic shock syndrome toxin-1	P06886	*tst*	VDLNTKR	423.250	518.300	8.11	24.0	excluded
Toxic shock syndrome toxin-1	P06886	*tst*	VDLNTKR	423.250	631.390	8.11	24.0	excluded
Toxic shock syndrome toxin-1	P06886	*tst*	VDLNTKR	423.250	746.420	8.11	24.0	excluded

Q1 (m/z): mass-to-charge ratio for the peptide ion used as Q1 value; Q3 m/z: mass-to-charge ratio for the fragment ion used as Q3 value.
[a]undetected, heavy or incongruous transitions were excluded based on train set analysis.
[b]Penicillin binding protein 2 prime stands for Penicillin Binding Protein 2 a (PBP2a), encoded by *mec*A[c] gene.
[c]according to Ito *et al.*[1].
[d]Beta-lactam-inducible penicillin-binding protein stands for Penicillin Binding Protein 2 c (PBP2c), encoded by *mec*C[c] gene.
[e]peptide FQITTSPGSTQK is shared by PBP2a and PBP2c encoded, respectively, by the *mec*A and *mec*C genes. This peptide was use to detected both proteins.

To facilitate the table reading, transitions contributing to a given feature (cf. column 1) have been highlighted by different colours:

- in green : I-feature;
- in orange: R-feature for PBP2a;
- in pink : R-feature for PBP2c;
- in grey: T-feature for Protein A;
- in dark blue: V-feature for PVL;
- in light blue: V-feature for TSST-1.

Supplementary Table 2: Datasets characterisation using refrence methods

Supplementary Table 2a: Train set characterisation

Sample name	Species[a]	*mec*A[b]	*mec*C[b]	*luk*S *luk*F[b]	*tst*[b]
Strain_001	*Staphylococcus aureus*	-	-	+	-
Strain_003	*Staphylococcus haemolyticus*	+	-	-	-
Strain_005	*Staphylococcus aureus*	-	-	-	-
Strain_006	*Staphylococcus simulans*	+	-	-	-
Strain_007	*Staphylococcus aureus*	+	-	-	-
Strain_009	*Staphylococcus aureus*	-	-	+	-
Strain_010	*Staphylococcus capitis*	-	-	-	-
Strain_012	*Staphylococcus saprophyticus*	+	-	-	-
Strain_014	*Staphylococcus epidermidis*	+	-	-	-
Strain_015	*Staphylococcus aureus*	+	-	-	-
Strain_019	*Staphylococcus aureus*	-	-	-	-
Strain_020	*Staphylococcus hominis*	+	-	-	-
Strain_023	*Staphylococcus aureus*	+	-	-	-
Strain_024	*Staphylococcus aureus*	+	-	-	-
Strain_027	*Staphylococcus aureus*	-	-	-	-
Strain_029	*Staphylococcus aureus*	+	-	-	-
Strain_031	*Staphylococcus aureus*	+	-	-	-
Strain_033	*Staphylococcus haemolyticus*	-	-	-	-
Strain_041	*Staphylococcus aureus*	+	-	-	-
Strain_043	*Staphylococcus aureus*	+	-	-	-
Strain_044	*Staphylococcus epidermidis*	-	-	-	-
Strain_046	*Staphylococcus saprophyticus*	-	-	-	-
Strain_047	*Staphylococcus aureus*	-	-	-	-

Strain_049	*Staphylococcus aureus*	-	-	+	-
Strain_051	*Staphylococcus aureus*	-	+	-	-
Strain_052	*Staphylococcus aureus*	-	+	-	-
Strain_053	*Staphylococcus aureus*	-	+	-	-
Strain_054	*Staphylococcus aureus*	-	+	-	-
Strain_055	*Staphylococcus aureus*	-	+	-	+
Strain_056	*Staphylococcus aureus*	-	+	-	-
Strain_057	*Staphylococcus aureus*	-	+	-	-
Strain_058	*Staphylococcus aureus*	-	+	-	-
Strain_087	*Staphylococcus aureus*	-	-	+	-
Strain_088	*Staphylococcus aureus*	+	-	+	-
Strain_089	*Staphylococcus aureus*	+	-	+	-
Strain_090	*Staphylococcus aureus*	-	-	+	-
Strain_091	*Staphylococcus aureus*	-	-	+	-
Strain_092	*Staphylococcus aureus*	+	-	+	-

[a]Species were identified by biochemical methods using VITEK2® instrument.

[b]*mec*A, *mec*C, *luk*F, *luk*S and *tst* genes were detected by PCR (see **Online methods**).

Supplementary Table 2b: Test set characterisation

Sample name	Species[a]	*mec*A[b]	*mec*C[b]	*luk*S *luk*F[b]	*tst*[b]
Strain_004	*Staphylococcus haemolyticus*	+	-	-	-
Strain_008	*Staphylococcus aureus*	+	-	-	-
Strain_011	*Staphylococcus aureus*	-	-	-	-
Strain_016	*Staphylococcus aureus*	-	-	-	-
Strain_017	*Staphylococcus aureus*	+	-	-	-
Strain_018	*Staphylococcus simulans*	+	-	-	-
Strain_021	*Staphylococcus capitis*	+	-	-	-
Strain_022	*Staphylococcus aureus*	-	-	-	+
Strain_025	*Staphylococcus aureus*	-	-	-	+
Strain_026	*Staphylococcus aureus*	+	-	-	-
Strain_028	*Staphylococcus hominis*	+	-	-	-
Strain_030	*Staphylococcus aureus*	-	-	-	-
Strain_032	*Staphylococcus aureus*	+	-	-	-
Strain_034	*Staphylococcus epidermidis*	+	-	-	-
Strain_035	*Staphylococcus warneri*	-	-	-	-
Strain_037	*Staphylococcus warneri*	-	-	-	-
Strain_038	*Staphylococcus aureus*	-	-	-	-
Strain_039	*Staphylococcus aureus*	+	-	-	-
Strain_040	*Staphylococcus hominis*	-	-	-	-
Strain_042	*Staphylococcus aureus*	+	-	-	-
Strain_045	*Staphylococcus simulans*	-	-	-	-
Strain_048	*Staphylococcus aureus*	-	-	-	-
Strain_050	*Staphylococcus aureus*	-	-	+	+
Strain_059	*Staphylococcus aureus*	-	+	-	+
Strain_060	*Staphylococcus aureus*	-	+	-	-
Strain_061	*Staphylococcus aureus*	-	+	-	-
Strain_062	*Staphylococcus aureus*	-	+	-	-
Strain_063	*Staphylococcus aureus*	-	+	-	-
Strain_064	*Staphylococcus aureus*	-	+	-	-
Strain_065	*Staphylococcus aureus*	-	+	-	-
Strain_066	*Staphylococcus aureus*	-	+	-	-
Strain_093	*Staphylococcus aureus*	+	-	+	-
Strain_094	*Staphylococcus aureus*	-	-	+	-
Strain_095	*Staphylococcus aureus*	+	-	+	+
Strain_096	*Staphylococcus aureus*	+	-	+	-
Strain_097	*Staphylococcus aureus*	-	-	+	-
Strain_098	*Staphylococcus aureus*	-	-	+	-
Strain_100	*Staphylococcus aureus*	-	-	+	+

[a]Species were identified by biochemical methods using VITEK2® instrument.
[b]*mec*A*,* *mec*C, *luk*F, *luk*S and *tst* genes were detected by PCR (see **Online methods**).

Supplementary Table 2c: characteristics of Staphylococcus aureus blind evaluation set 1

Sample name	*NRC*[a] *number*	Agr group[b]	CC or ST[c]	*spa* type[d]	*mec*A[e]	*mec*C[e]	*luk*S *luk*F[e]	*tst*[e]	*Strains features*	*Reference*
Strain_067	HT 2002 0209	3	ST80	t044	+	-	+	-	European CA-MRSA[f] clone	2
Strain_068	LUG 1800	3	ST80	t376	+	-	-	-	Δ*pvl* derivative of HT 2002 0209	3
Strain_069	HT 2005 0374	3	ST80	t934	-	-	+	-	ST80 *pvl*[+] MSSA[g] clone	4
Strain_070	HT 2003 0539	2	ST5	t450	+	-	+	-	/	4
Strain_071	HT 2003 0749	2	ST5	t242	+	-	-	+	MRSA[h] Geraldine clone	5
Strain_072	HT 2006 1141	2	ST5	t242	+	-	-	-	Δ*tst* derivative of HT2003 0749)	This work
Strain_073	HT 2004 0012	1	ST8	t008	+	-	-	-	HA-MRSA[i] Lyon clone	5
Strain_074	ST 2009 0070	1	ST8	t008	+	-	+	-	CA-MRSA USA 300 clone	6
Strain_075	HT 2007 0139	3	CC30	t012	-	-	-	+	CC30 MSSA lineage	7
Strain_076	ST 2009 0428	1	ST398	11	+	-	-	-	ST398 MRSA from a pig infection	8
Strain_077	LY 1999 0171	1	ST398	t571	-	-	+	-	ST398 MSSA from human infection	8
Strain_078	ST 2011 0820	3	ST1945	t843	-	+	-	-	*mec*C postive strain from human articular fluid	9
Strain_079	ST 2011 1100	3	ST130	t843	-	+	-	-	*mec*C postive strain from cow milk infection	9
Strain_080	ST 2011 1307	4	CC121	ND[j]	-	-	-	-	/	7
Strain_081	ST 2010 1105	2	ST5	2	+	-	-	+	HA-MRSA New York Japon clone	10
Strain_082	ST 2011 1020	1	ST239	30	+	-	-	-	HA-MRSA Hungarian and Brazilian clone	10
Strain_083	HT 2006 1122	2	CC5	2	+	-	-	-	HA-MRSA Pediatric clone	10
Strain_084	ST 2010 0143	1	CC22	32	+	-	-	-	UK MRSA15 clone	11
Strain_085	ST 2010 1055	3	CC36	12	+	-	-	+	UK MRSA16 clone	10
Strain_086	ST 2010 1992	1	CC45	4	+	-	-	-	HA-MRSA Berlin clone	12

[a]NRC number: strain number in French National Reference Center (NRC) of staphylococci.

[b]*Agr group*: allele of accessory gene regulator (Agr) system.
[c]CC or ST: clonal complex (CC) or sequence type (ST) according to multilocus sequence typing (MLST) scheme as previously described[13].
[d]*spa* type: type of A protein according to molecular typing methods using gene encoding A protein (*spa*) polymorphims[14].
[e]*mec*A and *mec*C encoding methicillin resistance, *luk*F and *luk*S genes encoding Panton Valentine Leukocidin and *tst* genes encoding Toxic Shock Syndrome Toxin 1 were detected by DNA microarrays as previously described[7].
[f]CA-MRSA: Community-Acquired Methicillin Resistant *Staphylococcus aureus.*
[g]MSSA: Methicillin Sensitive *Staphylococcus aureus.*
[h]MRSA: Methicillin Resistant *Staphylococcus aureus.*
[i]HA-MRSA: Hospital-Acquired Methicillin Resistant *Staphylococcus aureus.*
ND: not determined.

Results were given after SRMSRM analysis and interpretation

Supplementary Table 2d: characteristics of Staphylococcus aureus blind evaluation set 2

Sample name	*NRC reference*[a]	Agr group [b]	CC[c]	*mec*A[d]	*mec*C[d]	*luk*S, *luk*F[d]	*tst*[d]
Sample_101	ST 2012 0564	1	CC8	-	-	-	-
Sample_102	ST 2012 0529	1	CC22	-	-	-	-
Sample_103	ST 2012 0572	3	CC1	-	-	-	-
Sample_104	ST 2012 0523	1	CC20	-	-	-	-
Sample_105	ST 2012 0644	1	CC7	-	-	-	-
Sample_106	ST 2012 0566	1	CC398	-	-	-	-
Sample_107	ST 2012 0536	1	CC8	+	-	-	-
Sample_108	ST 2012 0533	1	CC20	-	-	-	-
Sample_109	ST 2012 0632	2	CC5	-	-	-	-
Sample_110	ST 2012 0641	2	CC5	-	-	-	-
Sample_111	ST 2012 1044	2	CC9	-	-	-	-
Sample_112	ST 2012 0520	1	CC8	-	-	-	-
Sample_113	ST 2012 0555	1	CC8	-	-	-	-
Sample_114	ST 2012 0586	1	CC188	-	-	-	-

[a]NRC reference: strain reference in French National Reference Center (NRC) of staphylococci.
[b]*Agr group*: allele of accessory gene regulator (Agr) system.
[c]CC or ST: clonal complex (CC) or sequence type (ST) were assigned by DNA microarrays as previously described.
[d]*mec*A and *mec*C encoding methicillin resistance, *luk*F and *luk*S genes encoding Panton Valentine Leukocidin and *tst* genes encoding Toxic Shock Syndrome Toxin 1 were detected by DNA microarrays as previously described[7].

Staphylococcus aureus was identified by WC-MALDI-TOF using VITEK-MS® instrument. Results were given after SRM analysis and interpretation.

Supplementary Table 2e: repeat set characterisation

Sample name	Number of repeat assay	Species[a]	*mec*A[b]	*mec*C[b]	*luk*S, *luk*F[b]	*tst*[b]
Strain_010_1	1	*Staphylococcus capitis*	-	-	-	-
Strain_010_2	2	*Staphylococcus capitis*	-	-	-	-
Strain_024_1	1	*Staphylococcus aureus*	+	-	-	-
Strain_024_2	2	*Staphylococcus aureus*	+	-	-	-
Strain_067_1	1	*Staphylococcus aureus*	+	-	+	-
Strain_067_2	2	*Staphylococcus aureus*	+	-	+	-
Strain_067_3	3	*Staphylococcus aureus*	+	-	+	-
Strain_070_1	1	*Staphylococcus aureus*	+	-	+	-
Strain_070_2	2	*Staphylococcus aureus*	+	-	+	-
Strain_070_3	3	*Staphylococcus aureus*	+	-	+	-
Strain_071_1	1	*Staphylococcus aureus*	+	-	-	+
Strain_071_2	2	*Staphylococcus aureus*	+	-	-	+
Strain_071_3	3	*Staphylococcus aureus*	+	-	-	+
Strain_075_1	1	*Staphylococcus aureus*	-	-	-	+
Strain_075_2	2	*Staphylococcus aureus*	-	-	-	+
Strain_075_3	3	*Staphylococcus aureus*	-	-	-	+
Strain_077_1	1	*Staphylococcus aureus*	-	-	+	-
Strain_077_2	2	*Staphylococcus aureus*	-	-	+	-
Strain_077_3	3	*Staphylococcus aureus*	-	-	+	-
Strain_078_1	1	*Staphylococcus aureus*	-	+	-	-
Strain_078_2	2	*Staphylococcus aureus*	-	+	-	-
Strain_078_3	3	*Staphylococcus aureus*	-	+	-	-
Strain_079_1	1	*Staphylococcus aureus*	-	+	-	-
Strain_079_2	2	*Staphylococcus aureus*	-	+	-	-
Strain_079_3	3	*Staphylococcus aureus*	-	+	-	-
Strain_080_1	1	*Staphylococcus aureus*	-	-	-	-
Strain_080_2	2	*Staphylococcus aureus*	-	-	-	-
Strain_080_3	3	*Staphylococcus aureus*	-	-	-	-
Strain_084_1	1	*Staphylococcus aureus*	+	-	-	-
Strain_084_2	2	*Staphylococcus aureus*	+	-	-	-
Strain_084_3	3	*Staphylococcus aureus*	+	-	-	-
Strain_085_1	1	*Staphylococcus aureus*	+	-	-	+
Strain_085_2	2	*Staphylococcus aureus*	+	-	-	+
Strain_085_3	3	*Staphylococcus aureus*	+	-	-	+

[a]Species were assessed according to **Supplementary Table 2a and 2c.**
[b]*mec*A and *mec*C encoding methicillin resistance, *luk*F and *luk*S genes encoding Panton Valentine Leukocidin and *tst* genes encoding toxic shock syndrome toxin were detected by PCR according to **Supplementary Table 2a and 2c**.

Results were given after SRM analysis and interpretation, except for both *Staphylococcus capitis* strains.

Supplementary Table 3: Results

Supplementary Table 3a: Train set results

Sample name	I-peptides : *S. aureus* identification				R-peptides: PBP2a (*mecA* product)[b]				R-peptides: PBP2a (*mecC* product)[d]				V-peptides: PVL				V-peptides: TSST				MS *versus* molecular methods agreement[g]
	1/slope	r	Number	MS result	1/slope	r	Number	MS result	1/slope	r	Number	MS result	1/slope	r	Number	MS result	1/slope	r	Number	MS result	
	1 ± 0,3	< 0,7	≤ 2		1 ± 0,3	< 0,7	≤ 3		1 ± 0,3	< 0,7	≤ 3		1 ± 0,3	< 0,7	≤ 1		1 ± 0,1	< 0,9	≤ 1		
Strain_001	1.008	0.998	9	+	0.189	0.230	3	-	0.079	0.073	1	-	0.970	0.980	4	+	0.000	0.000	0	-	Yes
Strain_005	0.981	0.998	9	+	-0.018	-0.030	2	-	0.000	0.000	0	-	0.000	0.000	0	-	0.000	0.000	0	-	Yes
Strain_051	0.995	0.999	9	+	0.101	0.140	4	-	1.056	0.988	5	+	0.000	0.000	0	-	0.000	0.000	0	-	Yes
Strain_052	0.973	0.997	8	+	0.154	0.222	2	-	0.891	0.990	3	+	0.000	0.000	0	-	0.000	0.000	0	-	Yes
Strain_049	1.019	0.999	8	+	0.214	0.351	4	-	-1.104	-0.602	1	-	0.985	0.999	4	+	0.000	0.000	0	-	Yes
Strain_087	0.991	0.997	8	+	-0.036	-0.078	1	-	0.000	0.000	0	-	0.952	0.994	4	+	0.000	0.000	0	-	Yes
Strain_015	1.016	0.999	8	+	0.979	0.995	8	+	0.634	0.722	3	-	0.000	0.000	0	-	0.000	0.000	0	-	Yes
Strain_007	0.990	0.996	9	+	0.946	0.996	8	+	0.861	0.995	1	-	0.000	0.000	0	-	0.000	0.000	0	-	Yes
Strain_044	1.424	0.687	2	-	0.581	0.789	2	-	-0.578	-1.000	1	-	0.000	0.000	0	-	0.000	0.000	0	-	Yes
Strain_046	0.000	0.000	0	-	0.000	0.000	0	-	0.000	0.000	0	-	0.000	0.000	0	-	0.000	0.000	0	-	Yes
Strain_009	1.006	0.999	9	+	-0.130	-0.199	3	-	-0.898	-0.659	1	-	1.014	0.998	4	+	0.000	0.000	0	-	Yes
Strain_053	1.002	0.999	9	+	0.345	0.347	6	-	1.059	0.982	5	+	0.000	0.000	0	-	0.000	0.000	0	-	Yes
Strain_054	0.961	0.998	8	+	0.053	0.090	3	-	0.937	0.998	3	+	0.000	0.000	0	-	0.000	0.000	0	-	Yes
Strain_090	0.994	1.000	9	+	0.179	0.189	6	-	-0.402	-0.763	3	-	1.026	0.999	4	+	0.000	0.000	0	-	Yes
Strain_091	0.988	0.998	9	+	-0.076	-0.133	2	-	0.000	0.000	0	-	0.960	0.994	4	+	0.000	0.000	0	-	Yes
Strain_029	0.991	1.000	9	+	0.994	0.985	8	+	-0.129	-0.254	3	-	0.000	0.000	0	-	0.000	0.000	0	-	Yes
Strain_023	0.982	0.996	8	+	0.948	0.995	8	+	0.866	1.000	1	-	0.000	0.000	0	-	0.000	0.000	0	-	Yes
Strain_003	0.000	0.000	0	-	0.944	0.994	8	+	0.148	0.181	2	-	0.000	0.000	0	-	-0.082	-0.125	1	-	Yes
Strain_006	1.123	0.925	1	-	0.989	0.997	8	+	0.466	0.763	3	-	0.000	0.000	0	-	0.000	0.000	0	-	Yes
Strain_010	0.000	0.000	0	-	0.099	0.318	3	-	0.242	0.361	2	-	0.429	0.543	2	-	0.000	0.000	0	-	Yes
Strain_033	0.000	0.000	0	-	-0.137	-0.316	1	-	-0.512	-0.554	2	-	0.000	0.000	0	-	-0.083	-0.134	1	-	Yes
Strain_019	1.006	1.000	9	+	0.072	0.078	7	-	-0.407	-0.767	3	-	0.000	0.000	0	-	0.756	0.933	1	-	Yes
Strain_024	1.005	0.999	9	+	1.009	0.996	8	+	-0.130	-0.218	3	-	0.000	0.000	0	-	0.000	0.000	0	-	Yes
Strain_055	0.997	0.999	9	+	0.129	0.162	6	-	1.089	0.933	5	+	0.000	0.000	0	-	1.000	1.000	1	+	Yes
Strain_056	1.014	0.999	8	+	0.025	0.029	5	-	0.998	0.882	5	+	0.000	0.000	0	-	0.000	0.000	0	-	Yes
Strain_088	0.999	0.999	9	+	1.020	0.998	8	+	0.550	0.524	2	-	1.000	0.997	4	+	0.730	0.895	1	-	Yes
Strain_089	0.992	0.999	9	+	1.021	0.997	8	+	0.457	0.397	2	-	1.017	0.995	4	+	0.851	0.922	1	-	Yes
Strain_041	0.996	0.999	9	+	0.994	0.995	8	+	0.886	0.827	2	-	0.000	0.000	0	-	0.000	0.000	0	-	Yes
Strain_031	0.990	0.999	9	+	1.005	0.968	8	+	-0.156	-0.257	2	-	0.000	0.000	0	-	0.538	0.595	1	-	Yes
Strain_012	0.000	0.000	0	-	0.733	0.758	7	+	0.253	0.251	2	-	0.000	0.000	0	-	0.000	0.000	0	-	Yes
Strain_014	-2.191	-0.908	1	-	0.804	0.895	7	+	0.508	0.632	2	-	0.000	0.000	0	-	0.000	0.000	0	-	Yes
Strain_027	1.007	0.999	9	+	0.152	0.195	4	-	-0.430	-0.643	3	-	0.000	0.000	0	-	0.000	0.000	0	-	Yes
Strain_047	0.996	0.998	8	+	0.048	0.058	3	-	-0.519	-0.707	3	-	0.000	0.000	0	-	0.000	0.000	0	-	Yes
Strain_057	1.008	0.998	9	+	0.012	0.016	4	-	0.944	0.899	5	+	0.000	0.000	0	-	0.000	0.000	0	-	Yes
Strain_058	1.010	0.999	9	+	0.262	0.360	5	-	0.842	0.836	5	+	0.000	0.000	0	-	0.000	0.000	0	-	Yes
Strain_092	1.000	0.998	9	+	1.031	0.997	8	+	0.595	0.462	2	-	0.968	0.993	4	+	0.752	0.870	1	-	Yes
Strain_043	1.000	0.998	9	+	1.012	0.995	8	+	1.100	0.983	1	-	0.000	0.000	0	-	0.724	0.815	1	-	Yes
Strain_020	0.000	0.000	0	-	0.938	0.981	8	+	-0.049	-0.053	3	-	0.000	0.000	0	-	0.000	0.000	0	-	Yes

[a]PBP2a: Penicillin Binding Protein 2 a, encoded by *mec*A gene.

[b]PBP2c: Penicillin Binding Protein 2 c, encoded by *mec*C gene.

[c]PVL: Panton Valentine Leukocidin.

[d]TSST-1: Toxic Shock Syndrome Toxin 1.

[e]Reference methods: full automated biochemical VITEK2® method for identification, PCR test for *mec*A*, mec*C, *luk*F, *luk*S and *tst* genes detection (see **Supplementary Table 2a**).

Supplementary Table 3b: Test set results

Sample name	I-peptides : *S. aureus* identification				R-peptides: PBP2a (*mecA* product)[b]				R-peptides: PBP2a (*mecC* product)[d]				V-peptides: PVL				V-peptides: TSST				MS *versus* molecular methods agreement[g]
	1/slope 1 ± 0,3	r < 0,7	Number ≤ 2	MS result	1/slope 1 ± 0,3	r < 0,7	Number ≤ 3	MS result	1/slope 1 ± 0,3	r < 0,7	Number ≤ 3	MS result	1/slope 1 ± 0,3	r < 0,7	Number ≤ 1	MS result	1/slope 1 ± 0,1	r < 0,9	Number ≤ 1	MS result	
Strain_035	0.000	0.000	0	-	0.274	0.566	3	-	0.276	0.214	2	-	1.823	0.990	1	-	0.000	0.000	0	-	Yes
Strain_040	-0.359	-0.518	2	-	-0.009	-0.011	5	-	-0.355	-0.436	3	-	-0.990	-0.944	1	-	0.146	0.169	1	-	Yes
Strain_011	1.006	0.997	9	+	0.289	0.421	4	-	-0.456	-0.997	1	-	0.000	0.000	0	-	0.000	0.000	0	-	Yes
Strain_016	1.038	0.994	9	+	0.253	0.379	4	-	-0.409	-0.793	3	-	0.000	0.000	0	-	-0.757	-0.585	1	-	Yes
Strain_060	1.041	0.994	9	+	0.264	0.325	5	-	1.039	0.983	5	+	0.388	0.917	1	-	-0.423	-0.670	1	-	Yes
Strain_061	1.036	0.993	9	+	-0.036	-0.041	6	-	1.041	0.982	5	+	0.000	0.000	0	-	0.000	0.000	0	-	Yes
Strain_094	1.024	0.995	9	+	0.198	0.262	5	-	-0.598	-0.755	3	-	0.991	0.974	4	+	1.687	0.902	1	-	Yes
Strain_097	1.041	0.993	8	+	0.241	0.319	5	-	-0.361	-0.820	2	-	1.129	0.991	3	+	0.000	0.000	0	-	Yes
Strain_026	1.007	0.995	9	+	0.945	0.998	8	+	-0.149	-0.279	3	-	0.000	0.000	0	-	0.935	0.868	1	-	Yes
Strain_017	0.994	0.996	9	+	0.257	0.370	4	-	-0.358	-0.988	2	-	0.000	0.000	0	-	0.953	0.976	1	+	Yes
Strain_045	0.218	0.324	2	-	0.213	0.244	4	-	0.519	0.812	2	-	0.571	0.730	2	-	0.000	0.000	0	-	Yes
Strain_004	0.000	0.000	0	-	0.955	0.993	8	+	-0.276	-0.343	3	-	0.567	0.790	1	-	-0.066	-0.122	1	-	Yes
Strain_022	1.020	0.995	9	+	0.935	0.996	8	+	-0.099	-0.183	3	-	0.000	0.000	0	-	0.000	0.000	0	-	Yes
Strain_025	1.003	0.995	9	+	0.215	0.303	5	-	-0.275	-0.456	3	-	0.000	0.000	0	-	0.972	0.981	1	+	Yes
Strain_062	1.024	0.993	9	+	0.296	0.432	6	-	1.089	0.894	5	+	0.000	0.000	0	-	0.000	0.000	0	-	Yes
Strain_063	1.048	0.991	9	+	0.187	0.268	4	-	0.941	0.991	5	+	0.000	0.000	0	-	0.000	0.000	0	-	Yes
Strain_098	1.029	0.996	8	+	0.281	0.439	4	-	-0.312	-0.861	2	-	0.872	0.971	4	+	0.000	0.000	0	-	Yes
Strain_093	1.041	0.993	8	+	0.959	0.996	8	+	-0.104	-0.214	3	-	0.973	0.990	4	+	0.000	0.000	0	-	Yes
Strain_039	1.019	0.995	9	+	0.937	0.992	8	+	-0.119	-0.241	2	-	0.000	0.000	0	-	0.000	0.000	0	-	Yes
Strain_032	1.030	0.995	9	+	0.871	0.928	8	+	-0.161	-0.311	3	-	0.000	0.000	0	-	0.000	0.000	0	-	Yes
Strain_018	0.061	0.052	3	-	0.957	0.997	8	+	0.453	0.718	4	-	0.556	0.692	2	-	0.000	0.000	0	-	Yes
Strain_021	0.457	0.531	3	-	0.933	0.978	8	+	-0.311	-0.418	4	-	-0.555	-0.518	2	-	0.000	0.000	0	-	Yes
Strain_030	1.034	0.994	9	+	0.279	0.417	4	-	-0.481	-0.828	3	-	0.000	0.000	0	-	0.000	0.000	0	-	Yes
Strain_038	1.033	0.994	9	+	0.086	0.142	5	-	-0.349	-0.994	2	-	-1.601	-0.553	1	-	0.000	0.000	0	-	Yes
Strain_064	1.038	0.994	9	+	0.308	0.430	6	-	1.038	0.971	5	+	0.000	0.000	0	-	0.000	0.000	0	-	Yes
Strain_065	1.034	0.995	9	+	0.308	0.430	5	-	0.979	0.976	5	+	0.000	0.000	0	-	0.000	0.000	0	-	Yes
Strain_095	1.019	0.994	9	+	0.932	0.995	8	+	-0.133	-0.261	3	-	0.920	0.983	4	+	0.000	0.000	0	-	No TSST-1 issue[i]
Strain_096	1.029	0.994	9	+	0.940	0.995	8	+	0.151	0.179	3	-	1.003	0.988	4	+	0.000	0.000	0	-	Yes
Strain_042	1.027	0.982	9	+	0.939	0.995	8	+	-0.124	-0.230	3	-	0.000	0.000	0	-	1.065	0.870	1	-	Yes
Strain_028	1.175	0.945	1	-	0.764	0.823	7	+	0.386	0.545	2	-	-0.303	-0.278	1	-	0.000	0.000	0	-	Yes
Strain_034	0.000	0.000	0	-	0.919	0.965	8	+	-0.253	-0.347	4	-	0.000	0.000	0	-	0.000	0.000	0	-	Yes
Strain_048	1.032	0.997	8	+	0.098	0.155	5	-	-0.503	-0.781	3	-	0.000	0.000	0	-	0.000	0.000	0	-	Yes
Strain_059	1.034	0.912	9	+	0.227	0.344	5	-	1.013	0.954	5	+	0.000	0.000	0	-	0.973	0.980	1	+	Yes
Strain_066	1.039	0.994	9	+	0.047	0.044	5	-	1.077	0.986	5	+	0.000	0.000	0	-	-0.120	-0.096	1	-	Yes
Strain_050	1.003	0.958	9	+	0.289	0.378	5	-	0.054	0.076	3	-	0.953	0.991	4	+	0.961	0.981	1	+	Yes
Strain_008	1.011	0.995	9	+	0.928	0.997	8	+	0.073	0.108	3	-	0.000	0.000	0	-	0.000	0.000	0	-	Yes
Strain_100	1.002	0.965	9	+	0.242	0.344	6	-	-0.168	-0.312	3	-	0.909	0.968	4	+	0.976	0.971	1	+	Yes
Strain_037	0.000	0.000	0	-	0.604	0.752	2	-	-0.990	-0.706	2	-	0.408	0.468	2	-	0.000	0.000	0	-	Yes

[a]PBP2a: Penicillin Binding Protein 2 a, encoded by *mec*A gene.

[b]PBP2c: Penicillin Binding Protein 2 c, encoded by *mec*C gene.

[c]PVL: Panton Valentine Leukocidin.

[d]TSST-1: Toxic Shock Syndrome Toxin 1.

[e]Reference methods: full automated biochemical VITEK2® method for identification, PCR test for *mec*A*, mec*C, *luk*F, *luk*S and *tst* genes detection (see **Supplementary Table 2b**).

[f]Strain_095 harboring TSST-1 gene was seen as negative for TSST-1 by SRM. However, sequencing of TSST-1 gene detected a mutation in the proteotypic peptide selected for the SRM method. LPTPIELPLK peptide was mutated into LLTPIELPLK peptide. The corresponding precursor masses are different and the LLTPIELPLK peptide detection was impossible with the SRM method described in Supplementary table 1. Nevertheless, the current SRM method should be easily modified to integrate the transitions of the mutated peptide (data not shown).

Supplementary Table 3c: Results of blind evaluation with set 1 strains

Sample name	I-peptides : *S. aureus* identification				R-peptides: PBP2a (*mecA* product)[b]				R-peptides: PBP2a (*mecC* product)[d]				V-peptides: PVL				V-peptides: TSST				MS *versus* molecular methods agreement[g]
	1/slope	r	Number	MS result	1/slope	r	Number	MS result	1/slope	r	Number	MS result	1/slope	r	Number	MS result	1/slope	r	Number	MS result	
	1 ± 0,3	< 0,7	≤ 2		1 ± 0,3	< 0,7	≤ 3		1 ± 0,3	< 0,7	≤ 3		1 ± 0,3	< 0,7	≤ 1		1 ± 0,1	< 0,9	≤ 1		
Strain_067	1.009	0.996	9	+	0.932	0.996	8	+	-0.019	-0.028	2	-	0.961	0.990	4	+	0.000	0.000	0	-	Yes
Strain_068	1.019	0.996	9	+	0.917	0.994	8	+	-0.096	-0.179	2	-	0.000	0.000	0	-	0.880	0.988	1	-	Yes
Strain_069	1.012	0.997	9	+	0.295	0.415	7	-	-0.349	-0.653	3	-	0.960	0.988	4	+	0.000	0.000	0	-	Yes
Strain_070	1.018	0.989	9	+	0.943	0.993	8	+	-0.090	-0.162	2	-	0.894	0.985	4	+	0.000	0.000	0	-	Yes
Strain_071	1.013	0.996	9	+	0.933	0.996	8	+	0.382	0.586	2	-	-0.689	-0.840	1	-	1.040	0.984	1	+	Yes
Strain_072	0.999	0.996	9	+	0.928	0.990	8	+	0.167	0.287	3	-	0.000	0.000	0	-	0.000	0.000	0	-	Yes
Strain_073	0.986	0.996	9	+	0.895	0.993	8	+	0.328	0.616	2	-	0.000	0.000	0	-	0.783	0.345	1	-	Yes
Strain_074	1.013	0.997	9	+	0.917	0.995	8	+	0.284	0.459	3	-	0.934	0.985	4	+	0.000	0.000	0	-	Yes
Strain_075	0.999	0.996	8	+	0.232	0.357	6	-	0.039	0.042	3	-	0.000	0.000	0	-	1.020	0.983	1	+	Yes
Strain_076	1.012	0.998	9	+	0.929	0.996	8	+	0.270	0.373	3	-	0.000	0.000	0	-	0.000	0.000	0	-	Yes
Strain_077	1.003	0.996	9	+	0.209	0.341	4	-	-0.404	-0.757	2	-	0.940	0.991	4	+	0.000	0.000	0	-	Yes
Strain_078	1.040	0.994	9	+	0.159	0.231	4	-	0.996	0.959	5	+	0.000	0.000	0	-	0.000	0.000	0	-	Yes
Strain_079	1.017	0.997	9	+	0.364	0.458	6	-	0.988	0.946	5	+	0.000	0.000	0	-	0.000	0.000	0	-	Yes
Strain_080	1.006	0.991	8	+	0.342	0.457	4	-	0.000	0.000	0	-	0.000	0.000	0	-	0.000	0.000	0	-	Yes
Strain_081	0.997	0.998	9	+	0.925	0.996	8	+	-0.023	-0.034	2	-	0.000	0.000	0	-	0.975	0.986	1	+	Yes
Strain_082	1.007	0.997	9	+	0.919	0.992	8	+	0.283	0.423	3	-	0.000	0.000	0	-	0.000	0.000	0	-	Yes
Strain_083	1.027	0.996	7	+	0.943	0.996	8	+	0.001	0.001	2	-	0.000	0.000	0	-	0.000	0.000	0	-	Yes
Strain_084	0.842	0.872	7	+	0.817	0.833	8	+	0.129	0.164	3	-	0.291	0.229	2	-	0.000	0.000	0	-	Yes
Strain_085	1.011	0.997	9	+	0.955	0.994	8	+	0.080	0.100	3	-	0.000	0.000	0	-	0.998	0.987	1	+	Yes
Strain_086	1.005	0.996	8	+	0.928	0.994	8	+	-0.031	-0.044	3	-	-0.549	-0.536	1	-	0.000	0.000	0	-	Yes

[a]PBP2a: Penicillin Binding Protein 2 a, encoded by *mec*A gene.
[b]PBP2c: Penicillin Binding Protein 2 c, encoded by *mec*C gene.
[c]PVL: Panton Valentine Leukocidin.
[d]TSST-1: Toxic Shock Syndrome Toxin 1.
[e]Reference methods: WC-MALDI-TOF or full automated biochemical VITEK2® method for identification, PCR test for *mec*A*,* *mec*C, *luk*F, *luk*S and *tst* genes detection (see **Supplementary Table 2c**).

Supplementary Table 3d: Results of blind evaluation with set 2 strains

Sample name	I-peptides : *S. aureus* identification				R-peptides: PBP2a (*mecA* product)[b]				R-peptides: PBP2a (*mecC* product)[d]				V-peptides: PVL				V-peptides: TSST				MS *versus* molecular methods agreement[g]
	1/slope	r	Number	MS result	1/slope	r	Number	MS result	1/slope	r	Number	MS result	1/slope	r	Number	MS result	1/slope	r	Number	MS result	
	1 ± 0,3	< 0,7	≤ 2		1 ± 0,3	< 0,7	≤ 3		1 ± 0,3	< 0,7	≤ 3		1 ± 0,3	< 0,7	≤ 1		1 ± 0,1	< 0,9	≤ 1		
Sample_101	0.884	0.990	4	+	0.106	0.216	2	-	0.000	0.000	0	-	0.000	0.000	0	-	0.000	0.000	0	-	Yes
Sample_102	0.866	0.994	3	+	0.064	0.126	2	-	0.000	0.000	0	-	0.000	0.000	0	-	0.000	0.000	0	-	Yes
Sample_103	0.852	0.986	3	+	0.262	0.236	1	-	0.000	0.000	0	-	0.000	0.000	0	-	0.000	0.000	0	-	Yes
Sample_104	0.908	0.992	5	+	0.754	0.508	1	-	0.000	0.000	0	-	0.000	0.000	0	-	0.000	0.000	0	-	Yes
Sample_105	0.857	0.990	4	+	0.485	0.267	1	-	0.000	0.000	0	-	0.000	0.000	0	-	0.000	0.000	0	-	Yes
Sample_106	0.886	0.996	3	+	0.212	0.160	1	-	0.000	0.000	0	-	0.000	0.000	0	-	0.000	0.000	0	-	Yes
Sample_107	0.896	0.993	6	+	0.910	0.976	8	+	0.712	0.993	1	-	0.000	0.000	0	-	0.000	0.000	0	-	Yes
Sample_108	0.888	0.995	4	+	0.409	0.304	1	-	0.000	0.000	0	-	0.000	0.000	0	-	0.000	0.000	0	-	Yes
Sample_109	0.821	0.989	5	+	0.031	0.025	1	-	0.000	0.000	0	-	0.000	0.000	0	-	0.000	0.000	0	-	Yes
Sample_110	0.917	0.991	3	+	0.097	0.239	1	-	0.000	0.000	0	-	0.000	0.000	0	-	0.000	0.000	0	-	Yes
Sample_111	0.873	0.996	4	+	1.784	1.000	1	-	0.000	0.000	0	-	0.000	0.000	0	-	0.000	0.000	0	-	Yes
Sample_112	0.890	0.992	2	+	0.000	0.000	0	-	0.000	0.000	0	-	0.000	0.000	0	-	0.000	0.000	0	-	Yes
Sample_113	0.895	0.996	2	+	0.000	0.000	0	-	0.000	0.000	0	-	0.000	0.000	0	-	0.000	0.000	0	-	Yes
Sample_114	0.874	0.990	5	+	1.333	0.717	1	-	0.000	0.000	0	-	0.000	0.000	0	-	0.000	0.000	0	-	Yes

[a]PBP2a: Penicillin Binding Protein 2 a, encoded by *mec*A gene.

[b]PBP2c: Penicillin Binding Protein 2 c, encoded by *mec*C gene.

[c]PVL: Panton Valentine Leukocidin.

[d]TSST-1: Toxic Shock Syndrome Toxin 1.

[e]Reference methods: WC-MALDI-TOF for identification, PCR test for *mecA, mecC*, *lukF*, *lukS* and *tst* genes detection (see **Supplementary Table 2d**).

Supplementary Table 3e: Results of repeat set strains

Sample name	I-peptides : *S. aureus* identification				R-peptides: PBP2a (*mecA* product)[b]				R-peptides: PBP2a (*mecC* product)[d]				V-peptides: PVL				V-peptides: TSST				MS *versus* molecular methods agreement[g]
	1/slope	r	Number	MS result	1/slope	r	Number	MS result	1/slope	r	Number	MS result	1/slope	r	Number	MS result	1/slope	r	Number	MS result	
	1 ± 0,3	< 0,7	≤ 2		1 ± 0,3	< 0,7	≤ 3		1 ± 0,3	< 0,7	≤ 3		1 ± 0,3	< 0,7	≤ 1		1 ± 0,1	< 0,9	≤ 1		
Strain_067_1	0.962	0.994	9	+	0.931	0.990	8	+	-0.060	-0.112	2	-	1.049	0.981	2	+	0.000	0.000	0	-	Yes
Strain_071_1	0.977	0.994	9	+	0.970	0.990	8	+	0.849	0.995	1	-	0.361	0.940	1	-	1.059	0.990	1	+	Yes
Strain_075_1	0.981	0.991	8	+	0.226	0.376	4	-	0.100	0.057	1	-	0.000	0.000	0	-	1.027	0.983	1	+	Yes
Strain_077_1	0.955	0.994	9	+	0.529	0.945	3	-	-0.488	-0.999	1	-	1.089	0.917	1	+	0.000	0.000	0	-	Yes
Strain_078_1	0.985	0.994	9	+	-0.090	-0.113	5	-	0.986	0.983	5	+	0.000	0.000	0	-	0.000	0.000	0	-	Yes
Strain_079_1	0.972	0.994	9	+	0.401	0.662	5	-	0.934	0.987	5	+	0.379	0.923	1	-	0.000	0.000	0	-	Yes
Strain_080_1	0.971	0.996	9	+	0.342	0.572	4	-	-0.452	-0.999	1	-	0.000	0.000	0	-	0.000	0.000	0	-	Yes
Strain_084_1	0.964	0.995	9	+	0.935	0.993	8	+	0.805	0.994	1	-	0.000	0.000	0	-	-0.725	-0.652	1	-	Yes
Strain_085_1	0.976	0.993	9	+	0.925	0.993	8	+	0.325	0.463	3	-	0.000	0.000	0	-	1.073	0.987	1	+	Yes
Strain_010_1	0.925	0.576	1	-	0.001	0.001	5	-	0.086	0.139	2	-	0.000	0.000	0	-	0.000	0.000	0	-	Yes
Strain_024_1	0.973	0.994	9	+	0.941	0.992	8	+	0.490	0.534	3	-	0.000	0.000	0	-	0.000	0.000	0	-	Yes
Strain_070_1	0.989	0.994	9	+	0.942	0.996	8	+	-0.068	-0.110	2	-	1.025	0.897	1	+	0.000	0.000	0	-	Yes
Strain_067_2	0.970	0.994	9	+	0.916	0.993	8	+	0.823	0.994	1	-	1.114	0.987	2	+	0.000	0.000	0	-	Yes
Strain_070_2	0.982	0.992	9	+	0.915	0.994	8	+	0.294	0.359	2	-	1.054	0.998	1	+	0.000	0.000	0	-	Yes
Strain_071_2	0.967	0.994	9	+	0.950	0.994	8	+	-0.058	-0.099	2	-	0.000	0.000	0	-	0.939	0.986	1	+	Yes
Strain_075_2	0.969	0.996	8	+	0.331	0.502	6	-	-0.068	-0.059	2	-	0.000	0.000	0	-	0.968	0.980	1	+	Yes
Strain_077_2	0.964	0.989	9	+	0.168	0.339	3	-	-0.520	-0.996	1	-	1.031	0.972	2	+	0.000	0.000	0	-	Yes
Strain_078_2	0.982	0.993	9	+	0.036	0.052	5	-	0.951	0.973	5	+	0.000	0.000	0	-	0.000	0.000	0	-	Yes
Strain_079_2	0.981	0.994	9	+	0.285	0.495	4	-	0.928	0.992	5	+	0.564	0.743	1	-	0.000	0.000	0	-	Yes
Strain_080_2	0.987	0.995	9	+	0.120	0.187	3	-	-0.403	-0.999	1	-	0.000	0.000	0	-	0.000	0.000	0	-	Yes
Strain_084_2	0.976	0.992	9	+	0.945	0.994	8	+	0.249	0.297	2	-	0.355	0.936	1	-	0.000	0.000	0	-	Yes
Strain_085_2	0.962	0.992	9	+	0.923	0.993	8	+	0.574	0.706	2	-	0.000	0.000	0	-	0.970	0.990	1	+	Yes
Strain_010_3	0.000	0.000	0	-	0.168	0.439	2	-	0.000	0.000	0	-	0.000	0.000	0	-	0.000	0.000	0	-	Yes
Strain_024_3	0.984	0.993	9	+	0.945	0.987	8	+	-0.068	-0.106	2	-	0.000	0.000	0	-	0.000	0.000	0	-	Yes
Strain_067_3	0.974	0.993	9	+	0.931	0.994	8	+	0.016	0.023	3	-	1.001	0.987	3	+	0.000	0.000	0	-	Yes
Strain_070_3	0.986	0.995	9	+	0.928	0.994	8	+	0.070	0.097	2	-	1.154	0.986	1	+	0.000	0.000	0	-	Yes
Strain_071_3	0.984	0.994	9	+	0.952	0.995	8	+	-0.061	-0.100	2	-	0.000	0.000	0	-	0.942	0.987	1	+	Yes
Strain_075_3	0.963	0.994	8	+	0.149	0.243	4	-	-0.033	-0.035	2	-	0.000	0.000	0	-	0.986	0.982	1	+	Yes
Strain_077_3	0.977	0.993	9	+	0.253	0.499	3	-	0.000	0.000	0	-	1.010	0.987	3	+	0.000	0.000	0	-	Yes
Strain_078_3	0.971	0.994	9	+	0.163	0.261	4	-	0.847	0.848	5	+	0.000	0.000	0	-	0.000	0.000	0	-	Yes
Strain_079_3	0.987	0.995	9	+	0.299	0.497	4	-	0.942	0.990	5	+	0.383	0.930	1	-	0.000	0.000	0	-	Yes
Strain_080_3	0.972	0.993	9	+	0.342	0.539	4	-	-0.213	-0.369	2	-	0.000	0.000	0	-	0.000	0.000	0	-	Yes
Strain_084_3	0.980	0.994	9	+	0.949	0.994	8	+	-0.004	-0.006	2	-	0.000	0.000	0	-	0.000	0.000	0	-	Yes
Strain_085_3	0.967	0.992	9	+	0.931	0.993	8	+	0.590	0.728	2	-	0.000	0.000	0	-	1.009	0.985	1	+	Yes

[a]PBP2a: Penicillin Binding Protein 2 a, encoded by *mec*A gene.

[b]PBP2c: Penicillin Binding Protein 2 c, encoded by *mec*C gene.

[c]PVL: Panton Valentine Leukocidin.

[d]TSST-1: Toxic Shock Syndrome Toxin 1.

[e]Reference methods: full automated biochemical VITEK2® method for identification, PCR test for *mec*A, *mec*C, *luk*F, *luk*S and *tst* genes detection (see **Supplementary Table 2a and 2c**).

Supplementary Table 4: Typing information, Staphylococcus aureus blind evaluation set 1

The number of peptides shared between strains was counted using I-Peptides and T-Peptides (cf. **Supplementary Table 1**). The higher the number is the closer the strains are.

Sample	STAAU _067	STAAU _068	STAAU _069	STAAU _070	STAAU _071	STAAU _072	STAAU _073	STAAU _074	STAAU _075	STAAU _076	STAAU _077	STAAU_ 078	STAAU _079	STAAU _080	STAAU _081	STAAU _082	STAAU _083	STAAU _084	STAAU _085	STAAU _086
STAAU_067	24	24	24	21	18	15	19	23	19	17	20	21	22	17	22	21	18	16	22	20
STAAU_068	24	24	24	21	18	15	19	23	19	17	20	21	22	17	22	21	18	16	22	20
STAAU_069	24	24	24	21	18	15	19	23	19	17	20	21	22	17	22	21	18	16	22	20
STAAU_070	21	21	21	24	17	16	18	22	20	18	19	22	21	16	21	20	19	15	21	17
STAAU_071	18	18	18	17	24	17	21	19	17	19	18	15	18	17	18	19	18	16	18	18
STAAU_072	15	15	15	16	17	24	20	16	12	22	17	14	17	16	15	16	13	15	15	13
STAAU_073	19	19	19	18	21	20	24	20	16	22	21	16	19	16	19	20	17	19	19	17
STAAU_074	23	23	23	22	19	16	20	24	20	18	21	20	21	16	23	22	19	17	23	19
STAAU_075	19	19	19	20	17	12	16	20	24	14	19	20	17	14	19	18	21	13	21	19
STAAU_076	17	17	17	18	19	22	22	18	14	24	19	16	19	18	17	18	15	17	17	15
STAAU_077	20	20	20	19	18	17	21	21	19	19	24	19	20	13	20	19	16	18	22	16
STAAU_078	21	21	21	22	15	14	16	20	20	16	19	24	21	16	19	18	17	13	21	17
STAAU_079	22	22	22	21	18	17	19	21	17	19	20	21	24	17	20	19	16	16	20	18
STAAU_080	17	17	17	16	17	16	16	16	14	18	13	16	17	24	15	18	15	13	15	17
STAAU_081	22	22	22	21	18	15	19	23	19	17	20	19	20	15	24	21	18	18	22	18
STAAU_082	21	21	21	20	19	16	20	22	18	18	19	18	19	18	21	24	19	17	21	19
STAAU_083	18	18	18	19	18	13	17	19	21	15	16	17	16	15	18	19	24	14	18	20
STAAU_084	16	16	16	15	16	15	19	17	13	17	18	13	16	13	18	17	14	24	16	14
STAAU_085	22	22	22	21	18	15	19	23	21	17	22	21	20	15	22	21	18	16	24	18
STAAU_086	20	20	20	17	18	13	17	19	19	15	16	17	18	17	18	19	20	14	18	24
CC[a] or ST[b]	ST80	ST80	ST80	ST5	ST5	ST5	ST8	ST8	CC30	ST398	ST398	ST1945	ST130	CC121	ST5	ST239	CC5	CC22	CC36	CC45
spa type	t044	t376	t934	t450	t242	t242	t008	t008	t012	ND	t571	t843	t843	ND	ND	ND	ND	ND	ND	ND

[a]CC: clonal complex
[b]ST: sequence type.

Supplementary Figure 1: Screenshot of Strain_067, analysed in blind conditions and identified as S. aureus, expressing both Penicillin Binding Protein 2a (PBP2a) and Panton-Valentine Leukocidin (PVL), but neither Penicillin Binding Protein 2c (PBP2c), nor Toxic Shock Syndrome Toxin 1 (TSST-1).

Peptides used for strain identification, resistance markers and virulence factors are identified by green, red and blue stars, respectively. Black crosses show isolated transitions.

A - Extracted ion chromatogram of peptides used for identification.

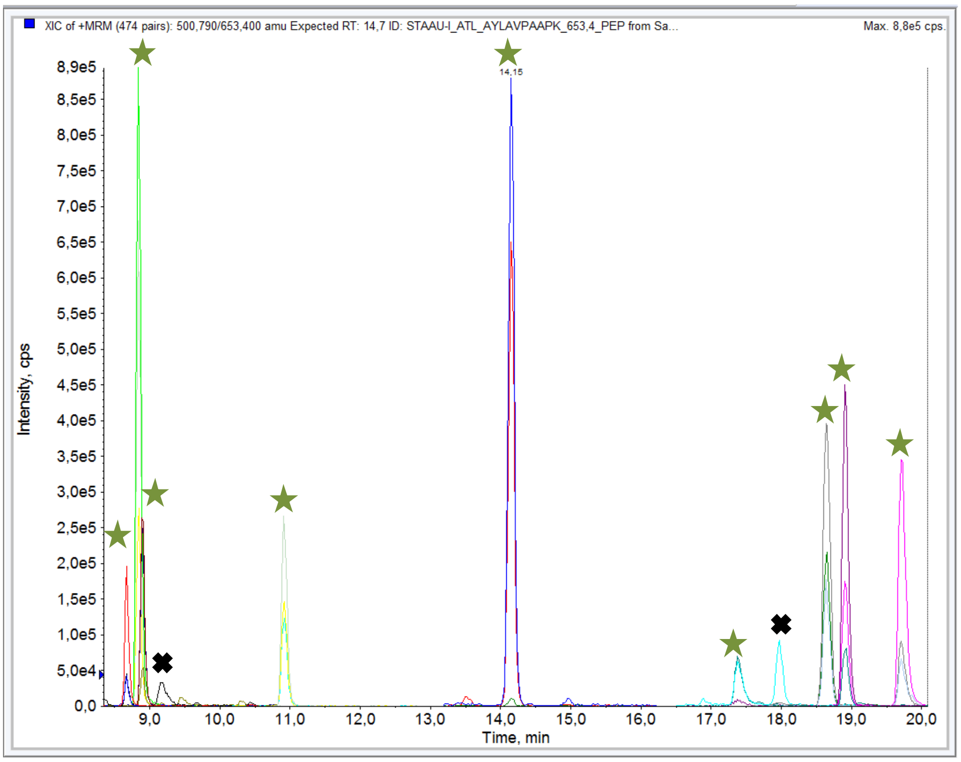

B - Extracted Ion chromatogram of peptides used for Penicillin Binding Protein 2a (PBP2a) detection.

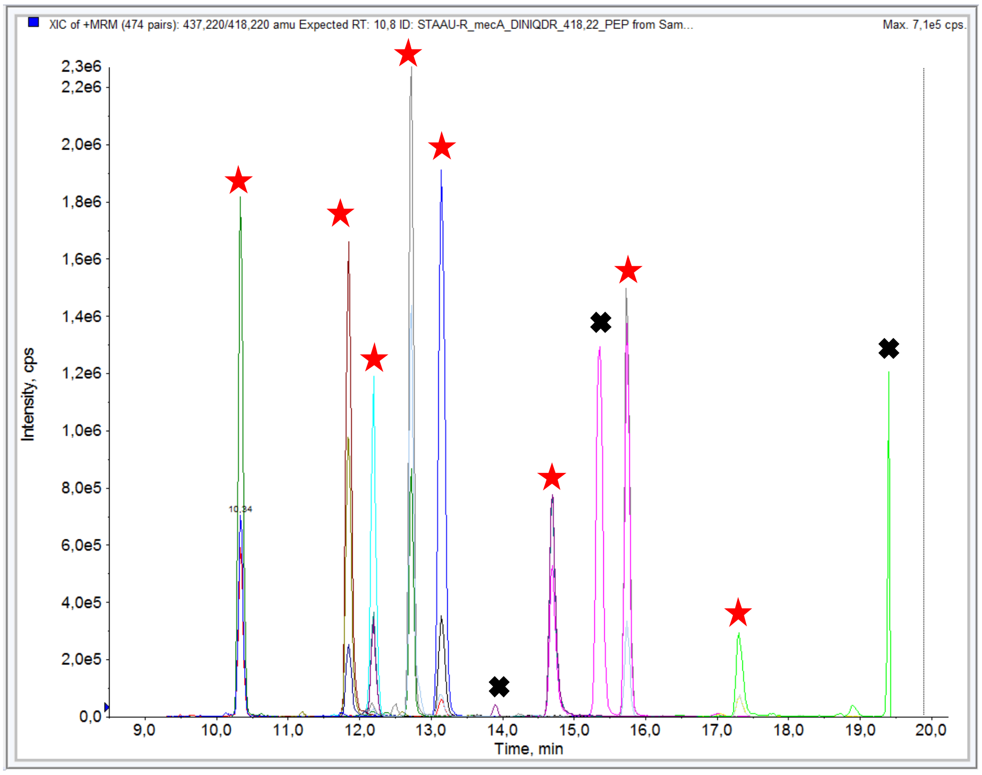

C - Extracted Ion chromatogram of peptides used for Penicillin Binding Protein 2c (PBP2c) detection.

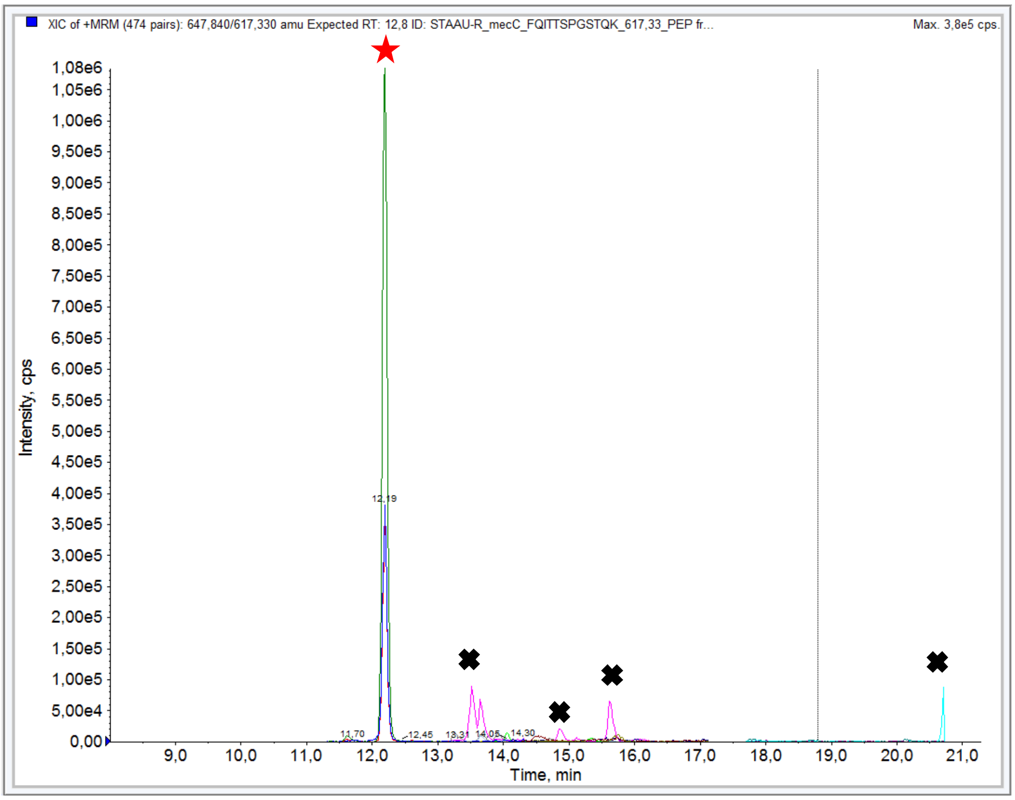

To be noted, one peptide is shared by PBP2a and PBP2c.

D - Extracted ion chromatogram of peptide used for Panton-Valentine Leukocidin (PVL) detection.

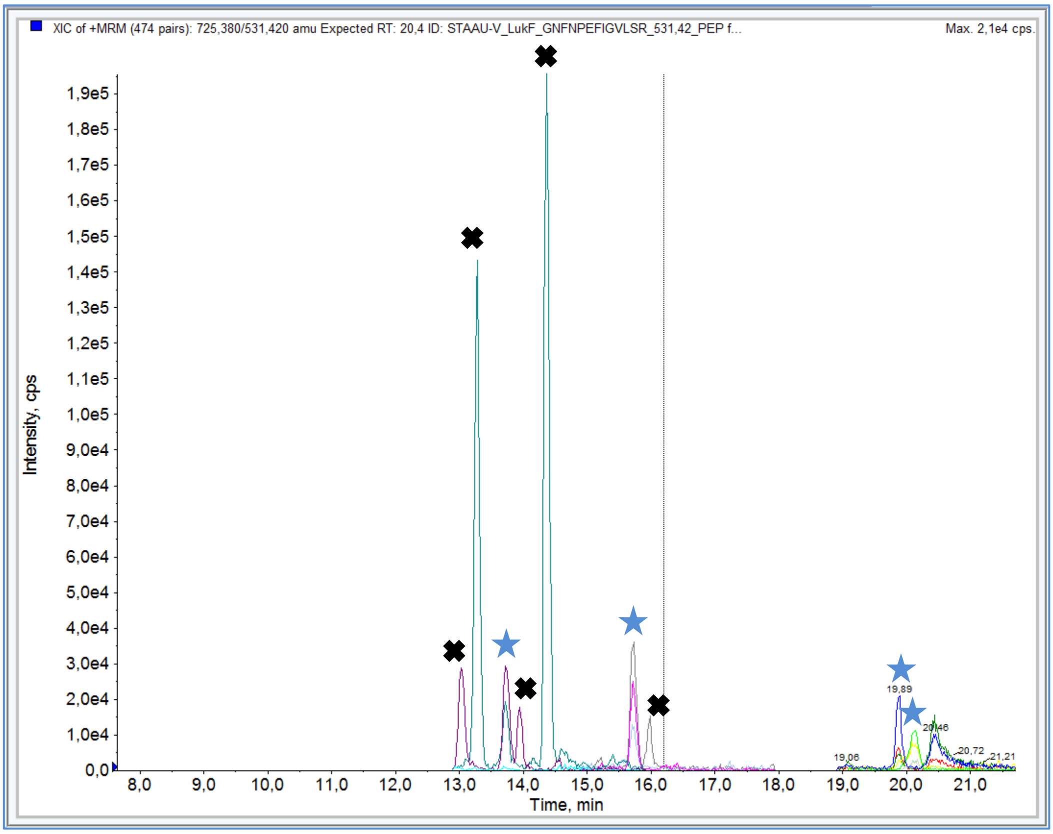

E - Extracted ion chromatogram of peptide used for Toxic Shock Syndrome Toxin 1 (TSST-1) detection.

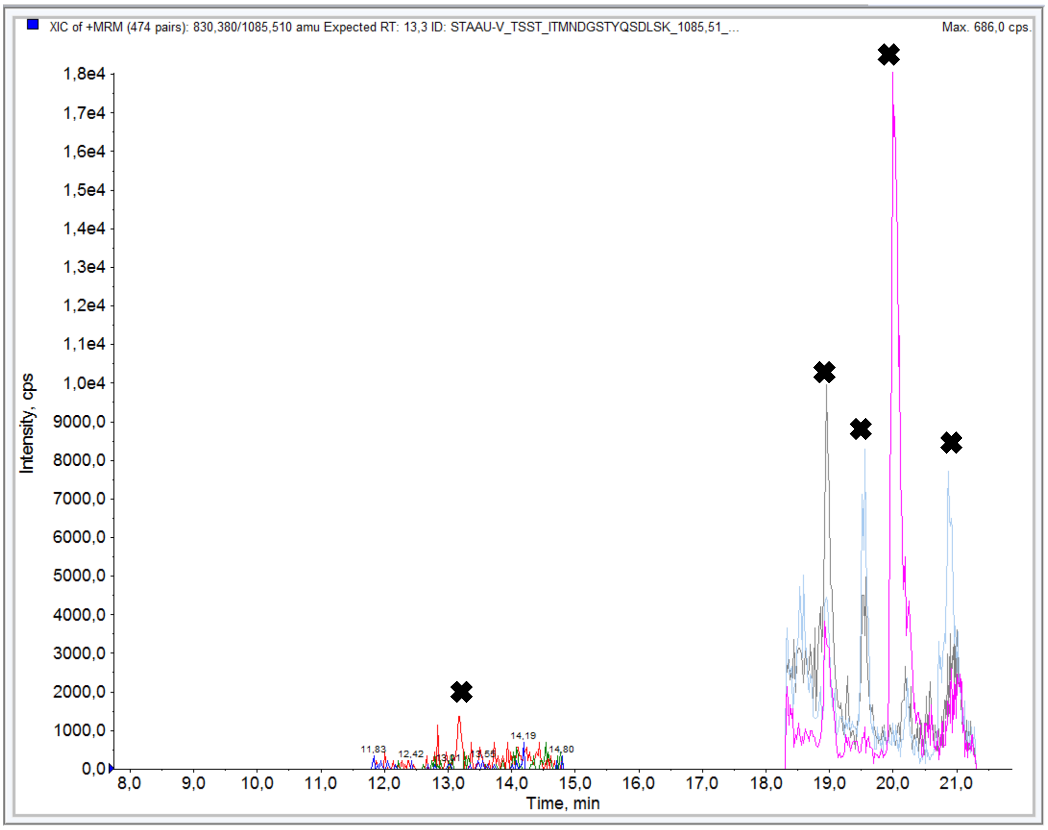

F - Total extracted ion chromatogram

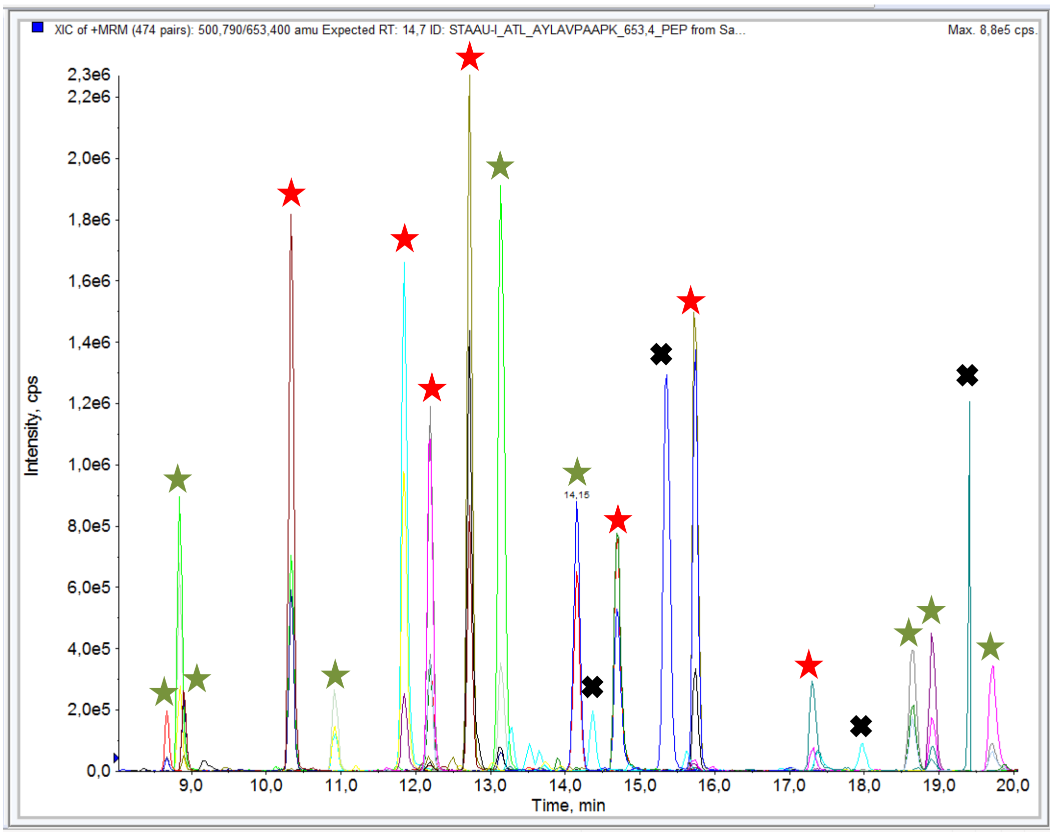

<u>Supplementary Figure 2:</u> Screenshot of Strain_080, analysed in blind condition, identified as S. aureus, expressing neither Penicillin Binding Protein 2a (PBP2a), Penicillin Binding Protein 2c (PBP2c), Panton-Valentine Leukocidin (PVL) nor Toxic Shock Syndrom Toxin 1 (TSST-1).

Peptides used for strain identification, resistance markers and virulence factors are identified by green, red and blue stars, respectively. Black crosses show isolated transitions.

A - Extracted ion chromatogram of peptides used for identification.

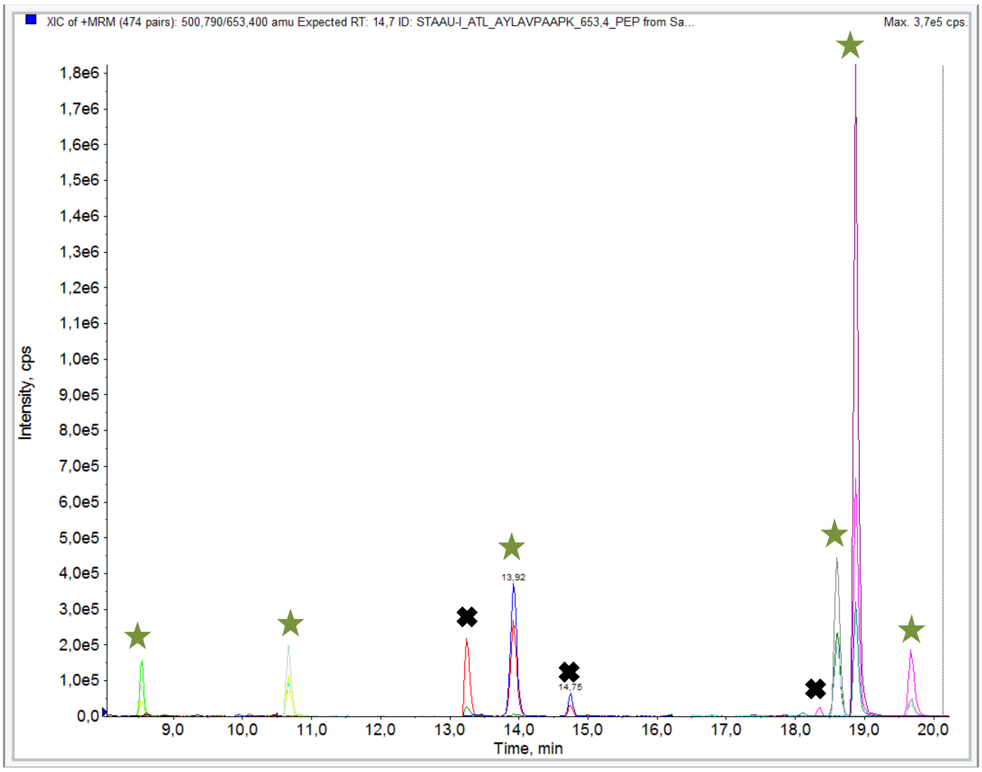

B - Extracted Ion chromatogram of peptides used for Penicillin Binding Protein 2a (PBP2a) detection.

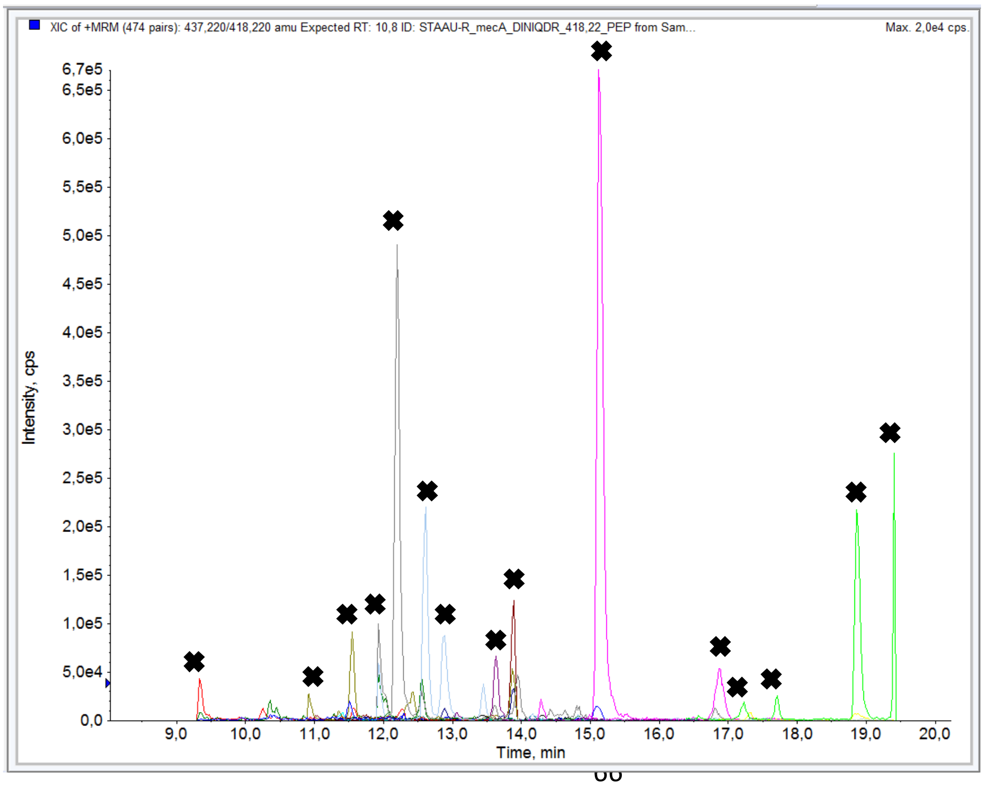

C - Extracted Ion chromatogram of peptides used for Penicillin Binding Protein 2c (PBP2c) detection.

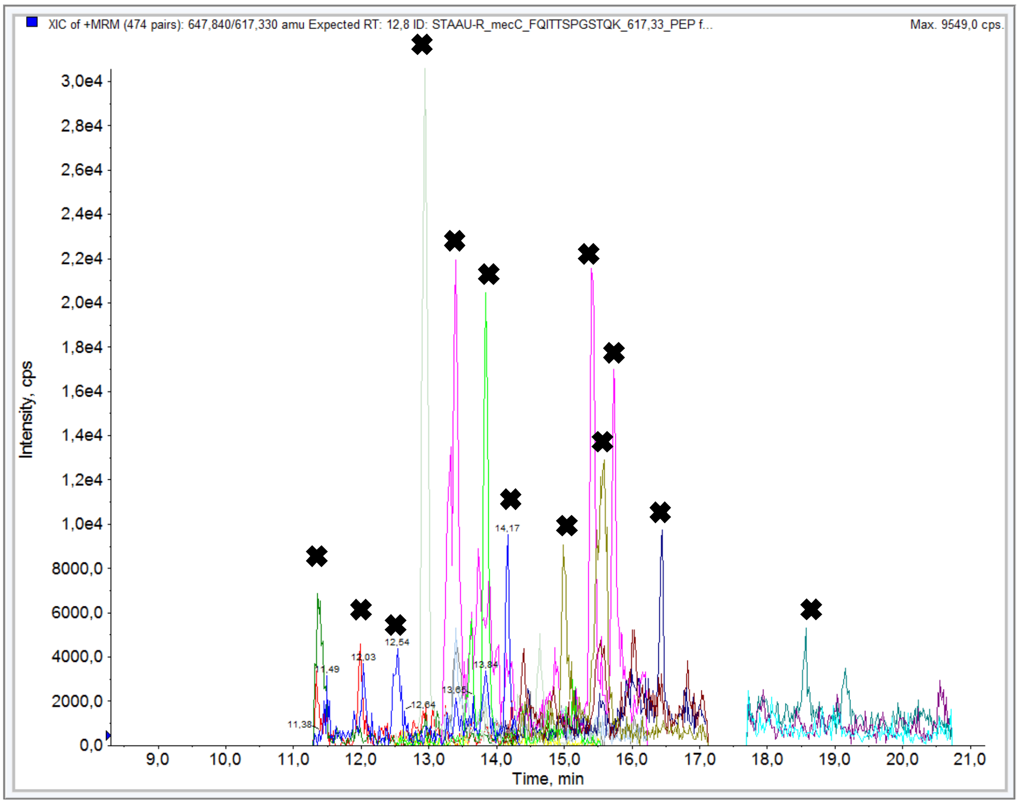

D - Extracted ion chromatogram of peptide used for Panton-Valentine Leukocidin (PVL) detection.

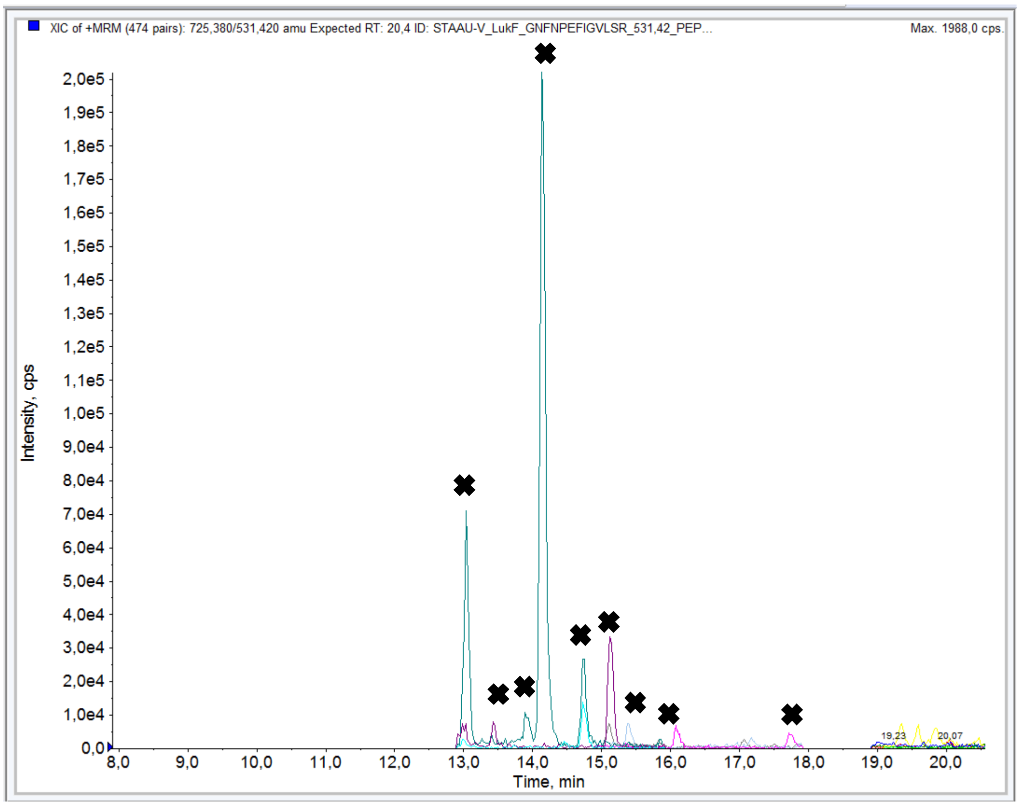

E - Extracted ion chromatogram of peptide used for Toxic Shock Syndrome Toxin 1 (TSST-1) detection.

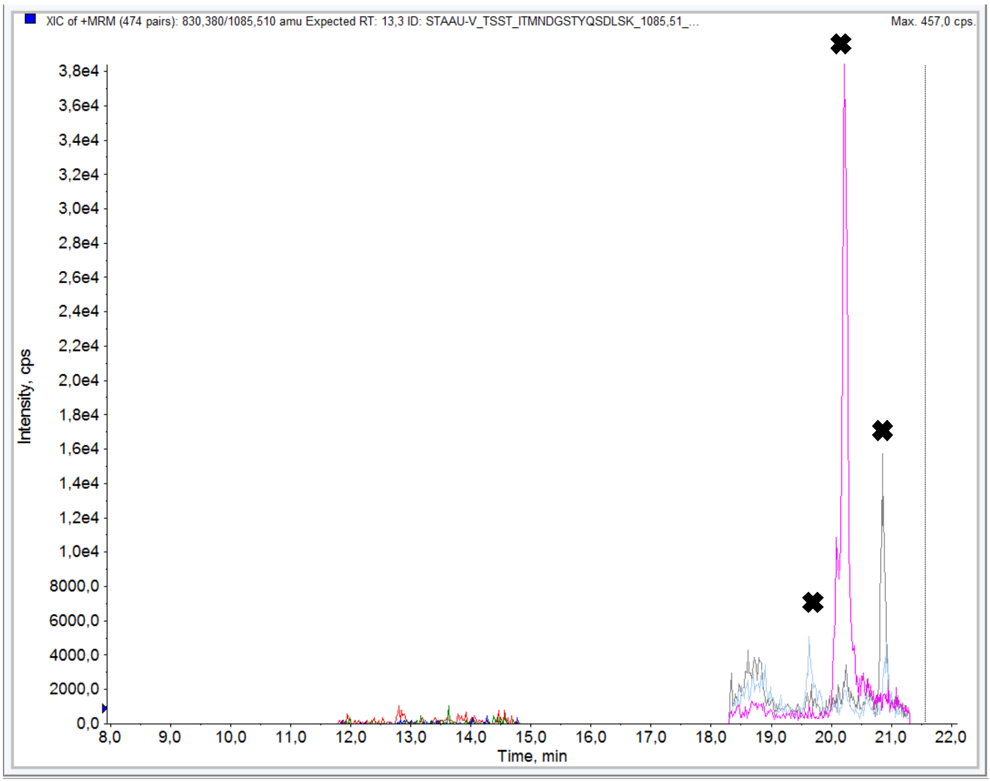

F - Total extracted ion chromatogram

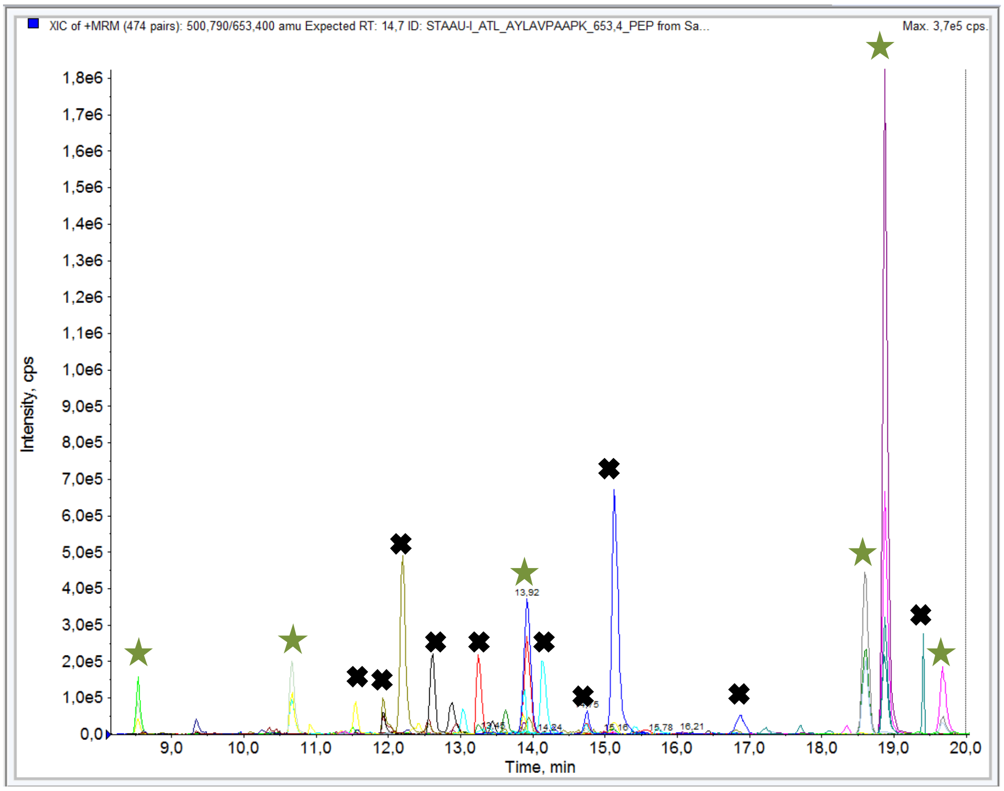

<u>Supplementary Figure 3:</u> Screenshot of Strain_040, analysed in blind conditions, and identified as Staphylococcus hominis and expressing neither Penicillin Binding Protein 2a (PBP2a) neither Penicillin Binding Protein 2c (PBP2c), nor Panton-Valentine Leukocidin (PVL), nor Toxic Shock Syndrome Toxin 1 (TSST-1).

Peptides used for strain identification, resistance markers and virulence factors are identified by green, red and blue stars, respectively. Black crosses show isolated transitions.

A - Extracted ion chromatogram of peptides used for identification.

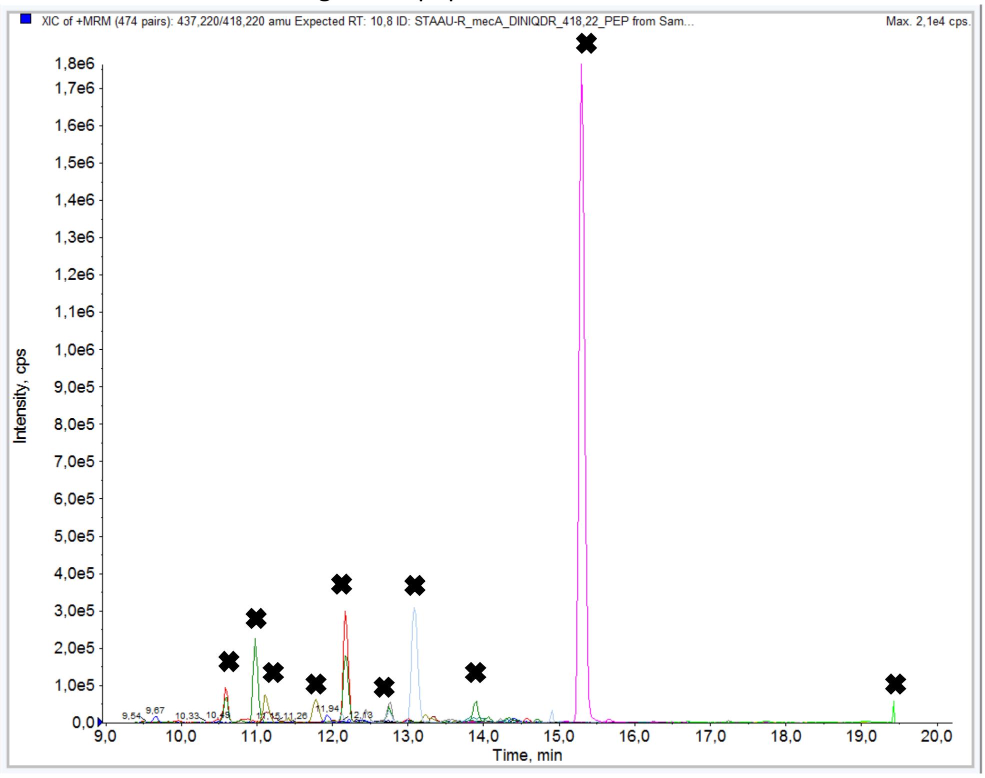

B - Extracted Ion chromatogram of peptides used for Penicillin Binding Protein 2a (PBP2a) detection.

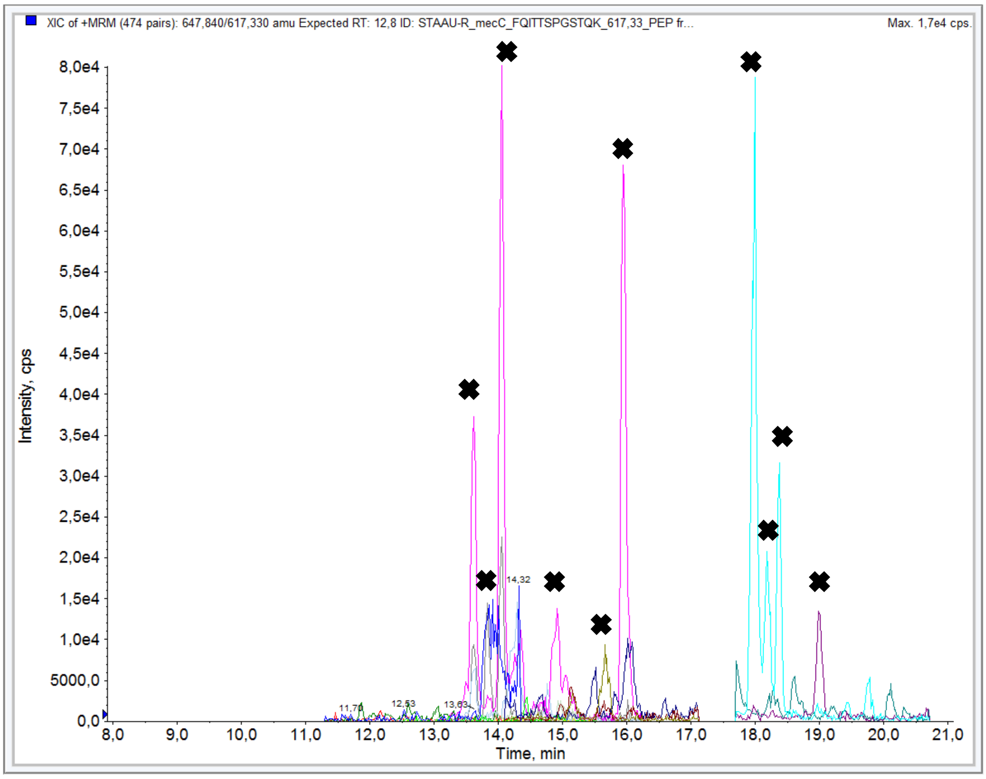

C - Extracted Ion chromatogram of peptides used for Penicillin Binding Protein 2c (PBP2c) detection.

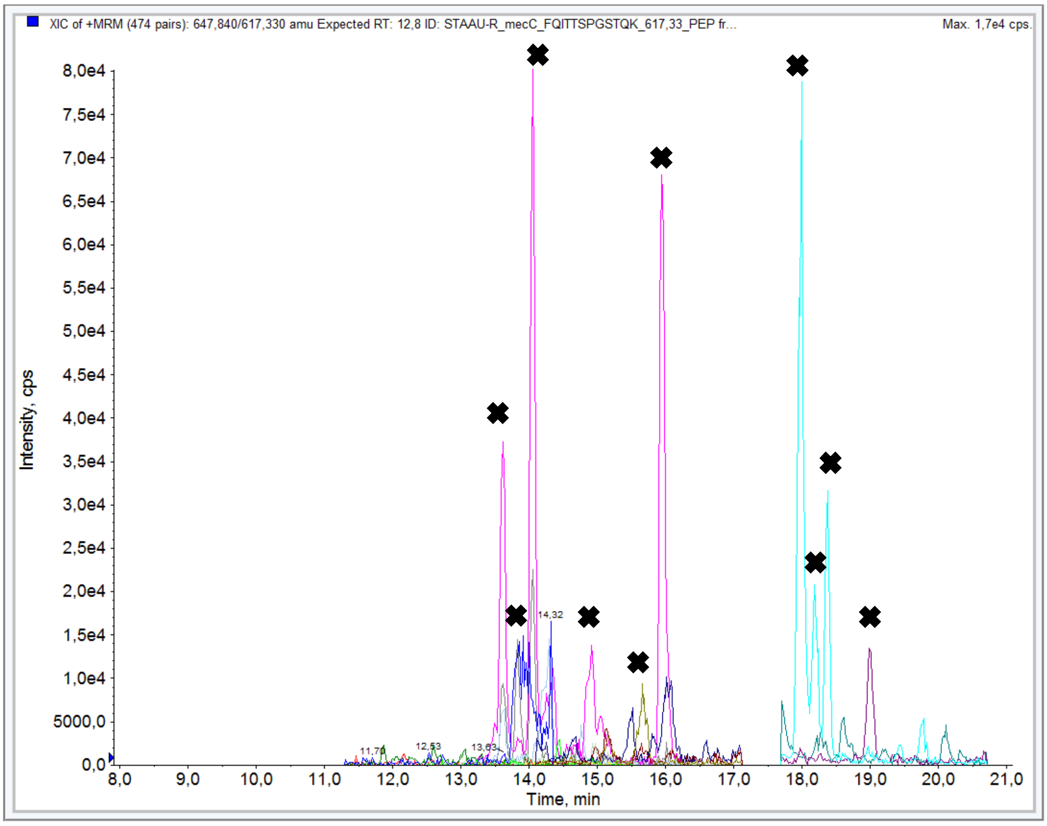

D - Extracted ion chromatogram of peptide used for Panton-Valentine Leukocidin (PVL) detection.

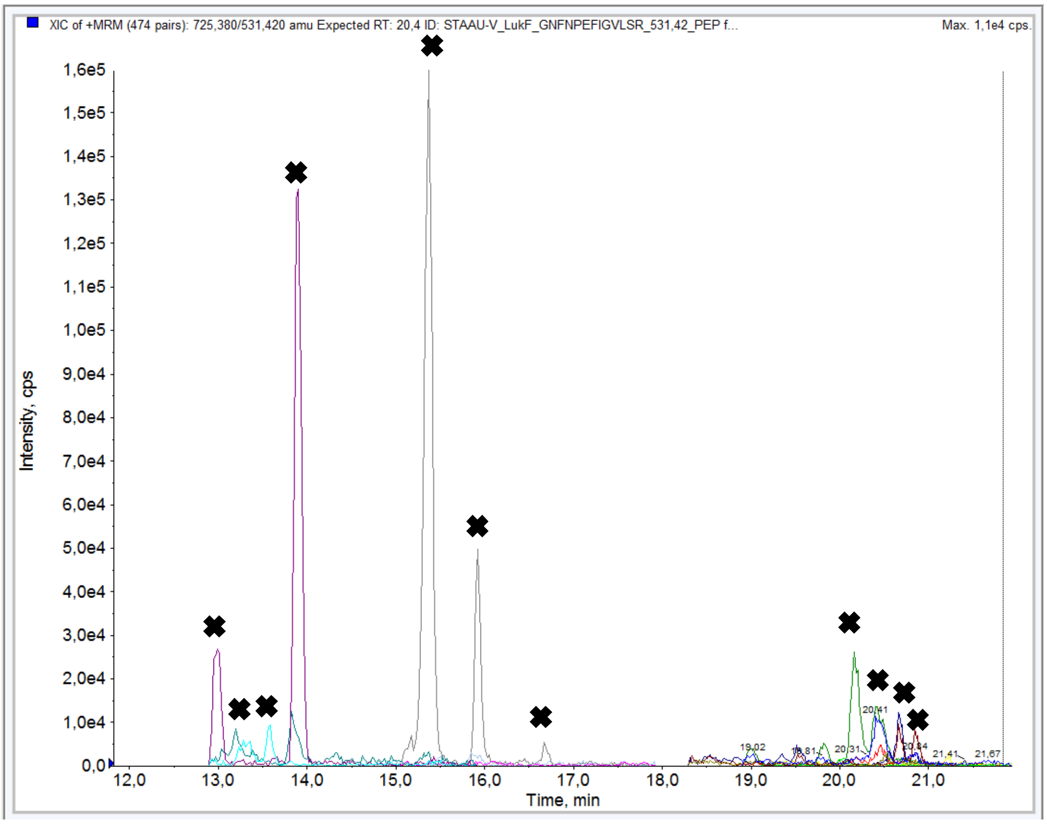

E - Extracted ion chromatogram of peptide used for Toxic Shock Syndrome Toxin 1 (TSST-1) detection.

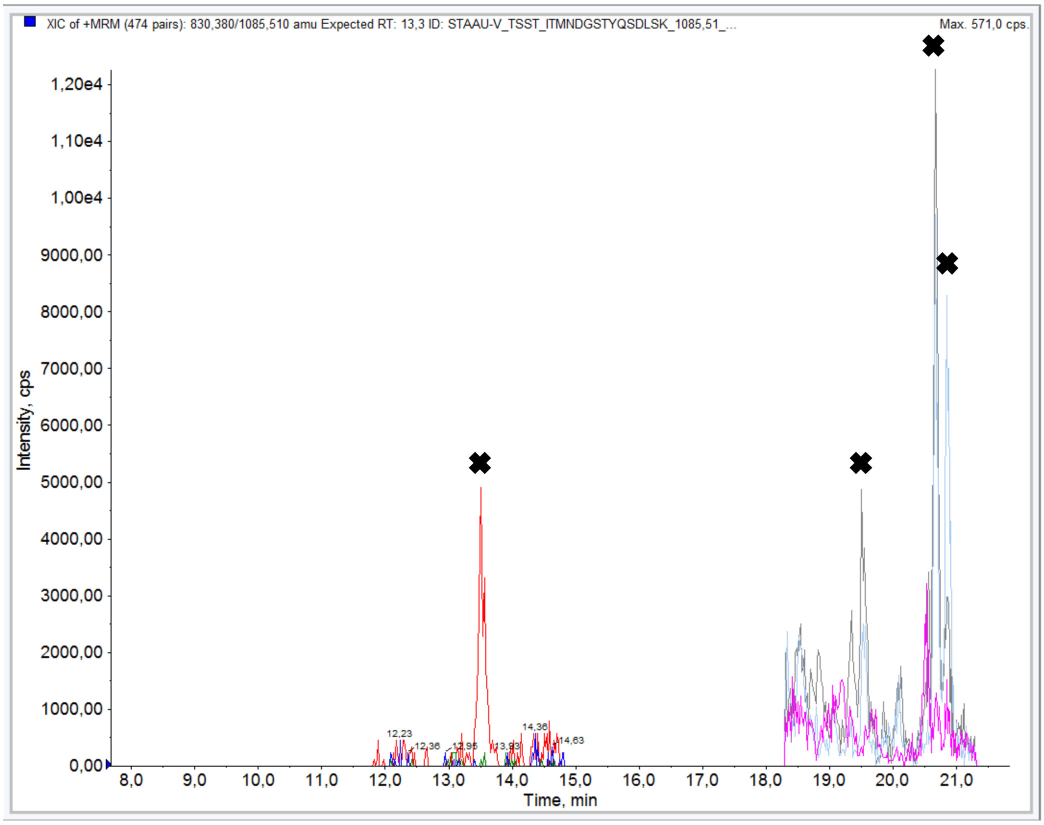

F: Total extracted ion chromatogram

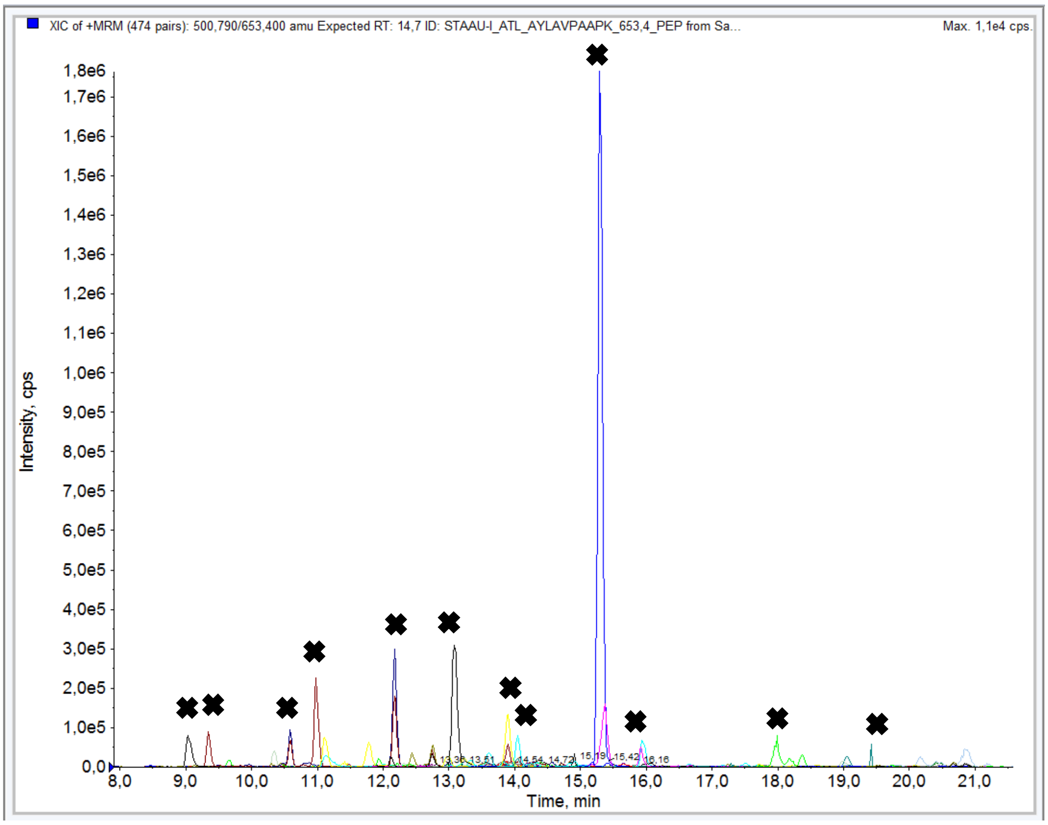

Supplementary Figure 4: Screenshot of Strain_071, analysed in blind conditions, and identified as Staphylococcus aureus, expressing Penicillin Binding Protein 2a (PBP2a) and Toxic Shock Syndrome Toxin 1 (TSST-1), but neither Penicillin Binding Protein 2c (PBP2c), nor Panton-Valentine Leukocidin (PVL).

Peptides used for strain identification, resistance markers and virulence factors are identified by green, red and blue stars, respectively. Black crosses show isolated transitions.

A - Extracted ion chromatogram of peptides used for identification

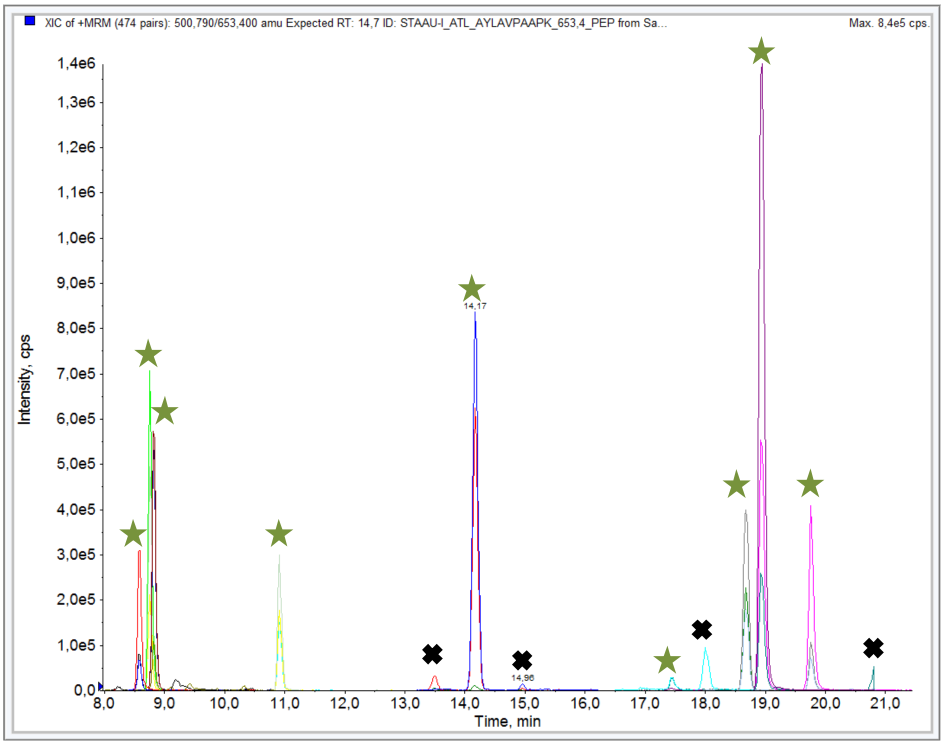

B - Extracted Ion chromatogram of peptides used for Penicillin Binding Protein 2a (PBP2a) detection.

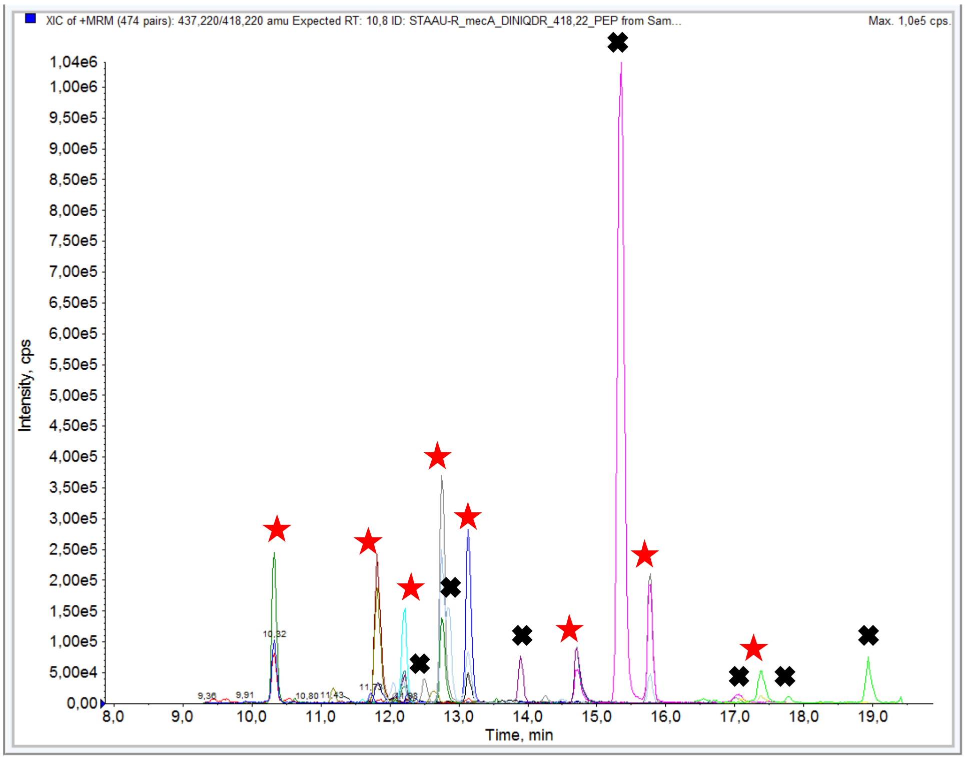

C - Extracted Ion chromatogram of peptides used for Penicillin Binding Protein 2c (PBP2c) detection.

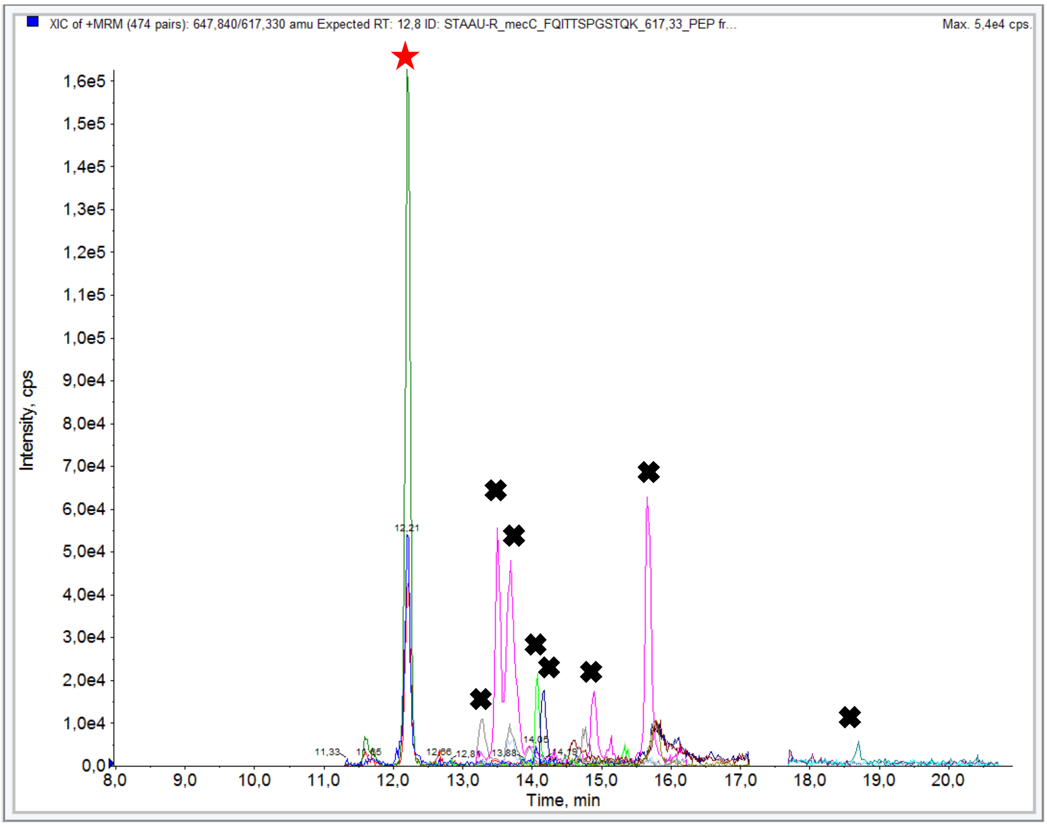

To be noted, one peptide is shared by PBP2a and PBP2c.

D - Extracted ion chromatogram of peptide used for Panton-Valentine Leukocidin (PVL) detection.

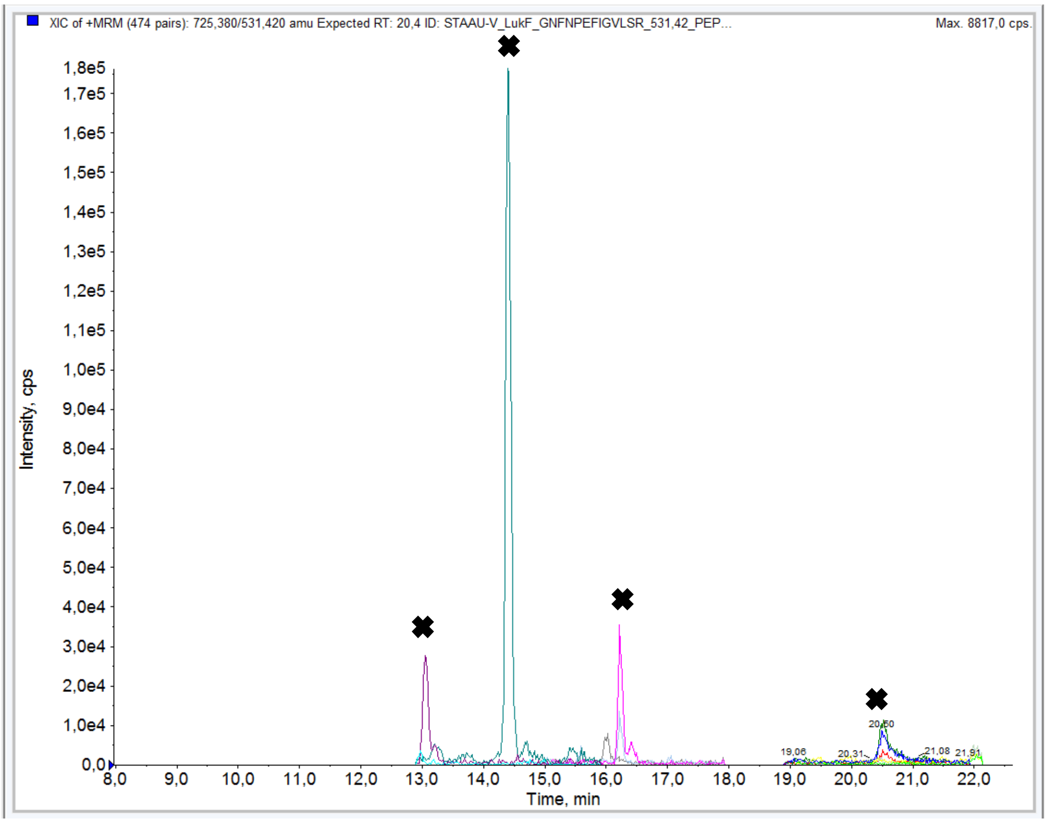

E - Extracted ion chromatogram of peptide used for Toxic Shock Syndrome Toxin 1 (TSST-1) detection.

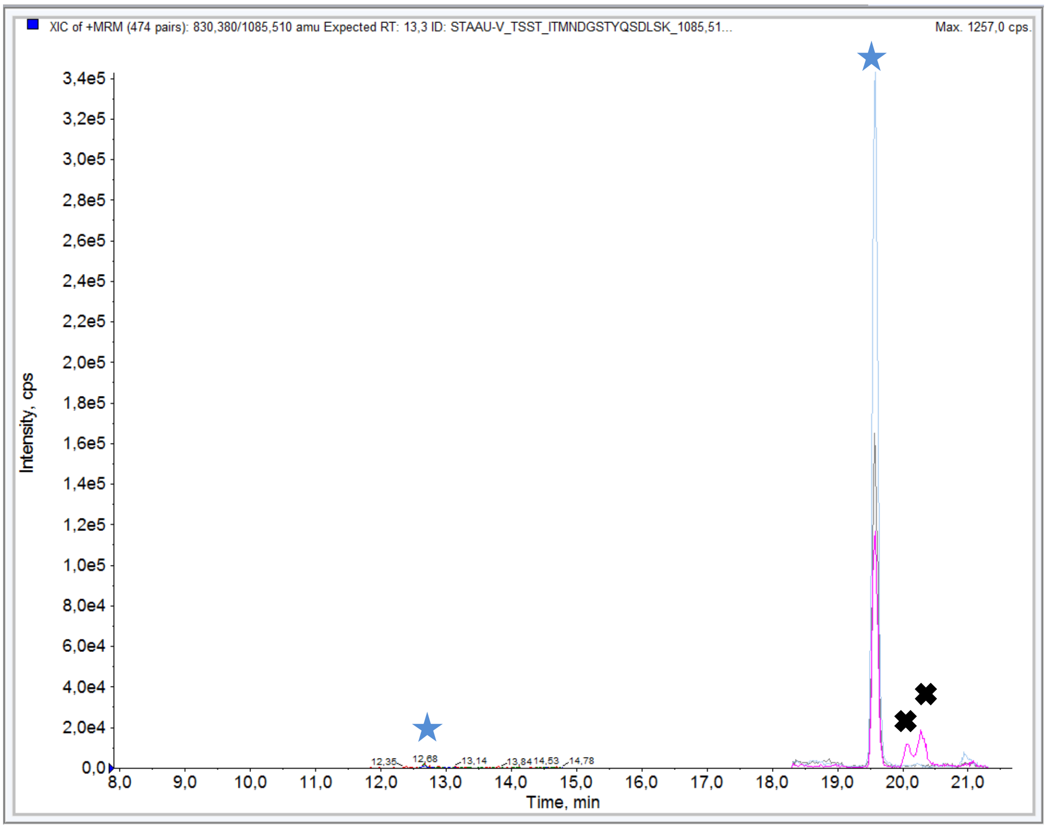

F: - Total extracted ion chromatogram

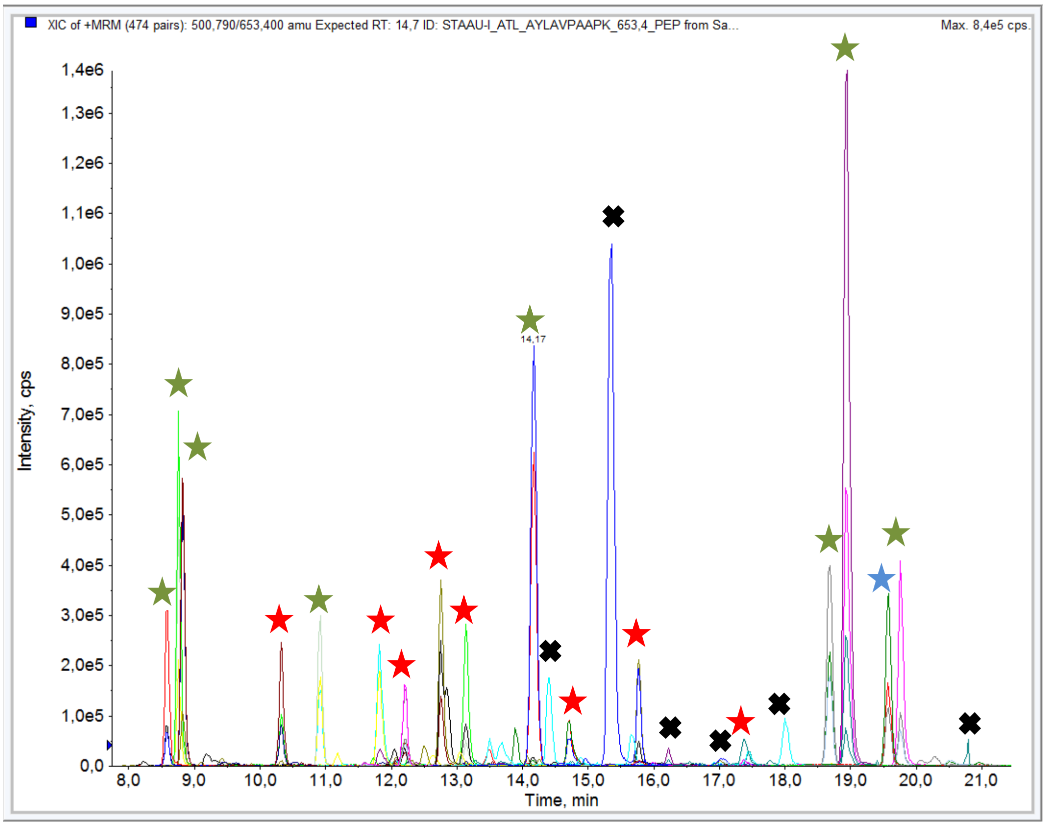

<u>Supplementary Figure 5:</u> Screenshot of Strain_078, analysed in blind conditions, and identified as Staphylococcus aureus, expressing Penicillin Binding Protein 2c (PBP2c) but neither Penicillin Binding Protein 2a (PBP2a), nor Panton-Valentine Leukocidin (PVL), nor Toxic Shock Syndrome Toxin 1 (TSST-1).

Peptides used for strain identification, resistance markers and virulence factors are identified by green, red and blue stars, respectively. Black crosses show isolated transitions.

A - Extracted ion chromatogram of peptides used for identification

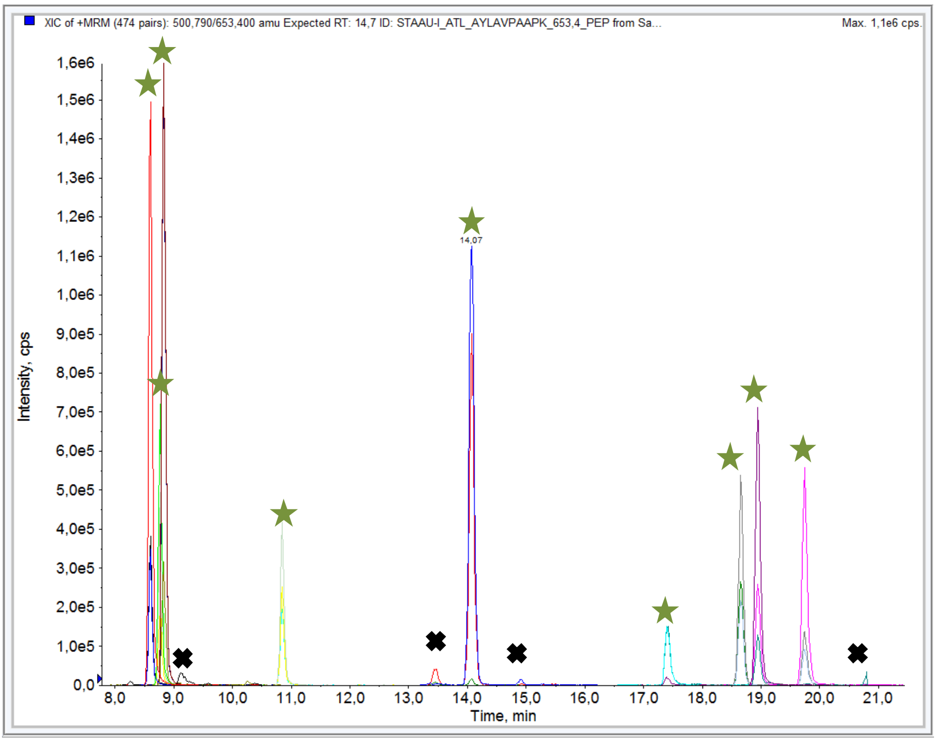

B - Extracted Ion chromatogram of peptides used for Penicillin Binding Protein 2a (PBP2a) detection.

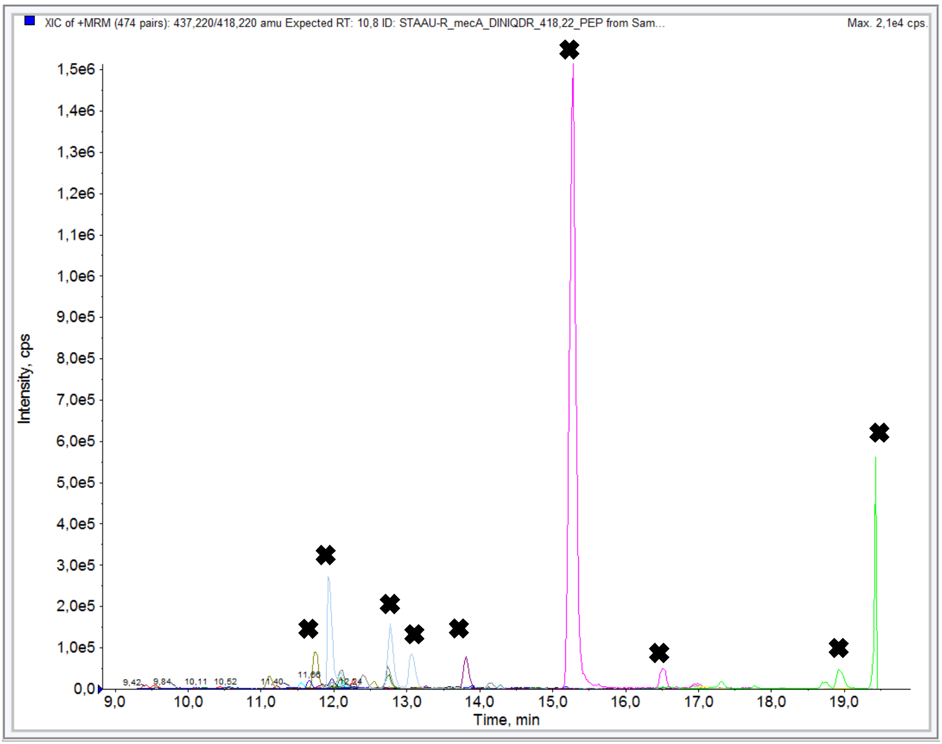

C - Extracted Ion chromatogram of peptides used for Penicillin Binding Protein 2c (PBP2c) detection.

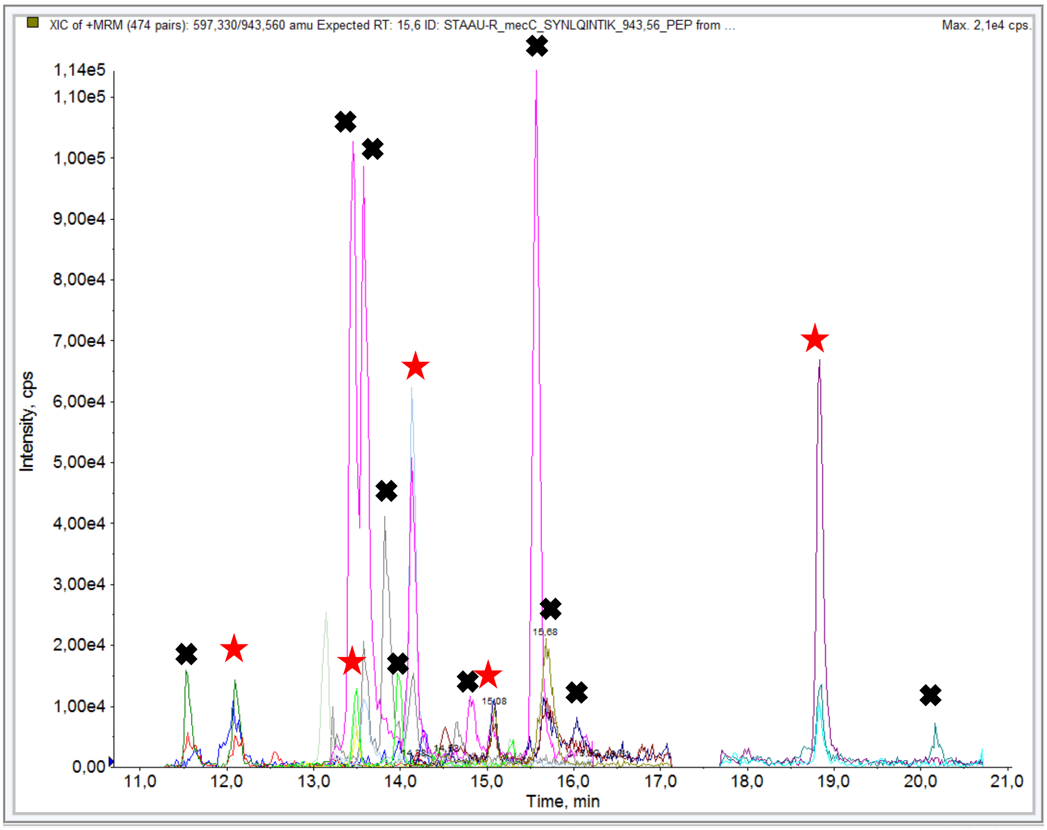

D - Extracted ion chromatogram of peptide used for Panton-Valentine Leukocidin (PVL) detection.

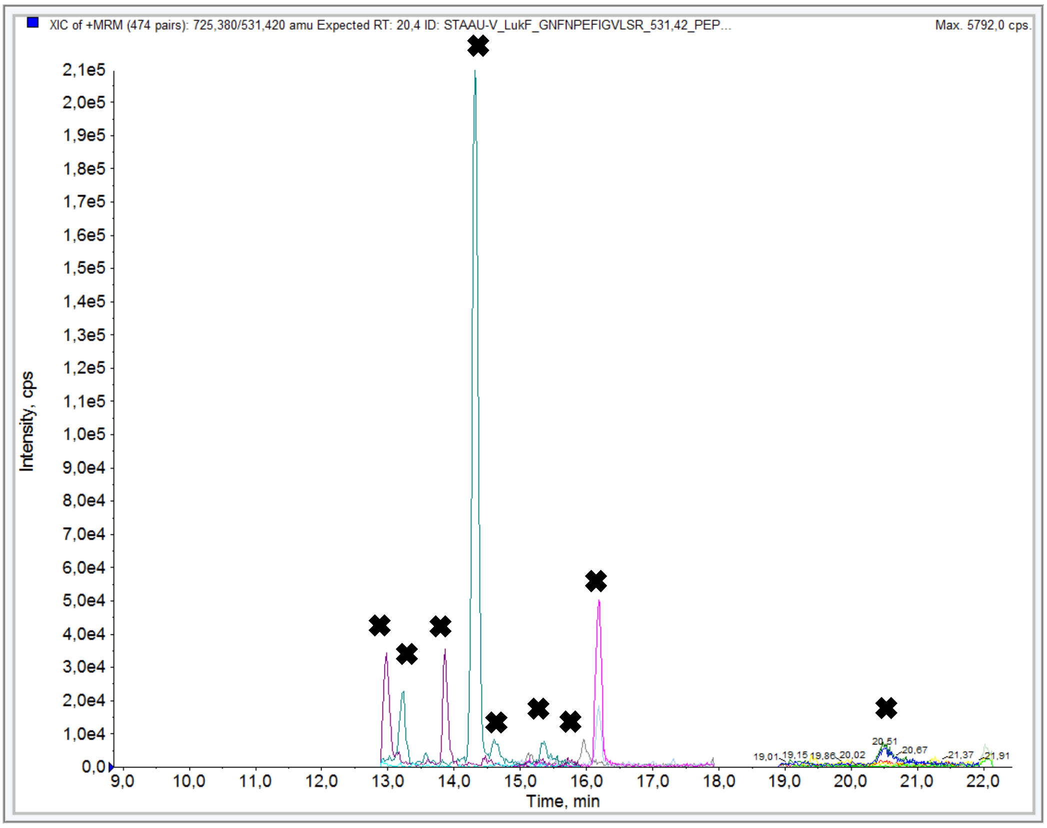

E - Extracted ion chromatogram of peptide used for Toxic Shock Syndrome Toxin 1 (TSST-1) detection.

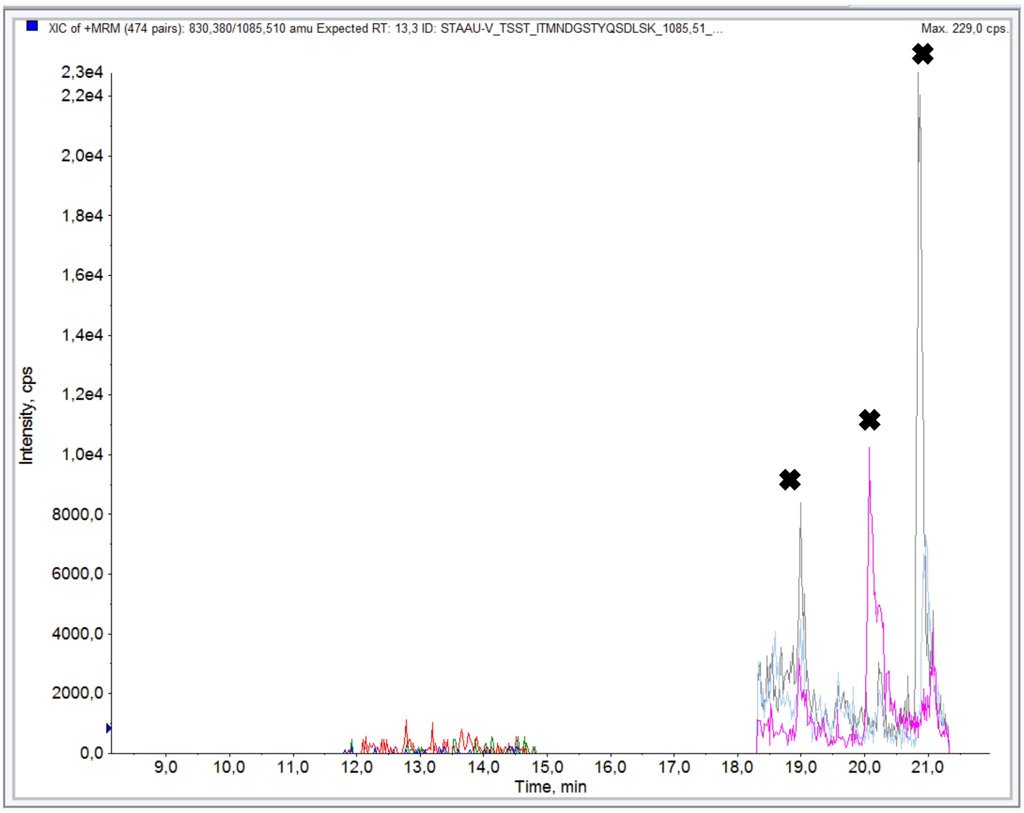

F - Total extracted ion chromatogram.

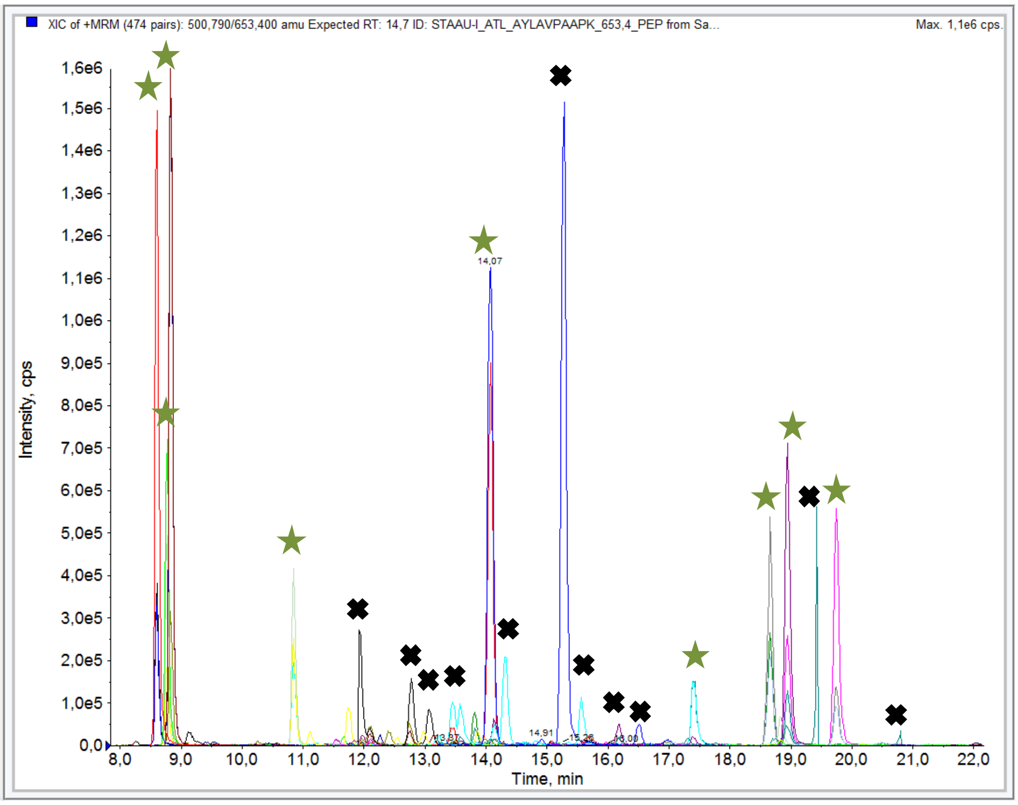

Supplementary Figure 6: Frequency distribution of the coefficient of variation from the normalised area ratios across the training set

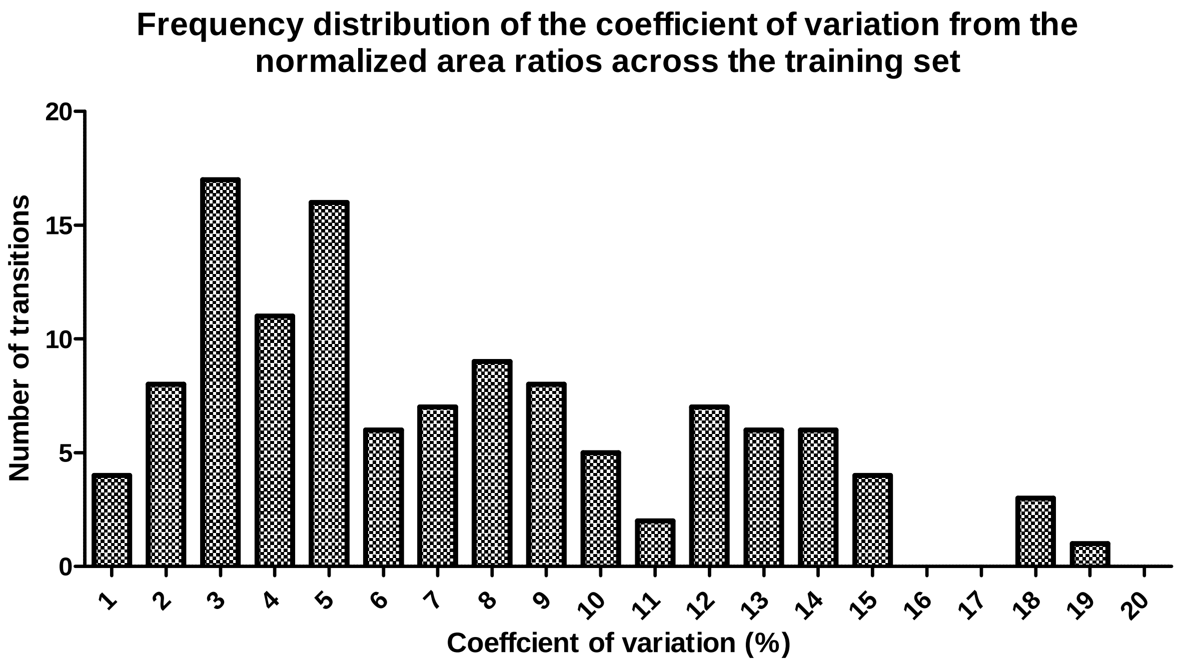

<u>Supplementary Figure 7:</u> feature detection in Strain_093

Supplementary Figure 7a: S. aureus identification based on peptide ratio conservation.

Nine *S. aureus* specific I-peptides were selected for identification. Each I-peptide was included when transitions retention time standard deviation were lower than 0.03 and when transitions areas were higher than 4000. If one of these criteria was not respected for one transition, the I-peptide was considered as undetected. For each peptide, 3 transitions (T1, T2 and T3) were used. For each transition a proportion can be calculated in every sample.

$$\text{Area ratio (T1)} = \frac{\text{T1 area}}{(\text{T1 area} + \text{T2 area} + \text{T3 area})}$$

For each transition, an average area was calculated based on positive samples from the train set, which allowed to determine an average ratio for each transition. This average ratio was compared to an unknown sample ratio. The slope and the r^2 were calculated from the resulting linear regression curve. Strain_093 (test set) gave the following graph:

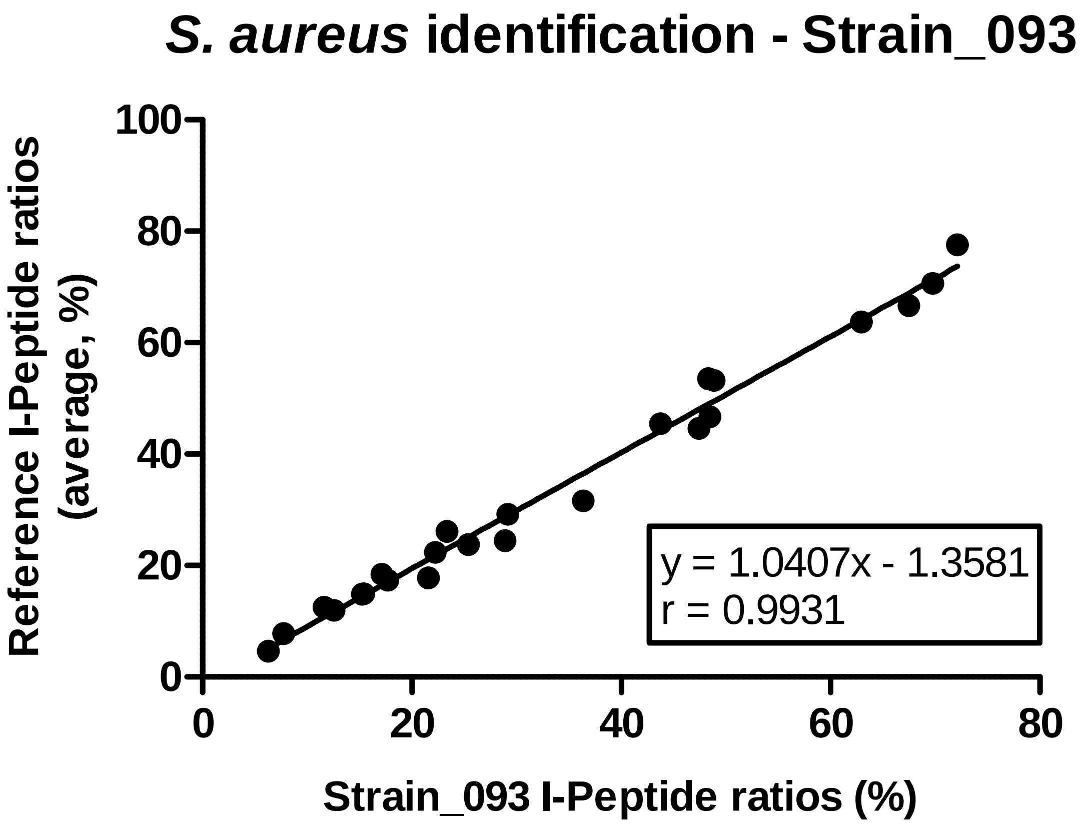

Supplementary Figure 7b: Penicillin Binding Protein 2a (PBP2a) detection based on peptide ratio conservation.

Transition areas were compared with average transition area obtained with positive samples from the train set. The slope and the r^2 were calculated from the resulting linear regression curve. Strain_093 gave the following graph:

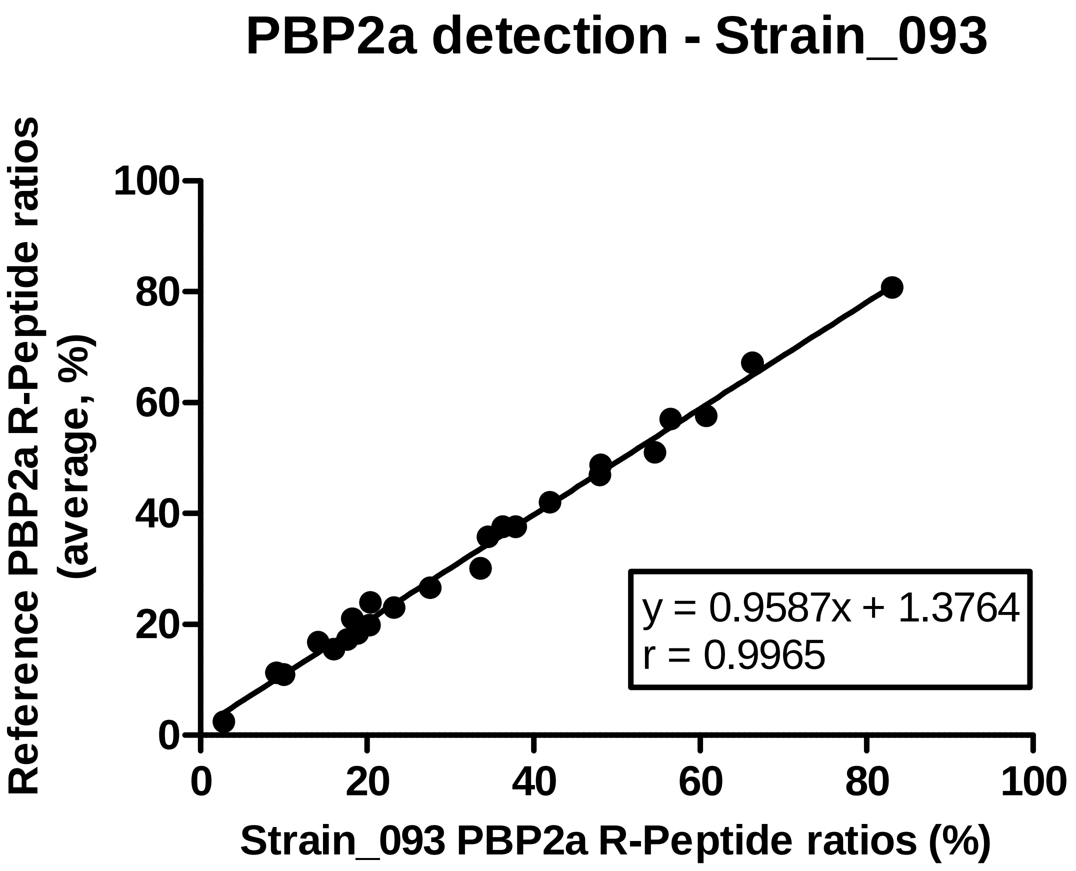

Supplementary Figure 7c: Panton-Valentine Leukocidin (PVL) detection based on peptide ratio conservation.

Transition areas were compared with average transition area obtained with positive samples from the train set. The slope and the r² were calculated from the resulting linear regression curve. Strain_093 gave the following graph:

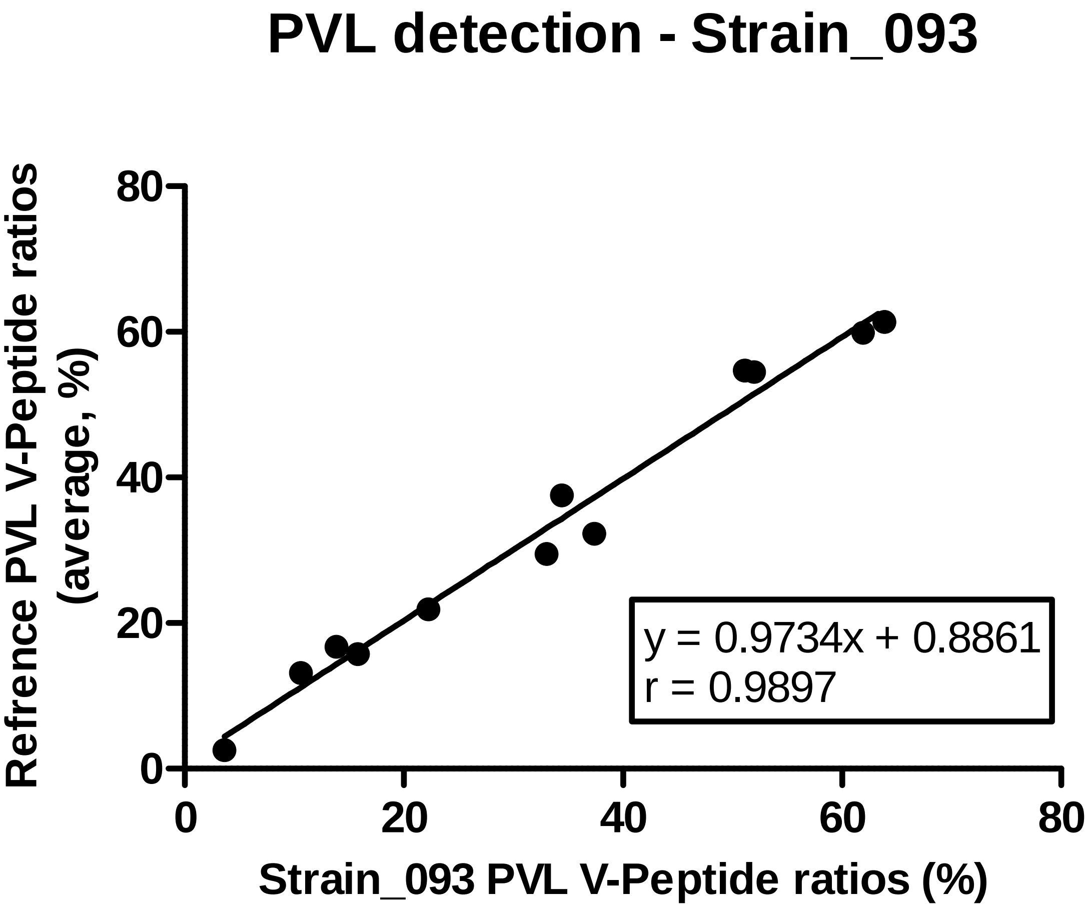

<u>Supplementary Figure 8:</u> Slope and the Pearson product-moment correlation coefficients (r) from the linear regression across the data sets

Supplementary Figure 8a: *S. aureus* identification

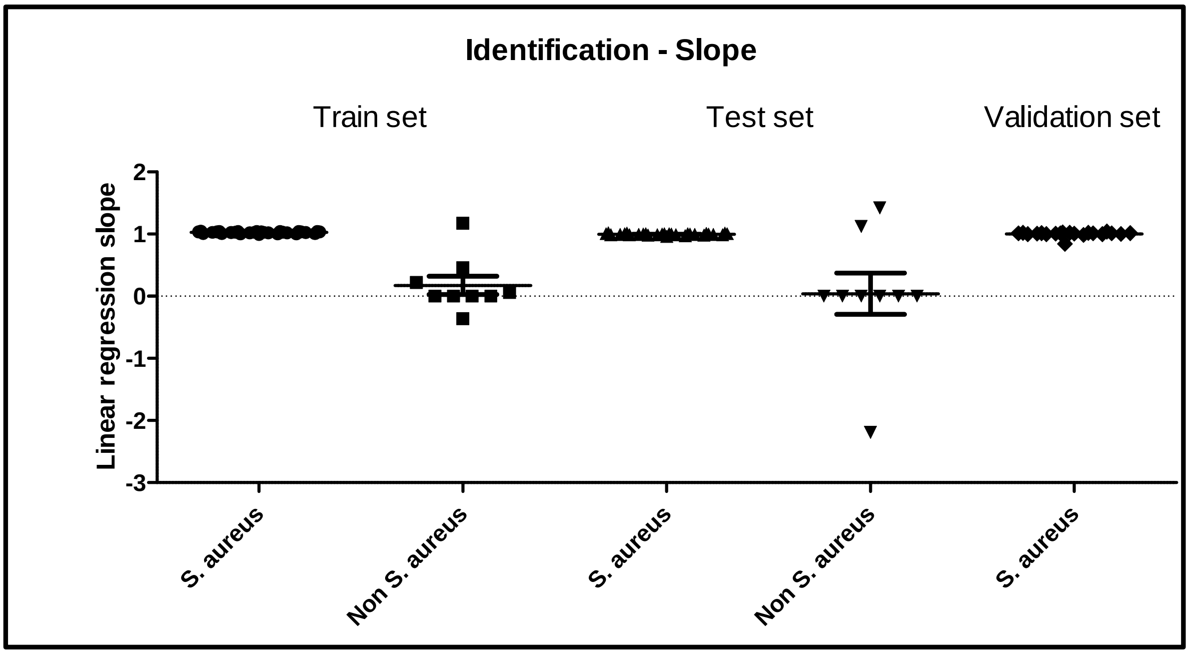

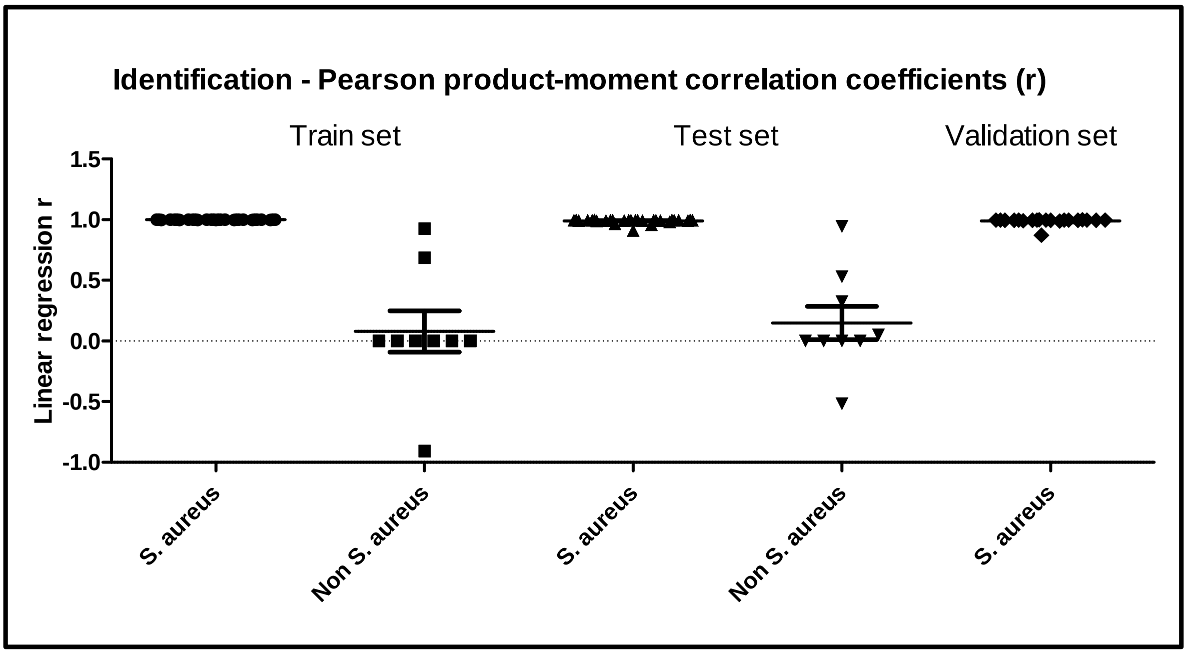

Supplementary Figure 8b: Penicillin Binding Protein 2c (PBP2c) detection.

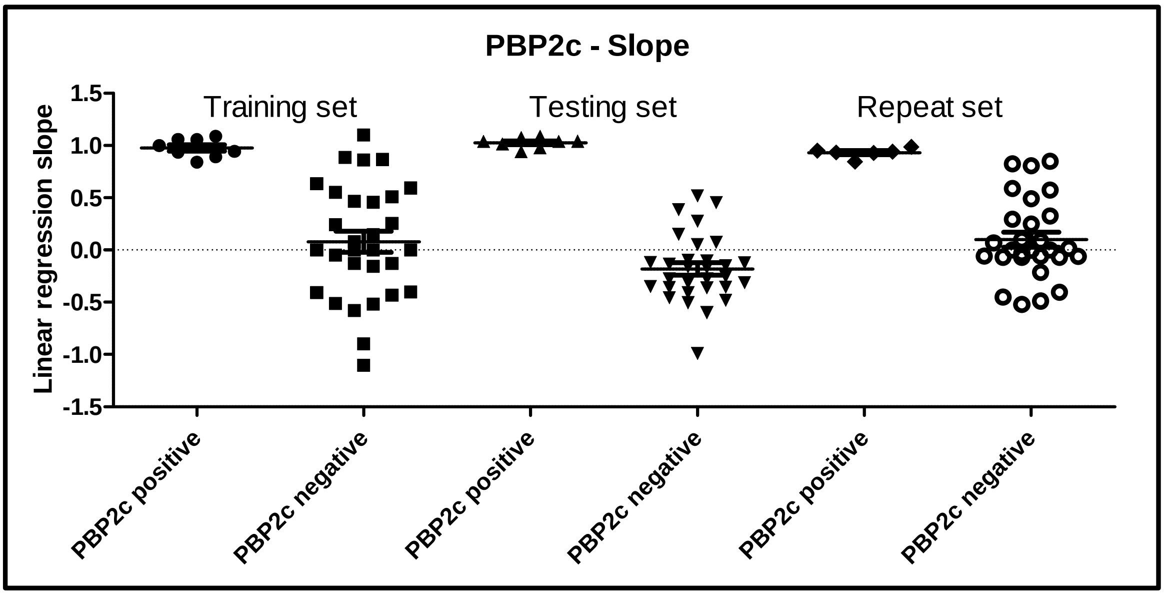

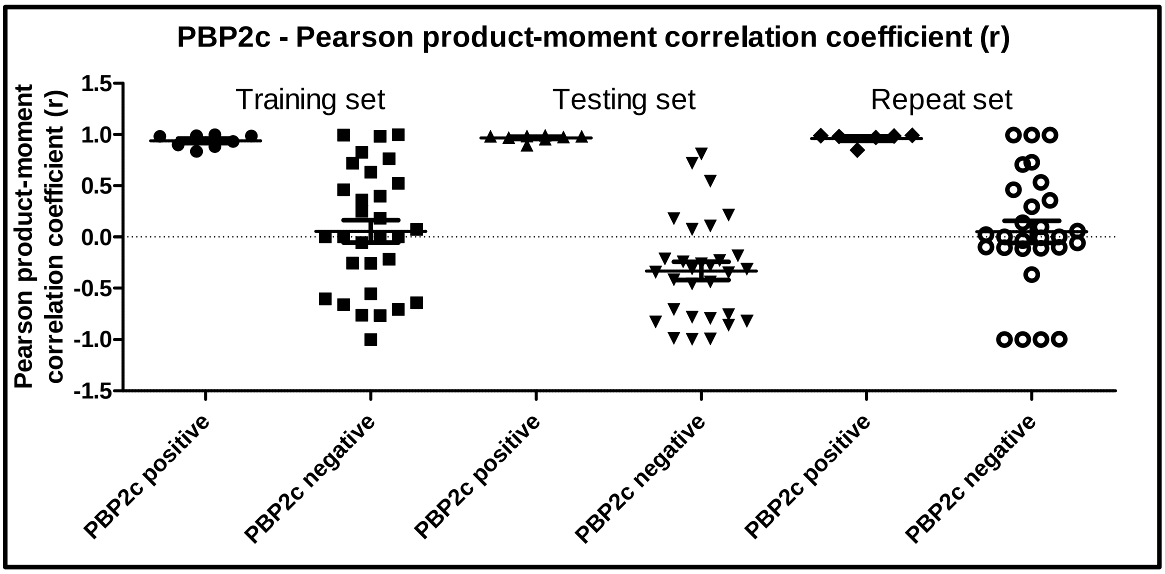

Peptide FQITTSPGSTQK is shared by PBP2a and PBP2c encoded, respectively, by the mecA and mecC genes. This peptide was use to detected both proteins. When PBP2a was produced, FQITTSPGSTQK detection lead to both a slope and a Pearson product-moment correlation coefficient (r) superior to 0.7, but the total number of detected peptide was never superior than 2. The latest rule enabled a clear distinction between PBP2c and PBP2a production.

Supplementary Figure 8c: Panton-Valentine Leukocidin (PVL) detection.

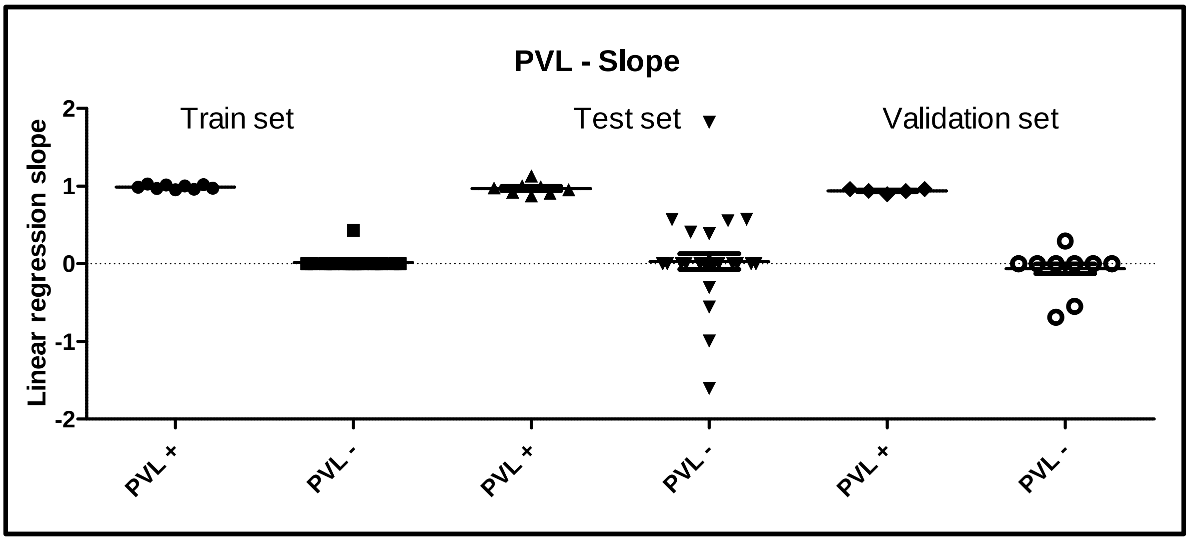

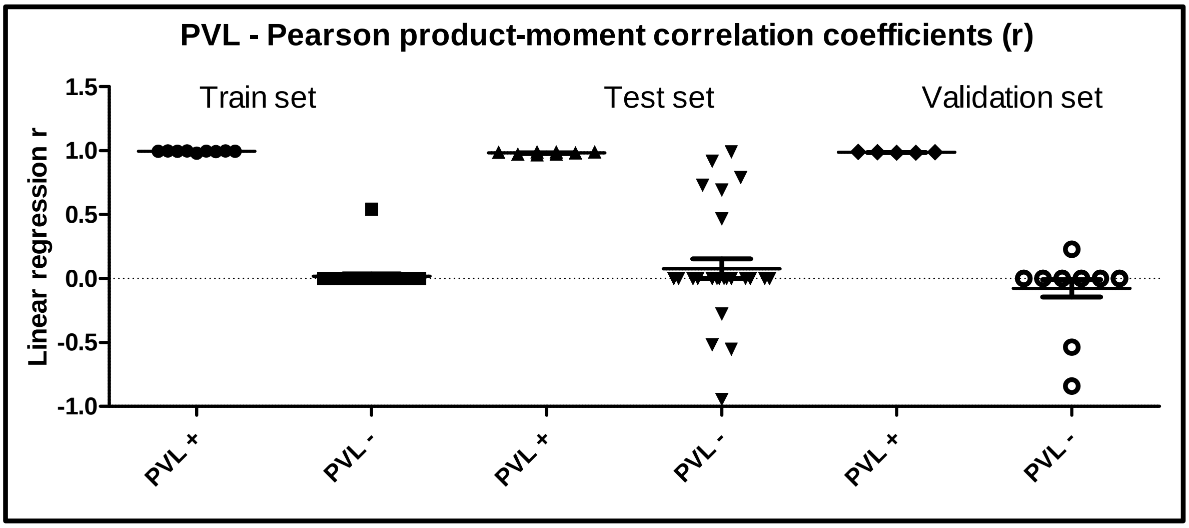

References

1. Ito, T. *et al.* Guidelines for reporting novel mecA gene homologues. *Antimicrob. Agents Chemother.* **56**, 4997-4999 (2012).

2. Tristan, A. *et al.* Global distribution of Panton-Valentine leukocidin--positive methicillin-resistant Staphylococcus aureus, 2006. *Emerg. Infect. Dis.* **13**, 594-600 (2007).

3. Perret, M. *et al.* Cross-talk between Staphylococcus aureus leukocidins-intoxicated macrophages and lung epithelial cells triggers chemokine secretion in an inflammasome-dependent manner. *Cell Microbiol.* **14**, 1019-1036 (2012).

4. Rasigade, J.P. *et al.* Global distribution and evolution of Panton-Valentine leukocidin-positive methicillin-susceptible Staphylococcus aureus, 1981-2007. *J. Infect. Dis.* **201**, 1589-1597 (2010).

5. Dauwalder, O. *et al.* Epidemiology of invasive methicillin-resistant Staphylococcus aureus clones collected in France in 2006 and 2007. *J. Clin. Microbiol.* **46**, 3454-3458 (2008).

6. Blanco, R. *et al.* Molecular epidemiology of Panton-Valentine leukocidin-positive Staphylococcus aureus in Spain: emergence of the USA300 clone in an autochthonous population. *J. Clin. Microbiol.* **49**, 433-436 (2011).

7. Monecke, S., Luedicke, C., Slickers, P. & Ehricht, R. Molecular epidemiology of Staphylococcus aureus in asymptomatic carriers. *Eur. J. Clin. Microbiol. Infect. Dis.* **28**, 1159-1165 (2009).

8. Chroboczek, T. *et al.* Clonal complex 398 methicillin susceptible Staphylococcus aureus: a frequent unspecialized human pathogen with specific phenotypic and genotypic characteristics. *PLoS. One.* **8**, e68462-(2013).

9. Laurent, F. *et al.* MRSA harboring mecA variant gene mecC, France. *Emerg. Infect. Dis.* **18**, 1465-1467 (2012).

10. Oliveira, D.C., Tomasz, A. & De Lencastre, H. Secrets of success of a human pathogen: molecular evolution of pandemic clones of meticillin-resistant Staphylococcus aureus. *Lancet Infect. Dis.* **2**, 180-189 (2002).

11. Grundmann, H. *et al.* Geographic distribution of Staphylococcus aureus causing invasive infections in Europe: a molecular-epidemiological analysis. *PLoS. Med.* **7**, e1000215-(2010).

12. Wannet, W.J. *et al.* Widespread dissemination in The Netherlands of the epidemic berlin methicillin-resistant Staphylococcus aureus clone with low-level resistance to oxacillin. *J. Clin. Microbiol.* **42**, 3077-3082 (2004).

13. Enright, M.C., Day, N.P., Davies, C.E., Peacock, S.J. & Spratt, B.G. Multilocus sequence typing for characterization of methicillin-resistant and methicillin-susceptible clones of Staphylococcus aureus. *J. Clin. Microbiol.* **38**, 1008-1015 (2000).

14. Harmsen, D. *et al.* Typing of methicillin-resistant Staphylococcus aureus in a university hospital setting by using novel software for spa repeat determination and database management. *J. Clin. Microbiol.* **41**, 5442-5448 (2003).
